# Geometric Interpretation of Gene Coexpression Network Analysis

**DOI:** 10.1371/journal.pcbi.1000117

**Published:** 2008-08-15

**Authors:** Steve Horvath, Jun Dong

**Affiliations:** Department of Human Genetics, David Geffen School of Medicine, and Department of Biostatistics, School of Public Health, University of California, Los Angeles, California, United States of America; University of Tokyo, Japan

## Abstract

The merging of network theory and microarray data analysis techniques has spawned a new field: gene coexpression network analysis. While network methods are increasingly used in biology, the network vocabulary of computational biologists tends to be far more limited than that of, say, social network theorists. Here we review and propose several potentially useful network concepts. We take advantage of the relationship between network theory and the field of microarray data analysis to clarify the meaning of and the relationship among network concepts in gene coexpression networks. Network theory offers a wealth of intuitive concepts for describing the pairwise relationships among genes, which are depicted in cluster trees and heat maps. Conversely, microarray data analysis techniques (singular value decomposition, tests of differential expression) can also be used to address difficult problems in network theory. We describe conditions when a close relationship exists between network analysis and microarray data analysis techniques, and provide a rough dictionary for translating between the two fields. Using the angular interpretation of correlations, we provide a geometric interpretation of network theoretic concepts and derive unexpected relationships among them. We use the singular value decomposition of module expression data to characterize approximately factorizable gene coexpression networks, i.e., adjacency matrices that factor into node specific contributions. High and low level views of coexpression networks allow us to study the relationships among modules and among module genes, respectively. We characterize coexpression networks where hub genes are significant with respect to a microarray sample trait and show that the network concept of intramodular connectivity can be interpreted as a fuzzy measure of module membership. We illustrate our results using human, mouse, and yeast microarray gene expression data. The unification of coexpression network methods with traditional data mining methods can inform the application and development of systems biologic methods.

## Author Summary

Similar to natural languages, network language is ever evolving. While some network terms (concepts) are widely used in gene coexpression network analysis, others still need to be developed to meet the ever increasing demand for describing the system of gene transcripts. There is a need to provide an intuitive geometric explanation of network concepts and to study their relationships. For example, we show that certain seemingly disparate network concepts turn out to be synonyms in the context of coexpression modules. We show how coexpression network language affects our understanding of biology. For example, there are geometric reasons why highly connected hub genes in important coexpression modules tend to be important, and why hub genes in one module cannot be hubs in another distinct module. We provide a short dictionary for translating between microarray data analysis language and network theory language to facilitate communication between the two fields. We describe several examples that illustrate how the two data analysis fields can inform each other.

## Introduction

Many biological networks share topological properties. Common global properties include modular organization [1],[2], the presence of highly connected hub nodes, and approximate ‘scale free topology’ [3],[4]. Common local topological properties include the presence of recurring patterns of interconnections (‘network motifs’) in regulation networks [5]–[7].

One goal of this article is to describe existing and novel network concepts (also known as network statistics or indices [8]) that can be used to describe local and global network properties. For example, the clustering coefficient [9] is a network concept, which measures the cohesiveness of the neighborhood of a node. We are particularly interested in network concepts that are defined with regard to a ‘gene significance measure’. Gene significance measures are of great practical importance since they allow one to incorporate external gene information into the network analysis. In functional enrichment analysis, a gene significance measure could indicate pathway membership. In gene knock-out experiments, gene significance could indicate knock-out essentiality. We study gene significance measures since a microarray sample trait (e.g., case control status) gives rise to a statistical measure of gene significance. For example, the Student *t*-test of differential expression leads to a gene significance measure. Many traditional microarray data analysis methods focus on the relationship between the microarray sample trait and the gene expression data. For example, gene filtering methods aim to find a list of (differentially expressed) genes that are significantly associated with the microarray sample trait; another example are microarray-based prediction methods that aim to accurately predict the sample trait on the basis of the gene expression data.

Gene expression profiles across microarray samples can be highly correlated and it is natural to describe their pairwise relations using network language. Genes with similar expression patterns may form complexes, pathways, or participate in regulatory and signaling circuits [10]–[12]. Gene coexpression networks have been used to describe the transcriptome in many organisms, e.g., yeast, flies, worms, plants, mice, and humans [13]–[23]. Gene coexpression network methods have also been used for typical microarray data analysis tasks such as gene filtering [19], [24]–[26] and outcome prediction [27],[28].

While the utility of network methods for analyzing microarray data has been demonstrated in numerous publications, the utility of microarray data analysis techniques for solving network theoretic problems has not yet been fully appreciated. One goal of this article is to show that simple geometric arguments can be used to derive network theoretic results if the networks are defined on the basis of a correlation matrix.

### Definition of Gene Coexpression Networks

Although many of our network concepts will be useful for general networks, we are particularly interested in gene coexpression networks (also known as association-, influence-, relevance-, or correlation networks). Gene coexpression networks are built on the basis of a gene coexpression measure. The network nodes correspond to genes—or more precisely to gene expression profiles. The *i*th gene expression profile *x_i_* is a vector whose components report the gene expression values across *m* microarrays. We define the coexpression similarity *s_ij_* between genes *i* and *j* as the absolute value of the correlation coefficient between their expression profiles:
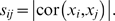



Using a thresholding procedure, this coexpression similarity is transformed into a measure of connection strength (adjacency). An unweighted network adjacency *a_ij_* between gene expression profiles *x_i_* and *x_j_* can be defined by hard thresholding the coexpression similarity *s_ij_* as follows
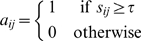
(1)where τ is the “hard” threshold parameter. Thus, two genes are linked (*a_ij_* = 1) if the absolute correlation between their expression profiles exceeds the (hard) threshold τ. Hard thresholding of the correlation leads to simple network concepts (e.g., the gene connectivity equals the number of direct neighbors) but it may lead to a loss of information: if τ has been set to 0.8, there will be no link between two genes if their correlation equals 0.799. To preserve the continuous nature of the coexpression information, one could simply define a weighted adjacency matrix as the absolute value of the gene expression correlation matrix, i.e., [*a_ij_*] = [*s_ij_*]. However, since microarray data can be noisy and the number of samples is often small, we and others have found it useful to emphasize strong correlations and to punish weak correlations. It is natural to define the adjacency between two genes as a power of the absolute value of the correlation coefficient [19],[24]:
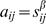
(2)with *β*≥1. This soft thresholding approach leads to a weighted gene coexpression network. We present empirical results for weighted and unweighted networks in the main text, Text S1, Text S2, and Text S3.

### Social Network Analogy: Affection Network

Since humans are organized into social networks, social network analogies should be intuitive to many readers. Therefore, we will refer to the following ‘affection network’ throughout this article. Assume that *n* individuals filled out an interest questionnaire, which was used to define a pairwise similarity score *s_ij_*. For convenience, we assume that the similarity measure takes on values between 0 and 1. Our definition of the affection network is based on the following assumption: the more similar the interests between two individuals, the more affection they feel for each other. More specifically, we assume that the affection (adjacency) *a_ij_* between two individuals is proportional to their similarity on a logarithmic scale, i.e.,
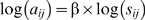
(3)This is equivalent to our soft thresholding approach *a_ij_* = *s_ij_^β^* (Equation 2). A soft threshold *β* = 2 implies that the affection *a_ij_* equals 0.25 if the similarity *s_ij_* equals 0.5.

## Results

### Gene Significance Based on a Microarray Sample Trait

Many network applications use at least one gene significance measure. Abstractly speaking, we define a gene significance measure as a function *GS* that assigns a nonnegative number to each gene; the higher *GS_i_* the more *biologically* significant is gene *i*. We assume that the minimum gene significance is 0. For example, if a statistical significance level (*p*-value) is available for each gene, the gene significance of the *i*th gene can be defined as minus log of the *p*-value, i.e., *GS_i_* = −log(*p_i_*). In this article, we are particularly interested in gene significance measures that are based on a microarray sample trait, e.g., a clinical outcome. The microarray sample trait *T* = (*T*_1_,…,*T_m_*) may be quantitative (e.g., body weight) or binary (e.g., case control status). Since our goal is to provide a simple geometric interpretation of coexpression network analysis, we define the **trait-based gene significance measure** by raising the correlation between the *i*th gene expression profile *x_i_* and the clinical trait *T* to a power *β*

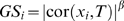
(4)Although any power *β* could be used in Equation 4, we use the same power as in Equation 2 to facilitate a simple geometric interpretation.

### Geometric Interpretation Using a Hypersphere

We find it convenient to express network quantities in terms of correlation coefficients since the correlation between two vectors can be interpreted as the cosine of the angle between them (measured in radians) if the vectors are scaled to have a mean of 0. Since the correlation is scale-invariant, i.e., cor(*ax_i_*+*b*, *cx_j_*+*d*) = cor(*x_i_*,*x_j_*), we can assume without loss of generality that the vectors *x_i_* have a mean 0 and are of the same length. In other words, they correspond to points on a hypersphere.

The network adjacency *a_ij_* is a monotonically decreasing function of the angle *θ_ij_* between the two scaled expression profiles if 0≤*θ_ij_*≤*π*/2. When the angle *θ_ij_* equals 0 or *π*/2, the adjacency equals 1 or 0, respectively. The network adjacency is a monotonically decreasing function of the length of the shortest path (geodesic) between the two points on the hypersphere. Soft thresholding methods (Equation 2) preserve the continuous nature of these distances. The higher the soft threshold *β*, the more weight is assigned to short geodesic distances compared to large distances.

Since the trait-based gene significance measure *GS_i_* = |cor(*x_i_*,*T*)|*^β^*, (Equation 4) is scale-invariant, the sample trait *T* can also be considered a point on the hypersphere. Analogous to the network adjacency, the smaller the geodesic distance between the *i*th gene expression profile and the trait *T*, the higher the gene significance of the *i*th gene. In other words, the smaller the angle between the sample trait and the expression profile, the more significant is the gene.

### A Motivational Example

**Figure 1:**
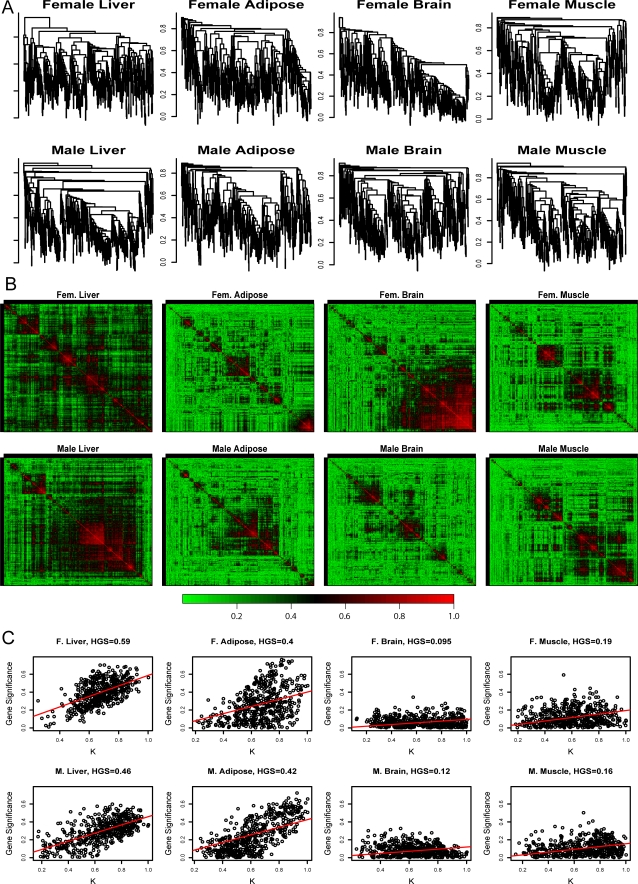
This motivational example explores the pairwise absolute correlations *a_ij_* = |cor(*x_i_*,*x_j_*)| among 498 genes in different mouse tissues. The biological significance of this network is described in [23],[26]. Each figure panel contains 8 subfigures for different genders and tissue types (liver, adipose, brain, muscle). (A) An average linkage hierarchical cluster tree of the genes. (B) The corresponding heat maps, which color-code the absolute pairwise correlations *a_ij_*: red and green in the heat map indicate high and low absolute correlation, respectively. The genes in the rows and columns of each heat map are sorted by the corresponding cluster tree. (C) The relationship between gene significance *GS* (*y*-axis) and connectivity (*x*-axis). The gene significance of the *i*th gene was defined as the absolute correlation between the *i*th gene expression profile and mouse body weight. The hub gene significance *HGS* (Equation 13) is defined as the slope of the red line, which results from a regression model without an intercept term.

As a motivational example, we study the pairwise correlations among 498 genes that had previously been found to form a sub-network related to mouse body weight. The microarray data measure the expression levels in multiple tissue samples (liver, adipose, brain, muscle) from male and female mice of an F2 intercross. Approximately 100 tissue samples are available for each gender/tissue combination. The biological significance of this subnetwork is described in [23],[26]. Here we focus on the mathematical and topological properties of the pairwise absolute correlations *a_ij_* = |cor(*x_i_*,*x_j_*)| between the genes. For each gender and tissue type Figure 1A depicts a hierarchical cluster tree of the genes. Figure 1B shows the corresponding heat maps, which color-code the absolute pairwise correlations *a_ij_*. As can be seen from the color bar underneath the heat maps, red and green in the heat map indicate high and low absolute correlation, respectively. The genes in the rows and columns of each heat map are sorted by the corresponding cluster tree.

It is visually obvious that the heat maps and the cluster trees of different gender/tissue combinations can look quite different. Network theory offers a wealth of intuitive concepts for describing the pairwise relationships among genes that are depicted in cluster trees and heat maps. To illustrate this point, we describe several such concepts in the following. By visual inspection of Figure 1B, genes appear to be more highly correlated in liver than in adipose (a lot of red versus green color in the corresponding heat maps). This property can be captured by the concept of network density (defined below). The density of the female liver network is 0.39 while it is only 0.23 for the female adipose network. Another example for the use of network concepts is to quantify the extent of cluster (module) structure. In this example, branches of a cluster tree (Figure 1A) correspond to modules in the corresponding network. The cluster structure is also reflected in the corresponding heat maps: modules correspond to large red squares along the diagonal. Network theory provides a concept for quantifying the extent of module structure in a network: the mean clustering coefficient (defined below). The female liver, male liver and female brain networks have high mean clustering coefficients (mean *ClusterCoef* = 0.42, 0.43, 0.41, respectively). In contrast, the female adipose, male adipose, and male brain networks have lower mean clustering coefficients (mean *ClusterCoef* = 0.27, 0.27, 0.25, respectively). Difference in module structure may reflect true biological differences or they may reflect noise (e.g. technical artifacts or tissue contaminations).

As another example for the use of network concepts, compare the cluster tree of the female brain network with that of the male brain network. The cluster tree of the female network appears to be comprised of a single large branch, i.e., a highly connected hub gene at the tip of the branch forms the center in this network. In contrast, the cluster tree corresponding to the male brain network appears to split into multiple smaller branches, i.e., no single gene forms the center. To measure whether a highly connected hub gene forms the center in a network, one can use the concept of centralization (defined below). The female brain and male brain networks have centralization 0.34 and 0.21, respectively.

These examples illustrate that graph theory contains a wealth of network concepts that can be used to describe microarray data. But we will argue that microarray data analysis techniques can also be used to derive network theoretic results. For example, network theorists have long studied the relationship between gene significance and connectivity. Several network articles have pointed out that highly connected hub nodes are central to the network architecture [17], [29]–[32] but hub genes may not always be biologically significant [33]. To define a sample trait based gene significance measure (Equation 4), we define the gene significance of gene *i* as the absolute correlation between the gene expression profile *x_i_* and body weight *T*, i.e., *GS_i_* = |cor(*x_i_*,*T*)|. Figure 1C shows the relationship between this gene significance measure and connectivity in the different gender/tissue type networks. We find a strong positive relationship between gene significance and connectivity in the female and the male mouse liver networks. The positive relationship between gene significance and connectivity suggests that both variables could be used to implicate genes related to body weight. For example, we used connectivity as a variable in a systems biologic gene screening method [26]. While most network theorists would agree that connectivity is an important variable for finding important genes in a network [17],[19], the statistical advantages of combining gene significance and connectivity are not clear. Below, we use the geometric interpretation of coexpression network analysis to argue that intramodular connectivity can be interpreted as a fuzzy measure of module membership. Thus, a systems biologic gene screening method that combines a gene significance measure with intramodular connectivity amounts to a pathway based gene screening method. Empirical evidence shows that the resulting systems biologic gene screening methods can lead to important biological insights [23]–[26]. Before combining gene significance and connectivity in a systems biologic gene screening approach, it is important to study their relationship. Toward this end, we propose a measure of hub gene significance *HGS* as slope of a regression line (through the origin) between gene significance and scaled connectivity. As can be seen from Figure 1C, the hub gene significance is high in liver and adipose tissues but it is low in brain and muscle tissues. Below, we use the geometric interpretation of coexpression networks to characterize coexpression networks that have high hub gene significance if the gene significance measure is based on a microarray sample trait *T*.

### Network Concepts

#### Abstract definition of network concepts

We define network concepts for (weighted) undirected networks that can be represented by a symmetric adjacency matrix *A* = [*a_ij_*], where 1≤*i*,*j*≤*n*. We assume that the pairwise adjacency (connection strength) *a_ij_* takes on values in the unit interval, i.e., 0≤*a_ij_*≤1. For notational convenience, we set the diagonal elements to 1. In the Methods section, we define a network concept *NCF*(*A*,*GS*) by evaluating a network concept function *NCF*(·,·) on the adjacency matrix *A* and/or a corresponding gene significance measure *GS*. This abstract definition will be useful in defining intramodular network concepts (e.g., Equation 17) and eigengene-based analogs of network concepts (e.g., Equation 30). In the following, we describe several network concepts including the connectivity, the maximum adjacency ratio, the density, and the centralization.

#### Connectivity and related concepts

The *connectivity* (also known as degree) of the *i*th gene is defined by
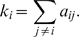
(5)In unweighted networks, the connectivity *k_i_* equals the number of genes that are directly linked to gene *i*. In weighted networks, the connectivity equals the sum of connection weights between gene *i* and the other genes.

The *maximum connectivity* is defined as
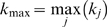
(6)The *scaled connectivity K_i_* of the *i*-th gene is defined by
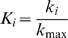
(7)By definition, 0≤*K_i_*≤1. Note that we distinguish the scaled from the unscaled connectivity by using an upper case “*K*” and a lower case “*k*”, respectively.

*Social Network Interpretation of the Connectivity:* For the aforementioned affection network (Equation 3), assume that the affection (adjacency) *a_ij_* equals 1 if two individuals strongly like each other; it equals 0.5 if they are neutral towards each other, and it equals 0 if they strongly dislike each other. Then the scaled connectivity *K_i_* is a measure of relative popularity: high values of *K_i_* indicate that the *i*th person is well liked by many others.

*Potential Uses of the Connectivity:* The connectivity is the most widely used concept for distinguishing the nodes of a network. As described in the motivational example and detailed below, intramodular connectivity can be used to define a systems biologic gene screening strategy that keeps track of module membership information [24].

#### Maximum adjacency ratio

For weighted networks, we define the *maximum adjacency ratio* of gene *i* as follows
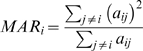
(8)which is defined if *k_i_* = *Σ_*j≠i*_*a_ij_*>0. One can easily verify that 0≤*a_ij_*≤1 implies 0≤*MAR_i_*≤1. Note that *MAR_i_* = 1 if all nonzero adjacencies take on their maximum value of 1, which justifies the name “maximum adjacency ratio.” By contrast, if all nonzero adjacencies take on a small (but constant) value *a_ij_* = *ε*, then *MAR_i_* = *ε* will be small.*

*Social Network Interpretation of the Maximum Adjacency Ratio: MAR_i_* = 1 suggests that the *i*th individual does not form neutral relationships; this individual either strongly likes or dislikes others. In contrast, *MAR_i_* = 0.5 suggests the *i*th individual forms less intense relationships with others.

*Potential Uses of the Maximum Adjacency Ratio:* Since *MAR_i_* = 1 for all genes in an unweighted network, the maximum adjacency ratio is only useful for weighted networks. The *MAR* can be used to determine whether a hub gene forms moderate relationships with a lot of genes or very strong relationships with relatively few genes. To illustrate this point, we show in the following simple example that the *MAR* can be used to distinguish nodes that have the same connectivity. Assume a network (labeled by *I*) for which the adjacency between node 1 and every other node equals *a*_1,*j*_^(I)^ = 1/(*n*−1). Then *k*_1_^(I)^ = (*n*−1)/(*n*−1) = 1 and *MAR*_1_^(I)^ = 1/(*n*−1). For a different network (labeled by II) where *a*_1,2_^(II)^ = 1 and *a*_1,*j*_^(II)^ = 0 for *j*≥3, the connectivity *k*_1_^(II)^ still equals 1 but *MAR*_1_^(II)^ = 1.

**Figure 2:**
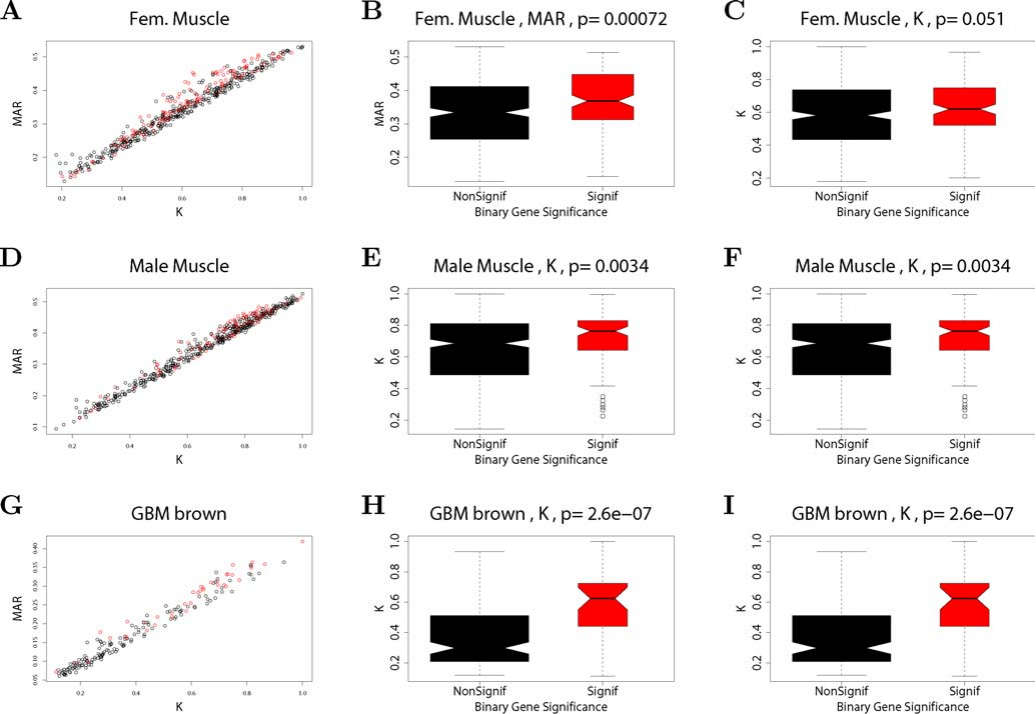
Relationships among maximum adjacency ratio, scaled connectivity, and gene significance. (A) The relationship between *MAR_i_* (y-axis) and scaled connectivity *K_i_* using the female mouse muscle tissue network described in the motivational example. The genes are colored red or black depending on whether they are significantly (*p*-value<0.05) related to mouse body weight. (B) Boxplots and a Kruskal-Wallis test *p*-value (*p* = 0.00072) for studying whether *MAR_i_* differs between significant (red) and non-significant (black) genes. (C) The analogous boxplots and *p*-value for the scaled connectivity *K_i_*. In this female muscle tissue application, *MAR_i_* is more significantly (*p* = 0.00072) related to *GS_i_* than is *K_i_* (*p* = 0.051). (D,E,F) The analogous relationships for male muscle. Here, the *MAR_i_* is more significantly (*p* = 0.00014) related to *GS_i_* than is *K_i_* (*p* = 0.0034). (G,H,I) The analogous relationships for the brown module of the brain cancer application. Here, the *MAR_i_* is slightly more significantly (*p* = 1.6E-8) related to *GS_i_* than is *K_i_* (*p* = 2.6E-7). As a caveat, we mention that in other applications (e.g., the yeast network), we have found that *K_i_* is more significantly related to *GS_i_* than *MAR_i_*.

In weighted coexpression networks, we find empirically that *MAR_i_* is often highly correlated with the connectivity *K_i_* (see also Equation 36). As we demonstrate in Figure 2, the *MAR_i_* is sometimes (but not always) superior to *K_i_* when it comes to identifying biologically important intramodular hub genes. As aside, we mention that a directed network analog of *MAR_i_* has been used in the analysis of metabolic fluxes [34].

#### Network density

The *network density* (also known as line density [35]) is defined as the mean off-diagonal adjacency and is closely related to the mean connectivity.
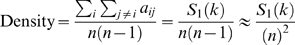
(9)where *k* = (*k*_1_,…,*k_n_*) denotes the vector of connectivities and the function vector *v* is defined by *S_p_*(*v*) = *Σ_*i*_*v_i_^p^*.*

*Social Network Interpretation of the Density:* The density measures the overall affection among individuals. A density close to 1 indicates that all individuals strongly like each other while a density of 0.5 suggests the presence of more ambiguous relationships.

*Potential Uses of the Density:* The density of genes in a subnetwork (e.g., a pathway) can be used to measure whether this sub-network is tight or cohesive. In our motivational mouse tissue example, we find that a network of genes has high density in liver tissue but low density in adipose tissue. The goal of many module detection methods is to find clusters of genes with high density.

#### Network centralization

The *network centralization* (also known as degree centralization [36]) is given by
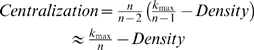
(10)The centralization is 1 for a network with star topology; by contrast, it is 0 for a network where each node has the same connectivity. A regular grid network such as a square has centralization 0.

*Social Network Interpretation of the Centralization:* The centralization of the affection network is close to 1, if one individual has loving relationships with all others who in turn strongly dislike each other. In contrast, a centralization of 0 indicates that all individuals are equally popular.

*Potential Uses of the Centralization:* While the centralization is a widely used measure in social network studies, it has only rarely been used to describe structural differences of metabolic networks [37]. As described in our motivational example, the centralization can be used to describe properties of cluster trees, see also [8].

#### Network heterogeneity

The *network heterogeneity* measure is based on the variance of the connectivity. Authors differ on how to scale the variance [35]. We define it as the coefficient of variation of the connectivity distribution, i.e.
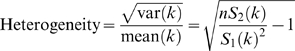
(11)This heterogeneity measure is invariant with respect to multiplying the connectivity by a scalar.

*Social Network Interpretation of the Heterogeneity:* The heterogeneity can be used to measure the variation of popularity (connectivity) across the individuals.

*Potential Uses of the Heterogeneity:* Describing the reasons for and the meaning of the heterogeneity of complex networks has been the focus of considerable research in recent years [29],[38]. Many complex networks have been found to exhibit an approximate scale-free topology, which implies that these networks are very heterogeneous [3].

#### Clustering coefficient

The *clustering coefficient* of gene *i* is a density measure of local connections, or “cliquishness” [9]. Specifically,
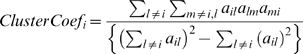
(12)In unweighted networks, *ClusterCoef_i_* equals 1 if and only if all neighbors of *i* are also linked to each other. For weighted networks, 0≤*a_ij_*≤1 implies that 0≤*ClusterCoef_ij_*≤1 [19].

*Social Network Interpretation of the Clustering Coefficient:* The higher the clustering coefficient of an individual, the higher is the affection among his friends. The clustering coefficient is zero if all of his friends strongly dislike each other.

*Potential Uses of the Clustering Coefficient:* As described in our motivational example, the mean clustering coefficient has been used to measure the extent of module structure present in a network. The relationship between the clustering coefficient and connectivity has been used to describe structural (hierarchical) properties of networks [1].

#### Hub gene significance

**Figure 3:**
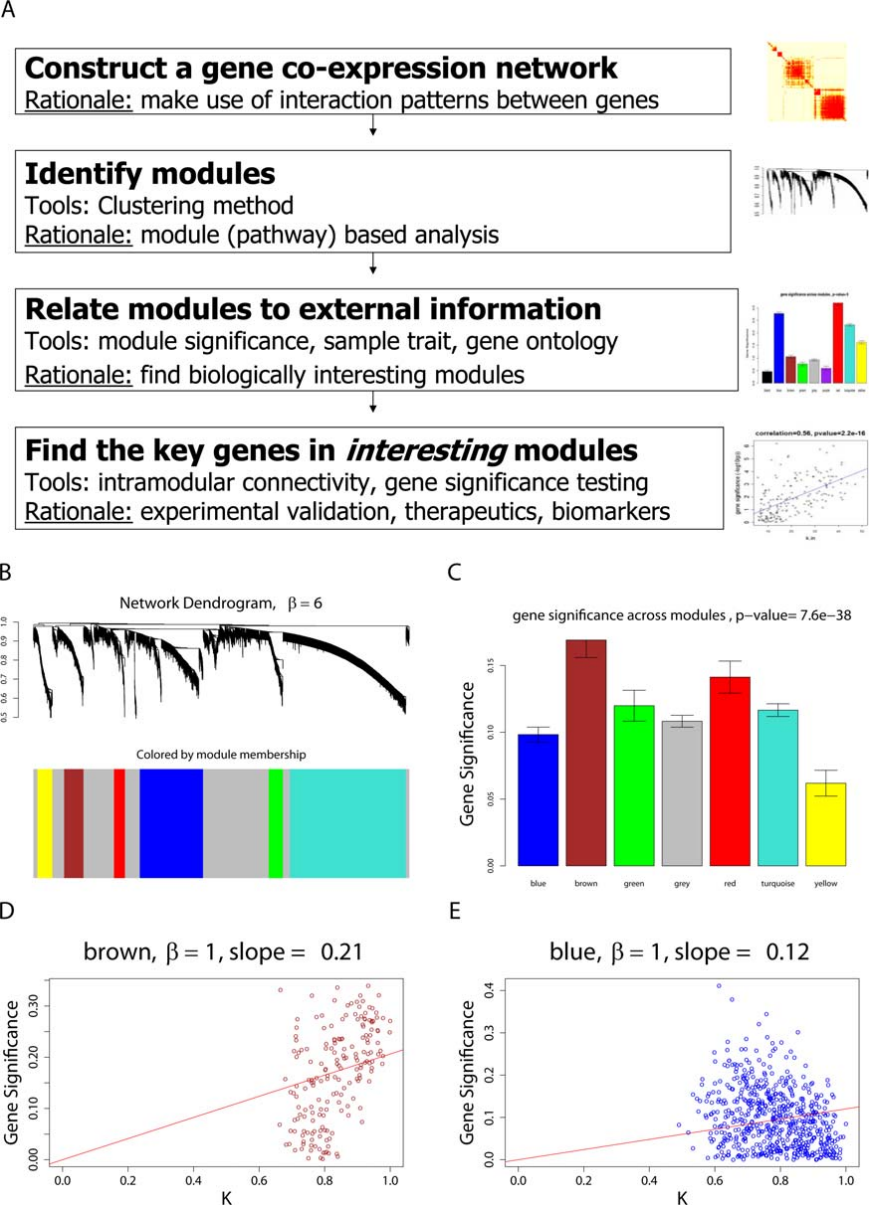
Overview and an example application of gene coexpression network analysis. (A) Outline of an analysis flow chart. Gene coexpression network analysis aims to identify pathways (modules) and their key drivers (e.g., intramodular hub genes). (B) The hierarchical cluster tree of genes in the brain cancer network. Modules correspond to branches of the tree. The branches and module genes are assigned a color as can be seen from the color-bands underneath the tree. Grey denotes genes outside of proper modules. A functional enrichment analysis of these modules can be found in Horvath et al. (2006). (C) The module significance (average gene significance) of the modules. The underlying gene significance is defined with respect to the patient survival time (Equation 4). (D,E) Scatter plots of gene significance *GS* (*y*-axis) versus scaled connectivity *K* (*x*-axis) in the brown and blue module, respectively. The hub gene significance (Equation 13) is defined as the slope of the red line, which results from a regression model without an intercept term.

To measure the association between connectivity and gene significance, we propose the following measure of *hub gene significance*:
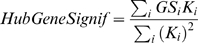
(13)When *GS_i_* is proportional to the scaled connectivity (*GS_i_* = *cK_i_*), the hub gene significance equals the constant of proportionality: *HubGeneSignif* = *c*. The hub gene significance equals the slope of the regression line between *GS_i_* and *K_i_* if the intercept term is set to 0 (Figure 3D and 3E).

*Social Network Interpretation of the Hub Gene Significance:* Assume that the node significance measures the grade point average of the *i*th individual. Then the hub node significance can be used to assess whether there is a relationship between popularity (connectivity) and grade point average.

*Potential Uses of the Hub Gene Significance:* Several studies have shown that the relationship between connectivity and gene significance (i.e., the hub gene significance) carries important biological information. For example, in the analysis of yeast networks, highly connected hub genes were found to be essential for yeast survival and there is evidence that hub genes are preserved across species [17], [25], [29]–[32]. A detailed analysis shows that the positive relationship between connectivity and knockout essentiality cannot always be observed [33], i.e., the hub gene significance can be close to 0.

#### Network significance measure

We define the *network significance measure* as the average gene significance of the genes:
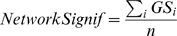
(14)


*Social Network Interpretation of the Network Significance:* The network significance simply measures the average grade point average among the individuals.

*Potential Uses of the Network Significance:* We refer to the network significance of a module network as “module significance.” The module significance measure can be used to address a major goal of gene network analysis: the identification of biologically significant subnetworks or pathways.

#### Centroid significance and centroid conformity

We define the *centroid significance* as the gene significance of a suitably chosen representative node (centroid) in the network.
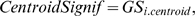
(15)where *i.centroid* denotes the index associated with the centroid. A centroid can be defined in many different ways, e.g., based on connectivity or other centrality measures. In our applications, we define the centroid as the most highly connected gene in the network. If multiple genes attain the maximum connectivity, we define the centroid significance by their average gene significance.

We define the *centroid conformity* of the *i*th gene as the adjacency between the centroid and the *i*th gene
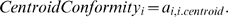
(16)If multiple genes attain the maximum connectivity, we define the centroid conformity as their average adjacency with the *i*th gene.

*Social Network Interpretation of the Centroid Conformity:* In our affection network, we choose the most popular individual as centroid; then his or her grade point average is the centroid significance. The centroid conformity of the *i*th individual equals his or her affection (connection strength) with the most popular individual.

*Potential Uses of the Centroid Conformity:* Below, we will characterize coexpression networks for which the adjacency *a_ij_* can be approximated by a product of the centroid conformities: *a_ij_*≈*CentroidConformity_i_ CentroidConformity_j_*. We will use this insight to derive relationships among seemingly disparate network concepts. For example, the mean clustering coefficient (Equation 12), the density (Equation 9), and the heterogeneity (Equation 11) measure different network properties but we show that they satisfy a simple relationship (Equation 31) in coexpression modules. Further, we will use the centroid significance to derive a simple relationship (Equation 37) between module significance (Equation 14) and hub gene significance (Equation 13).

### Overview of Weighted Gene Coexpression Network Analysis

One of the many biological applications of gene coexpression networks is the identification of pathways (modules) and centrally located genes (referred to as module centroids). In our applications, we define highly connected intramodular hub genes as module centroids. Weighted gene coexpression network analysis (WGCNA, [19],[24]) can be considered a step-wise microarray data reduction technique, which starts from the level of thousands of genes, identifies clinically interesting gene modules, and finally represents the modules by their centroids. The module centric analysis alleviates the multiple testing problem inherent in microarray data analysis. Instead of relating thousands of genes to a sample trait, it focuses on the relationship between a few (usually less than 10) modules and the sample trait.

An outline of WGCNA is presented in Figure 3A. The module definition does not make use of *a priori* defined gene sets. Instead, modules are constructed from the expression data by using a tight clustering procedure. Although it is advisable to relate the resulting modules to gene ontology information to assess their biological plausibility, it is not required. Because the modules may correspond to biological pathways, focusing the analysis on modules (and corresponding centroids) amounts to a biologically motivated data reduction method. Intramodular hub genes are centrally located in the module and thus lend themselves as candidates for biomarkers. Examples of biological studies that show the importance of intramodular hub genes can be found reported in [23]–[25],[33],[39]. Because the expression profiles of intramodular hub genes are highly correlated (in our data, *r*>0.90), typically dozens of candidates result. Although these candidates are statistically equivalent, they may differ in terms of biological plausibility or clinical utility.

### Network Modules

Roughly speaking, we define network modules as groups of highly interconnected genes. As detailed in Text S1, Text S2, Text S3, and in our online R tutorials, we use a hierarchical clustering procedure to identify modules (clusters) as branches of the resulting cluster tree. A common but inflexible branch cutting method uses a constant height cutoff value. Alternatively, dynamic branch cutting adaptively chooses cutting values depending on the shape of the branch [40]. Each module is assigned a unique color label (Figure 3B). Our branch cutting algorithm only assigns module colors to branches whose size exceeds a user-specified threshold parameter. In practice, it is advisable to vary the minimum module size and other branch cutting parameters to determine how the results are affected by different parameter choices. An iterative approach for choosing the parameters could be defined by optimizing the module significance. This module detection approach has led to biologically meaningful modules in several applications [1], [8], [23]–[25], [33], [39]–[43] but our theoretical results transcend this particular module detection method. Any module detection method that results in clusters of highly correlated gene expressions could be used.

### Intramodular Network Concepts

In the following, we assume that a module detection method (e.g., a clustering procedure) has found *Q* modules. We denote the adjacency matrix of the genes inside the *q*th module by *A* ^(*q*)^. Thus, *A* ^(*q*)^ represents a subnetwork comprised of the genes in the *q*th module. Analogously, we define *GS* ^(*q*)^ as the gene significance measure restricted to the module genes. Denote by *n* ^(*q*)^ the number of genes inside the *q*th module. Throughout the manuscript, we use the superscript (*q*) to denote quantities associated with the *q*th module. But for notational convenience, we sometimes omit (*q*) when the context is clear.

We define an intramodular network concept *NCF*(*A* ^(*q*)^,*GS* ^(*q*)^) by evaluating a network concept function *NCF*(·,·) on the adjacency matrix *A* ^(*q*)^ and/or a corresponding gene significance measure *GS* ^(*q*)^.

For example, the intramodular connectivity is defined by
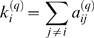
(17)where the *j* indexes the genes in the *q*th module. Intramodular connectivity has been found to be an important complementary gene screening variable for finding biologically important genes [24],[25],[39].

We refer to the network significance (Equation 14) of a module network simply as the **module significance measure**, i.e., the module significance is the average gene significance of the module genes:
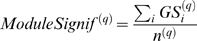
(18)


### Data Reduction Methods for Microarray Data

The high dimensionality of gene expression data has inspired two broad categories of data reduction techniques. The first category, often used by network theorists, is to reduce the gene coexpression networks into modules. Each module can be represented by a centroid, e.g., an intramodular hub gene. The second category, often used by microarray data analysts, reduces the gene expression data to a small number of components that capture the essential behavior of the expression profiles [27], [44]–[51]. One of our goals is to understand how the two categories of data reduction methods relate to each other. Here we use the singular value decomposition [44],[45],[48] since this will allow us to define a simple measure of factorizability (Equation 24).

#### Singular value decomposition

For the *q*th module, denote by *X* ^(*q*)^ the *n* ^(*q*)^×*m* matrix of *n* ^(*q*)^ gene expression profiles across *m* microarrays:
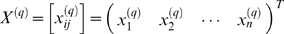
(19)where *x_i_* denotes the gene expression vector of the *i*th gene.

The singular value decomposition (SVD) of *X* ^(*q*)^ is given by *X* ^(*q*)^ = *U* ^(*q*)^ *D* ^(*q*)^(*V* ^(*q*)^)*^T^*, where *U* ^(*q*)^ is an *n* ^(*q*)^×*m* matrix with orthonormal columns, *V* ^(*q*)^ is an *m*×*m* orthogonal matrix, and *D* ^(*q*)^ is an *m*×*m* diagonal matrix of the singular values {|*d_l_* ^(*q*)^|}. Specifically, *V* ^(*q*)^ and *D* ^(*q*)^ are given by
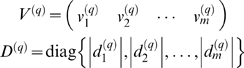
(20)


The singular value decomposition of *X* ^(*q*)^ is closely related to the principal component analysis of the correlation matrix *COR* = [cor(*x_i_* ^(*q*)^,*x_j_* ^(*q*)^)] whose entries correspond to the pairwise correlations between the rows (genes) of *X* ^(*q*)^. For example, the eigenvalues of the correlation matrix *COR* are squares of corresponding singular values |*d_l_* ^(*q*)^|.

We assume that the singular values |*d_l_* ^(*q*)^| are arranged in decreasing order. Adapting terminology from [44], we refer to the first column of *V* ^(*q*)^ as the **Module Eigengene**:
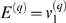
(21)


**Figure 4:**
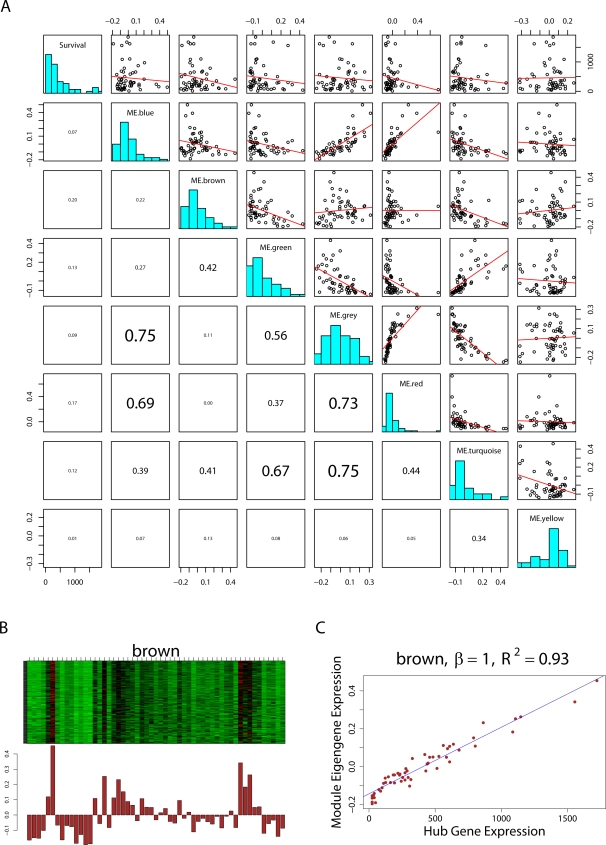
Module eigengenes in the brain cancer gene coexpression network. (A) The pairwise scatter plots among the module eigengenes *E* ^(*q*)^ of different modules and cancer survival time *T*. Each dot represents a microarray sample. ME.blue denotes the module eigengene *E* ^(blue)^ of the blue module. Numbers below the diagonal are the absolute values of the corresponding correlations. Note that the module eigengenes of different modules can be highly correlated. The brown module eigengene has the highest absolute correlation (*r* = 0.20) with survival time. Frequency plots (histograms) of the variables are plotted along the diagonal. (B) Upper panel: heat map plot of the brown module gene expression profiles (rows) across the microarray samples (columns). Red corresponds to high- and green to low- expression values. Since the genes of a module are highly correlated, one observes vertical bands. (B) Lower panel: the values of the components of the module eigengene (*y*-axis) versus microarray sample number (*x*-axis). Note that vertical bands of red (green) in the upper panel correspond to high (low) values of the eigengene in the lower panel. (C) The expression profile of the module eigengene (*y*-axis) is highly correlated with that of the most highly connected hub gene (*x*-axis). A linear regression line has been added.

For brevity, we sometimes drop the superscript (*q*) and simply refer to *E* as the eigengene. The module eigengene can be used to summarize and represent the expression profiles of the module genes, see Figure 4B. The proportion of variance explained by the module eigengene *E* ^(*q*)^ is defined as
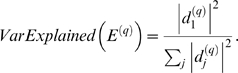
(22)


### High Level View of Gene Coexpression Networks and Eigengene Networks

The module eigengenes of different modules can be highly correlated (Figure 4A). Detecting a high correlation between module eigengenes may either be of biological interest (suggesting interactions between pathways) or it may be a methodological artifact (suggesting poorly defined modules that should be merged). The correlations between two eigengenes can be used to define eigengene coexpression networks [52], e.g., a weighted eigengene coexpression network can be defined as follows
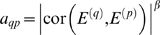
(23)where *E* ^(*q*)^ and *E* ^(*p*)^ represent the eigengenes of two distinct modules. Apart from correlating the module eigengenes of different modules to each other, one can relate the module eigengenes to an external microarray sample trait *T* to identify trait related modules. Thus, eigengene network analysis can be viewed as a network reduction scheme that reduces a gene coexpression network involving thousands of genes to an orders of magnitude smaller metanetwork involving module representatives (one eigengene per module).

Unlike traditional microarray data reduction methods that impose orthogonality (e.g., principal component analysis) or independence (e.g., independent component analysis), gene coexpression network analysis can be considered a pathway-based data reduction method that allows dependencies between the modules. When focusing on the use of module eigengenes, network analysis can be considered a variant of oblique factor analysis.

### Low Level View of a Single Module and Factorizable Networks

While a high level view of modular gene coexpression networks can be viewed as a data reduction technique, many network analyses focus on the pairwise relationships of relatively few (hundreds) of correlated genes, i.e., genes that form a single module in a larger network. For example, the 498 genes of our motivational example were part of a body weight related module, which was found in a large gene coexpression network based on the female mouse liver samples [23].

The low-level analysis of a single network module may help identify key genes that may be used as therapeutic targets or candidate biomarkers. An important question of low level analysis is to efficiently describe the connection strengths between interacting module genes. We have provided *empirical* evidence that many module adjacency matrices, i.e., networks comprised of genes of a single module, are approximately factorizable [8]. In such networks, the adjacency between module genes *i* and *j* can approximately be factored into gene specific contributions, i.e., *a_ij_* ^(*q*)^≈*CF_i_* ^(*q*)^ *CF_j_* ^(*q*)^ with *CF_i_* ^(*q*)^ defined as the conformity of gene *i*. Thus, the adjacency matrix of an approximately factorizable network can be approximated using the rank 1 matrix [*CF_i_* ^(*q*)^ *CF_j_* ^(*q*)^]. The conformity vector *CF* ^(*q*)^ can be estimated in several ways [8]; it is highly related to a single factor nonnegative matrix decomposition of *A* ^(*q*)^ [51] and it is highly related to the connectivity 
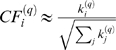
.

#### Characterizing approximately factorizable coexpression modules

An open theoretical research question is to characterize microarray data that lead to factorizable coexpression networks. Here we solve this problem for the case of modules in a gene coexpression network. Toward this end, we propose the following measure of **eigengene factorizability**:
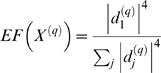
(24)Note that 0≤*EF*(*X* ^(*q*)^)≤1 and the close resemblance to the proportion of variance explained by the module eigengene (Equation 22). In the Methods section, we argue that *EF*(*X* ^(*q*)^)≈1 implies that the correlation matrix factors as follows
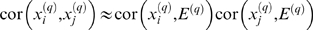
Further, we derive the following

#### Observation 1

*If the eigengene factorizability EF(X^(q)^) is close to 1, the adjacencies of the weighted coexpression module network A^(q)^ = |cor(X^(q)^)|^β^ and the trait-based gene significance measure GS_i_^(q)^ = |cor(x_i_^(q)^,T)|^β^ can be factored as follows*

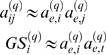
(25)
*where*

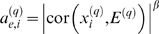
(26)
*is referred to as the* ***eigengene conformity*** *of the ith gene, and*

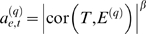
(27)
*is referred to as the qth module* ***eigengene significance*** *with respect to T, also denoted as EigengeneSignif^(q)^.*

As described in Table 1, the eigengene significance and the eigengene conformity are the eigengene-based counterparts of the centroid significance (Equation 15) and centroid conformity (Equation 16), respectively.

**Table 1:** Dictionary for translating between general network terms and their eigengene-based counterparts.

Term	General network	Gene expression
	Adjacency matrix *A* ^(*q*)^ = [*a_ij_*]	Microarray data *X* ^(*q*)^
Decomposition	Factor analysis of *A*	Singular value decomposition of *X* = *UDV^T^*
Centroid	Intramodular hub gene	Module eigengene *E*
Conformity(i)	*CF_i_* defined as 1st factor of *A*	*a_e_*_,*i*_ = |cor(*x_i_*,*E*)|*^β^*
Approximately factorizable means	*a_ij_*≈*CF_i_CF_j_*	*x_i_*≈*u*_1_(*i*)|*d*_1_|*E*
Factorizability measure	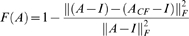	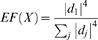
CentroidSignif(i)	*GS_i,centroid_*	*a_e_*_,*t*_ = |cor(*E*,*T*)|*^β^*
CentroidConformity(i)	*a_i.centroid,i_*	*a_e_*_,*i*_ = |cor(*E*,*x_i_*)|*^β^*
Weighted gene coexpression network and its eigengene-based approximation if *EF*(*X* ^(*q*)^)≈1		
	Coexpression network	Eigengene-based counterpart
Network	*A* = |cor(*X*)|*^β^*	*A_E_* = *a_e_a_e_^T^*
Gene significance(i)	*GS_i_* = |cor(*x_i_*,*T*)|*^β^*	*GS_E_*_,*i*_ = *a_e,i_a_e,t_*
Connectivity(*i*)	*k_i_* = *Σ_j≠i_a_ij_*	*k_E,i_* = *a_e_*_,*i*_ *Σ_j_a_e_*_,*j*_
Network concepts based on a network concept function *NCF*(·,·) if *EF*(*X* ^(*q*)^)≈1 and max*_j_*(*a_e_*_,*j*_)≈1		
	Intramodular	Eigengene-based
Concept	*NCF(A,GS)*	*NCF(A_E_,GS_E_)*
Scaled connectivity(*i*)	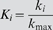	*K_E,i_*≈*a_e_*_,*i*_
Density	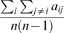	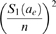
Centralization	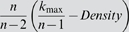	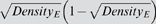
Heterogeneity	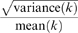	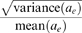
Clustering Coefficient(*i*)	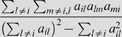	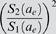
Max. adjacency ratio(*i*)	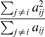	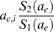
Hub gene significance	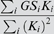	*a_e_*_,*t*_
Module significance	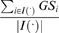	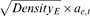

Here we omit the reference to the *q*th module.

The eigengene-based approximations on the right hand side of Equation 25 motivate us to define the eigengene-based adjacency matrix *A_E_* ^(*q*)^ and gene significance measure *GS_E_* ^(*q*)^ as follows:
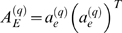
(28)

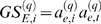
(29)For our coexpression modules, we find empirically that the eigengene factorizability is close to 1 (see Table 2, Text S1, Text S2, and Text S3).

**Table 2:** Values of network concepts in weighted gene coexpression module networks (brain cancer data).

Module	Blue	Brown	Green	Grey	Red	Turquoise	Yellow
Module size (*n* ^(*q*)^)	606	185	136	1313	105	1112	143
Eigengene factorizability (*EF*(*X* ^(*q*)^))	0.97	0.99	0.99	0.66	0.98	0.98	0.99
*VarExplained(E^(q)^)*	0.59	0.66	0.70	0.28	0.68	0.57	0.71
Max. conformity max(*a_e_*_,*i*_)	0.97	0.97	0.98	0.91	0.95	0.98	0.98
*Density*	0.58	0.65	0.69	0.29	0.67	0.55	0.70
*Density_E_*	0.58	0.65	0.70	0.23	0.68	0.55	0.71
*Centralization*	0.16	0.13	0.12	0.15	0.11	0.17	0.12
*Centralization_E_*	0.16	0.13	0.12	0.21	0.11	0.18	0.12
*Heterogeneity*	0.14	0.10	0.11	0.17	0.091	0.17	0.11
*Heterogeneity_E_*	0.14	0.10	0.11	0.44	0.091	0.17	0.11
*Mean(ClusterCoef)*	0.60	0.66	0.71	0.32	0.68	0.59	0.72
*ClusterCoef_E_*	0.60	0.66	0.71	0.33	0.68	0.59	0.72
*ModuleSignif*	0.088	0.12	0.21	0.11	0.16	0.14	0.065
*ModuleSignif_E_*	0.018	0.093	0.21	0.008	0.16	0.13	0.039
*HubGeneSignif*	0.11	0.14	0.25	0.15	0.18	0.19	0.074
*HubGeneSignif_E_*	0.023	0.11	0.25	0.014	0.18	0.17	0.045
*EigengeneSignif = a_e,i_^(q)^*	0.024	0.12	0.25	0.016	0.19	0.18	0.046

Here we report the results for soft thresholding with *β* = 1 (Equation 2). The results for higher powers *β* and for unweighted networks can be found in Text S1. Grey colors genes outside the 6 properly defined modules. The table shows that network concepts in the proper modules are close to their eigengene based analogs.

Abstractly speaking, Observation 1 allows us to characterize coexpression networks for which the adjacency *a_ij_* can be approximated by a product of the centroid conformities (Equation 16): *a_ij_*≈*CentroidConformity_i_ CentroidConformity_j_*.

#### Geometric interpretation of factorizability

**Figure 5:**
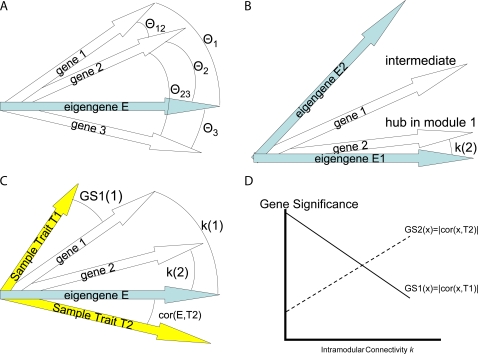
Using vectors to illustrate results in gene coexpression network analysis. (A) A geometric interpretation of factorizability if the gene expression profiles and the module eigengene lie in a Euclidean plane. Then the angle *θ*_12_ between gene expressions profiles 1 and 2 can be expressed in terms of angles with the module eigengene, i.e., *θ*_12_ = *θ*_1_−*θ*_2_. Similarly, *θ*_23_ = *θ*_2_+*θ*_3_. Under the assumptions stated in the text, we find *θ_ij_*≈|*θ_i_*±*θ_j_*|. Using a trigonometric formula (Equation 51) this implies that the correlation matrix is approximately factorizable. (B) Illustrating why intramodular hub genes cannot be “intermediate” genes between two distinct coexpression modules. The large angle between module eigengenes *E1* and *E2* reflects that the corresponding modules are distinct. Since intermediate gene 1 does not have a small angle with either eigengene, it is not an intramodular hub gene. By contrast, intramodular hub gene 2 has a small angle with eigengene *E1* but is not close to module eigengene *E2*. (C,D) Illustrating that the hub gene significance of a module depends on the relationship between the module eigengene and the underlying microarray sample trait (Equation 34). For sample traits *T2* and *T1* the hub gene significance (and corresponding eigengene significance cor(*E*,*T*)) are high and low, respectively. The geometry of (C) implies relationships between the connectivity *k* of a gene (determined by its angle with the eigengene E) and gene significance measure *GS1* (its angle with trait *T1*) and *GS2* (its angle with trait *T2*). As shown in (D), the gene significance measure *GS2* increases with *k* since the small angle between *E* and *T2* implies that genes with high *k* (small angle with *E*) also have a small angle with *T2*. In contrast, high connectivity *k* implies a large angle with *T1* and thus *GS1* decreases as a function of *k*.

In the Methods section, we argue that *EF*(*X* ^(*q*)^)≈1 if the module gene expressions *x_i_* ^(*q*)^ are approximately orthogonal to the right singular vectors *v_l_* ^(*q*)^ for *l*≥2, i.e., if on average the gene expression profiles point in the direction of the module eigengene *v*_1_^(*q*)^ = *E* ^(*q*)^. A rough geometric intuition of *a_ij_* ^(*q*)^≈*a_e,j_* ^(*q*)^ *a_e,j_* ^(*q*)^ (Equation 25) is presented in Figure 5A. The angle between the module eigengene *E* ^(*q*)^ and the *i*th gene expression profile is denoted by *θ_i_*. The angle between gene expression profiles *i* and *j* is denoted by *θ_ij_*. In the Methods section, we show that *θ_ij_*≈|*θ_i_*±*θ_j_*| and sin(*θ_i_*) sin(*θ_j_*)≈0 imply approximate factorizability of the correlation matrix.

### Eigengene-Based Analogs of Network Concepts

Here we define **eigengene-based network concepts** as a step towards a geometric interpretation of network concepts. Analogous to the case of intramodular network concepts, we define eigengene-based network concepts by evaluating the network concept function *NCF*(*A_E_* ^(*q*)^,*GS_E_* ^(*q*)^) on the eigengene-based adjacency matrix *A_E_* ^(*q*)^ (Equation 28) and the eigengene-based gene significance measure *GS_E_* ^(*q*)^ (Equation 29). One can easily derive the following formulas for eigengene-based network concepts:
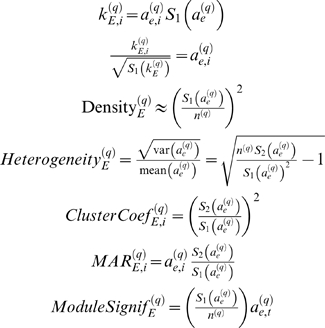
(30)where 
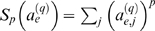
. Under the assumptions of Observation 1, we find that *A* ^(*q*)^≈*A_E_* ^(*q*)^ and *GS_i_*≈*GS_E,i_*. For a continuous network concept function *NCF*(·,·) this implies *NCF*(*A* ^(*q*)^,*GS*)≈*NCF*(*A_E_* ^(*q*)^,*GS_E_*). We summarize this observation as follows

#### Observation 2

*If A^(q)^ = |cor(X^(q)^)|^β^ and the eigengene factorizability EF(X^(q)^) is close to 1, the network concepts can be approximated by their eigengene-based analogs.*

**Figure 6:**
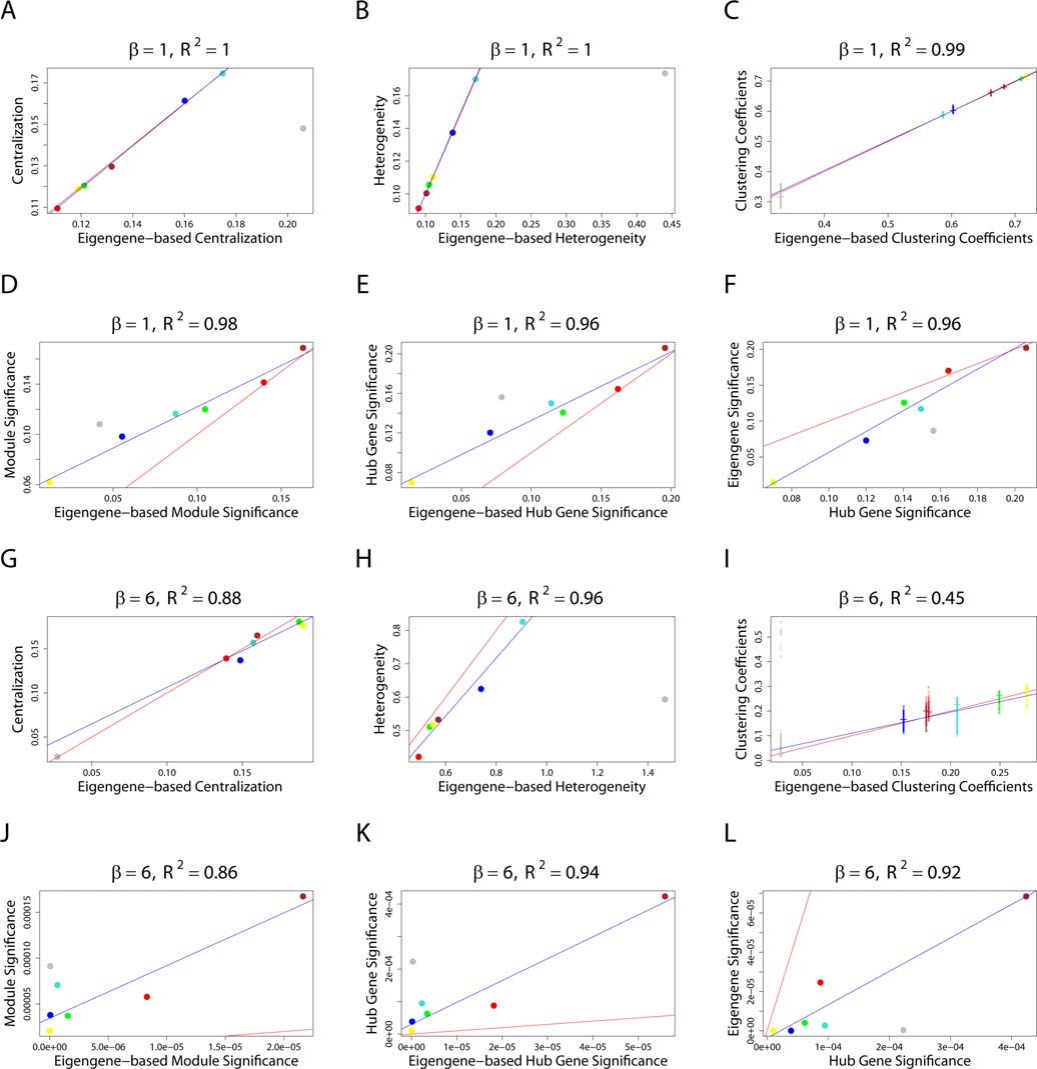
Illustrating Observation 2 regarding the relationship between a network concept (*y*-axis) and its eigengene-based analog (*x*-axis) in the brain cancer data. Each point corresponds to a module. (A–F) Corresponding to a weighted network constructed with a soft threshold (Equation 2) of *β* = 1. (G–L) Analogous plots for *β* = 6. (A,G) Centralization (*y*-axis) versus eigengene-based Centralization_E_ (*x*-axis). The following are analogous plots for (B,H): heterogeneity; (C,I) clustering coefficient; (D,J) module significance; and (E,K) hub gene significance. (F,L) Illustrating Equation 13 regarding the relationship between eigengene significance and hub gene significance. The blue line is the regression line through the points representing proper modules (i.e., the grey, nonmodule genes are left out). While the red reference line (slope 1, intercept 0) does not always fit well, we observe high squared correlations *R* ^2^ between network concepts and their analogs. Since the grey point corresponds to the genes outside properly defined modules, we did not include it in calculations.

This observation is illustrated in Figure 6.

#### Using the eigengene-based heterogeneity to study the effect of soft thresholding

It can be advantageous to replace network concepts by their eigengene-based analogs when studying theoretical properties. To illustrate this point, we briefly describe the effect of soft thresholding *a_ij_* = *s_ij_^β^* (Equation 2) on the network heterogeneity. Using extensive simulation studies reported on our webpage, we found that for the vast majority of networks, the heterogeneity increases with the soft threshold *β*. Thus, for most coexpression networks, increasing *β* makes it easier to discern highly connected genes from less connected genes. However, one can construct networks for which increasing *β* leads to a lower heterogeneity. The situation is much simpler for the eigengene-based heterogeneity *Heterogeneity_E_* ^(*q*)^ (Equation 30). In the Methods section, we prove that the eigengene-based heterogeneity is a monotonically increasing function of the soft threshold *β*. Thus, the heterogeneity will be an increasing function of *β* if it can be approximated by its eigengene based analog (Observation 2).

#### Relationships among eigengene-based network concepts

A major theoretical advantage of eigengene-based network concepts is that they reveal simple relationships amongst each other. For example, it is straightforward to derive
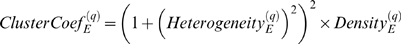
(31)


To arrive at particular simple relationships among network concepts, we make use of the following terminology. We denote the **maximum eigengene conformity** as *a_e_*_,max_^(*q*)^ = max*_j_*(*a_e,j_* ^(*q*)^), where *a_e,j_* ^(*q*)^ = |cor(*x_j_* ^(*q*)^,*E* ^(*q*)^)|*^β^* (Equation 26). In most modules, we find genes that have very high correlations (*r*≈*0.99*) with the module eigengene. For a low power *β*, this implies that the maximum eigengene conformity is approximately equal to 1:
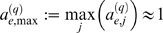
(32)


We refer to Equation 32 as the **maximum conformity assumption**. With the results in the Methods section, one can show that the maximum conformity assumption implies the following

#### Observation 3

*If A^(q)^ = |cor(X^(q)^)|^β^, EF(X^(q)^)≈1 and the maximum conformity assumption applies, intramodular network concepts satisfy the following relationships*

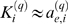
(33)

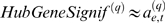
(34)

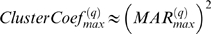
(35)

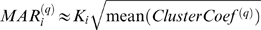
(36)

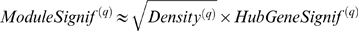
(37)

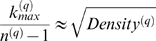
(38)

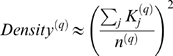
(39)

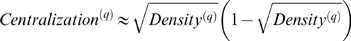
(40)
*where mean(ClusterCoef^(q)^) denotes the mean clustering coefficient, ClusterCoef_max_^(q)^ = max_j_(ClusterCoef_j_^(q)^) and MAR_max_^(q)^ = max_j_(MAR_j_^(q)^).*

In practice, we find that the maximum conformity assumption holds well for low values of *β*. Below, we study the robustness of our results with respect to higher powers and alternative network construction methods.

#### Geometric interpretation of network concepts

Observations 2 and 3 allow us to provide a geometric interpretation of intramodular network concepts.

The relationship between the scaled intramodular connectivity *K_i_* ^(*q*)^ and its eigengene based analog *a_e,i_* ^(*q*)^ = |cor(*x_i_* ^(*q*)^,*E* ^(*q*)^)|*^β^* (Equation 33) facilitates a geometric interpretation of the intramodular connectivity: the smaller the angle *θ_i_* between the *i*th gene expression profile and the module eigengene, the larger is |cos(*θ_i_*)|*^β^* = *a_e,i_* ^(*q*)^, i.e., the larger is the scaled intramodular connectivity. Since the module eigengene summarizes the overall behavior of the module, *a_e,i_* ^(*q*)^ measures how well gene *i* conforms to the overall module. Thus, a tongue-in-cheek social network interpretation of Equation 33 is that group-conforming behavior leads to high popularity.

We provide two geometric interpretations of the density. The first makes use of the relationship *a_ij_* ^(*q*)^ = |cos(*θ_ij_*)|*^β^* where *θ_ij_* denotes the angle between gene expression profiles *i* and *j*. By definition (Equation 9), the smaller the pairwise angles *θ_ij_* between the gene expression profiles, the higher is the module density. Equation 39 provides another interpretation: the smaller the angles *θ_i_* between the module gene expression profiles and the module eigengene, the higher is the density. Thus, the density can be interpreted as a measure of average closeness between the gene expression profiles and the module eigengene. By definition, coexpression module networks have a relatively high density (see Table 2, Text S1, Text S2, and Text S3).

The eigengene-based heterogeneity equals the coefficient of variation of the *a_E_* ^(*q*)^, i.e., it is a measure of variability of the angles *θ_i_* between the gene expression profiles and the module eigengene. The heterogeneity equals 0 if the angles *θ_i_* are all equal.

The *i*th gene has high eigengene-based significance *GS_E,i_* ^(*q*)^ (Equation 29) if the eigengene has a small angle with the sample trait and *θ_i_* is small. Similarly, the geometric interpretation of the hub gene significance (Equation 13) is straightforward: the smaller the angle between the module eigengene and the sample trait, the higher is the hub gene significance (Equation 34).

We provide two geometric interpretations of the module significance (Equation 14). The first interpretation is based on the definition of the module significance as average gene significance; a module has high module significance if on average the angles between the module expression profiles and the sample trait tend to be small. The second interpretation of the module significance is based on Equation 37: a module has high significance if the module density is high and the angle between the module eigengene and the sample trait is small.

### What Can Microarray Data Analysts Learn from the Geometric Interpretation?

Here we illustrate how the geometric interpretation of gene coexpression networks can be used to derive results, which may be interesting to microarray data analysts.

#### Summarizing the expression profiles of a module

Multiple approaches are conceivable for summarizing the expression profiles of the genes inside a single module. One approach (popular with statisticians) applies a singular value decomposition to the expression data and summarizes the module with the module eigengene. Another approach (popular with network theorists) is to construct a module network and to use the most highly connected hub gene as centroid. Since Equation 33 implies that hub genes are highly correlated with the module eigengene, we find that the two seemingly different approaches will lead to very similar results in practice (Figure 4C).

#### Intramodular connectivity is a measure of module membership

Since module construction is computationally intensive, one often restricts the module detection analysis to a subset of the original genes on the microarray, e.g., the most varying and/or the most connected genes. To counter this loss of information, generalizing the intramodular connectivity to extramodular genes, i.e. genes outside the module, is an important problem. Our solution is motivated by the relationship between the intramodular connectivity and its eigengene based analog (Equation 33). Specifically, the *q*th module eigengene gives rise to an eigengene-based scaled intramodular connectivity measure
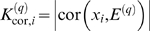
(41)


Under the assumptions of Observation 3, Equation 33 implies that *K_i_* ^(*q*)^≈|*K*_cor,*i*_^(*q*)^|*^β^* for the subset of genes that are in the *q*th module. The larger *K*_cor,*i*_^(*q*)^, the more similar is gene *i* is to the summary profile of the *q*th module. Thus, *K*_cor,*i*_^(*q*)^ can be used to measure module membership. A theoretical advantage of *K*_cor,*i*_^(*q*)^ over *K_i_* ^(*q*)^ is that its definition can be easily extended to expression profiles *x_i_* outside the *q*th module. Another advantage of *K*_cor,*i*_^(*q*)^ is that a simple correlation test *p*-value can be used to assess the statistical significance of the correlation between *x_i_* and *E* ^(*q*)^.

#### Fuzzy module annotation of genes

**Figure 7:**
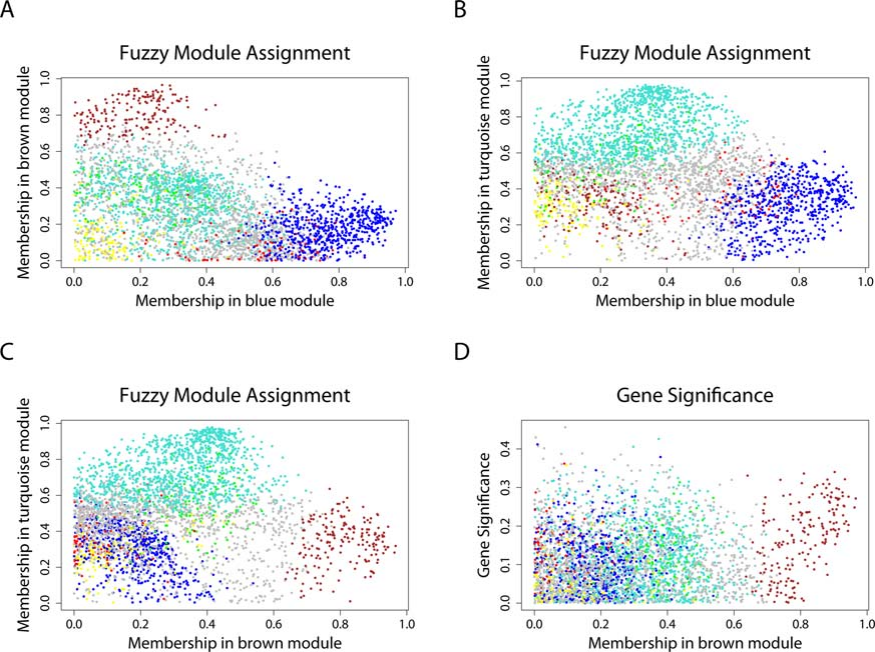
Fuzzy module annotation of genes in the brain cancer network. A natural choice for a fuzzy measure of module membership is the generalized scaled connectivity measure *K*_cor,*i*_^(*q*)^ = |cor(*x_i_*,*E* ^(*q*)^)| (Equation 41). (A) Scatterplot of the brown module membership measure (*y*-axis) versus that of the blue module (*x*-axis). Note that grey dots corresponding to genes outside of properly defined modules can be intermediate between module genes. (B) The corresponding plot for blue versus turquoise module membership. (C) Brown versus turquoise module membership. (D) The relationship between gene significance based on survival time (*y*-axis) and brown module membership (*x*-axis).

Module detection usually involves certain parameter choices. For some genes, it may be difficult to decide whether they belong to a particular module or whether they belong to more than one module. Instead of reporting a binary indicator of module membership, it can be advantageous to report a fuzzy measure of module membership, which takes on values in the unit interval [0,1]. A natural choice for a fuzzy measure of module membership is the eigengene-based scaled intramodular connectivity measure *K*_cor,*i*_^(*q*)^ (Equation 41). The fuzzy module membership measures *K*_cor,*i*_^(*q*)^ specify how close gene *i* is to modules *q* = 1,…,*Q*. It is straightforward to use these measures for finding genes that are close to two modules, i.e., intermediate genes. In Figure 7, we show the pairwise relationships among different *K*_cor,*i*_^(*q*)^ measures where the genes are colored by their original module assignment. Note that many of the nonmodule (grey) genes lie intermediate between the proper module genes.

### What Can Network Theorists Learn from the Geometric Interpretation?

In the following, we provide several examples that illustrate potential uses of the geometric interpretation.

#### Statistical significance of network concepts

While fundamental network concepts are defined as functions of the network adjacency matrix, their eigengene-based analogs are often simple monotonic functions of correlation coefficients. This insight can be used to attach significance levels (*p*-values) to several eigengene-based network concepts. For example, the eigengene-based hub gene significance is a monotonic function of the correlation between the eigengene and the sample trait (Equation 34). Thus, one can use a correlation test *p*-value [53] or a regression-based *p*-value for assessing the statistical significance between *E* ^(*q*)^ and the sample trait *T*. Analogously, one can attach a significance level to the fuzzy module membership measures *K*_cor,*i*_^(*q*)^ (Equation 41).

Since the gene coexpression network concepts are based on correlations between quantitative variables, one can use permutation test procedures to attach significance levels to network concepts. By randomly permuting the gene expression values of each gene, it is possible to noise up the correlation structure inherent in the original data. We find that the resulting permuted data lead to networks with low density and low mean clustering coefficients (reflecting the lack of large modules).

#### Relationship between centralization and density

The relationship between centralization and density (Equation 40) is surprisingly simple for coexpression networks but it does not hold in general networks. For a general network, one can only derive an upper bound for the centralization in terms of the density [35]. As a caveat, we mention that our empirical studies (described below) show that Equation 40 is not very robust with regard to deviations from our theoretical assumptions.

#### Intramodular hub genes cannot be intermediate genes in coexpression networks

The geometric interpretation of gene coexpression network analysis can be used to argue that a gene that lies “intermediate” between two distinct modules cannot be a highly connected intramodular hub gene in either module (see Figure 5B). More precisely, we refer to gene *i* as hub gene in module 1 if its scaled connectivity *K_i_* ^(1)^ is very high (say larger than 0.9). Further, we refer to two modules as distinct if their respective eigengenes have a low correlation, say |cor(*E* ^(1)^,*E* ^(2)^)|<0.3. We refer to gene *i* as intermediate between modules 1 and 2 if it has a moderately high connectivity with both modules, say *K_i_* ^(1)^>0.5 and *K_i_* ^(2)^>0.5.

Equation 33 allows us to translate statements about the scaled intramodular connectivity into statements about the angles between genes and module eigengenes. A gene is an intermediate gene if it has a moderately small angle with both module eigengenes. If the eigengenes are distinct (i.e., the angle between them is large), the intermediate gene cannot have a very small angle with either module eigengene, i.e., it cannot be an intramodular hub gene in either module. A geometric interpretation of this example can be found in Figure 5B.

As an important caveat, we mention that intermediate network genes may well be highly connected “hub” genes if the factorizability property does not hold such as in the entire network comprised of multiple distinct modules.

#### Characterizing module networks where hub genes are significant

For a trait-based gene significance measure, the striking relationship between module significance and hub gene significance (Equation 37) suggests a positive relationship between connectivity and gene significance (high hub gene significance) in modules that are enriched with significant genes (high module significance).

Further, Equation 34 shows that the hub gene significance of a module network is determined by the angle between the module eigengene and the sample trait. This allows us to describe situations when a module has high hub gene significance, i.e., when there is a strong positive relationship between gene significance and intramodular connectivity. In the example provided in Figure 5C and 5D, the angle between *E* and *T2* is small which implies that the hub gene significance with regard to *GS2_i_* = |cor(*x_i_*,*T2*)| is high. By contrast, the angle between *E* and *T1* is large, which implies that the hub gene significance with regard to *GS1_i_* = |cor(*x_i_*,*T1*)| is low.

### Dictionary for Translating between Network Concepts and Their Eigengene-Based Analogs

To facilitate the communication between microarray data analysts and network theorists, we provide a short dictionary for translating between microarray data analysis and network theory terminology. More specifically, for a subset (module) of genes that have high expression factorizability, Table 1 describes the correspondence between general network terms and their eigengene-based counterparts. While our theoretical derivations assume a weighted gene coexpression network, our robustness studies show empirically that many of the findings apply to unweighted networks as well. The summary of empirical robustness studies is described below.

In general, eigengene-based concepts are no substitute for network concepts. It is natural to use network concepts when describing the pairwise relationships between genes and to use eigengene-based network concepts when relating the gene expression profiles to a module eigengene. Since eigengene-based network concepts tend to be relatively simple, they often simplify theoretical derivations. Further, many of them allow one to calculate a statistical significance level (*p*-value) using a correlation or regression based test statistic.

### Real Data Applications

To illustrate the theoretical results we report 4 different microarray data applications. The underlying data sets and R software code can be found on our webpage http://www.genetics.ucla.edu/labs/horvath/ModuleConformity/GeometricInterpretation/.

#### Brain cancer network application

Here we describe a weighted gene coexpression network that was constructed on the basis of 55 microarray samples of glioblastoma (brain cancer) patients. A detailed description of the data, modules, and biological implications can be found in [24]. We defined 6 modules as branches of an average linkage hierarchical cluster tree (Figure 3B). Module membership in the 6 “proper” modules is color-coded by turquoise, blue, brown, yellow, green and red. Grey denotes the color of genes that were not grouped into any of the 6 proper modules. To allow for a comparison, we also report results for the “improper” module comprised of grey genes.

We used the patient survival time as microarray sample trait *T*. We defined a gene significance measure as the absolute value of the correlation between *T* and the gene expression profiles (Equation 4). The module significance was defined as average gene significance (Equation 14). Figure 3C shows that the brown module had the highest module significance. This module was previously found to be enriched with genes that are prognostic of patient survival [24].

By relating the gene significance measure *GS_i_* to the scaled connectivity *K_i_*, we arrive at a hub gene significance measure (Equation 13). As illustrated in Figure 3D and 3E, the hub gene significance is defined as the slope of a regression model without intercept term. The brown module had the highest hub gene significance, see Table 2.

We defined the module eigengene significance (Equation 27) as the absolute value of the correlation between the module eigengene and patient survival time. The brown module eigengene also had the highest eigengene significance: *a_e,t_* ^brown^ = |cor(*E* ^brown^,*T*)| = 0.202. An advantage of the eigengene-based hub gene significance (the eigengene significance) is that it allows one to compute a corresponding p-value. Using a correlation test, we find that the value of the eigengene significance *a_e,t_* ^brown^ is statistically insignificant (*p* = 0.30) in this dataset. However, when we combined these data with an additional data set, we found that the brown module eigengene is significantly related to survival time [24].

We visualize the gene expression profiles of module genes with a heat map plot (Figure 4B) where rows correspond to the genes, the columns to the samples, and the gene expression profiles have been standardized to a mean of 0 and a variance of 1. The heat map colors high and low expression values by red and green, respectively. For a given module, the heat map exhibits characteristic vertical bands that reflect the high correlation among module gene expression profiles. For the 6 proper modules of our brain cancer application, the proportion of variance explained by the first eigengene ranges from 0.59 to 0.71 (Table 2). For the improper grey module genes (defined as genes outside of all proper modules) the proportion of variance explained by the first eigengene is only 0.28. Similarly, when all network genes are used to define an improper module, the proportion of variance explained by the first eigengene is only 0.32. As expected by module construction, we find that the gene expression data of proper modules have high eigengene factorizabilities *EF*(*X*)≥0.97 (Table 2). By contrast, the factorizability of the grey genes (i.e., the genes outside of proper modules) is relatively low (*EF*(*X*) = 0.66).

For each module, Table 2 reports network properties including network size, density, centralization, heterogeneity, mean clustering coefficient, module significance, hub gene significance, and eigengene significance. For the proper (nongrey) modules, we find that the numerical values of the intramodular network concepts and their eigengene-based analogs support our theoretical derivations.

Our empirical results illustrate Observation 2 regarding the relationship between intramodular network concepts and their eigengene-based analogs. Figure 6A–E depict the relationships among centralization, heterogeneity, clustering coefficient, module significance, hub gene significance and their respective eigengene-based analogs when a soft threshold of *β* = 1 is used for the weighted network construction (Equation 2). The analogous results for *β* = 6 are depicted in Figure 6G–K. Figure 6F and 6L depicts the relationship between hub gene significance (Equation 13) and module eigengene significance (Equation 27) for *β* = 1 and *β* = 6, respectively. For completeness, we also report the results for the grey, nonmodule genes in the figures. But since our theoretical results assume proper modules, we exclude the grey genes from the calculation of the squared correlation coefficient *R* ^2^. The summary of a robustness analysis with regard to different soft thresholds *β* and hard thresholds *τ* is reported in Table 3 and Text S1. Overall, we find very high squared correlations (*R* ^2^>0.85), which confirm our theoretical results. Only the *R* ^2^ values for the relationship between clustering coefficient and its eigengene-based analog is decreased if *β*>3.

**Table 3:** Robustness analysis of the brain cancer gene coexpression network results.

**	Weighted networks						Unweighted networks	
Squared correlation R 2 across modules	Soft threshold *β*						Hard threshold *τ*	
Relation	1	2	3	4	5	6	0.7	0.5
*Centralization*≈*Centralization_E_*	1.0	1.0	0.97	0.90	0.87	0.88	0.07	0.93
*Heterogeneity*≈*Heterogeneity_E_*	1.0	1.0	0.99	0.98	0.97	0.96	0.89	0.87
*ClusterCoef_i_*≈*ClusterCoef_E_*	0.99	0.96	0.88	0.74	0.58	0.45	0.04	0.32
*ModuleSignif*≈*ModuleSignif_E_*	0.98	0.91	0.87	0.85	0.85	0.86	0.98	0.98
*HubGeneSignif*≈*HubGeneSignif_E_*	0.96	0.91	0.89	0.90	0.92	0.94	0.93	0.87
*EigengeneSignif*≈*HubGeneSignif*	0.96	0.89	0.87	0.88	0.90	0.92	0.93	0.87
*ClusterCoef* = (1+*Heterogeneity* ^2^)^2^×*Density*	0.99	0.96	0.89	0.76	0.61	0.49	0.006	0.32
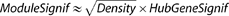	1.0	0.99	0.99	0.98	0.97	0.95	0.85	0.99
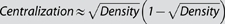	0.90	0.68	0.058	0.016	0.16	0.35	0.20	1.0
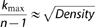	0.94	0.94	0.94	0.94	0.93	0.92	0.95	0.98
*K_i_*≈*a_e_*_,*i*_ (median *R* ^2^)	1.0	1.0	1.0	1.0	1.0	0.99	0.95	0.83

The table reports how the relationships among network concepts change as function of different soft threshold parameters *β* (Equation 2) or hard thresholds (Equation 1) used in the network construction. For each relationship and each network construction method, the table entry reports the squared correlation *R* ^2^ across the proper modules. For within module comparisons the table reports median *R* ^2^ values. Additional details can be found in Text S1.

**Figure 8:**
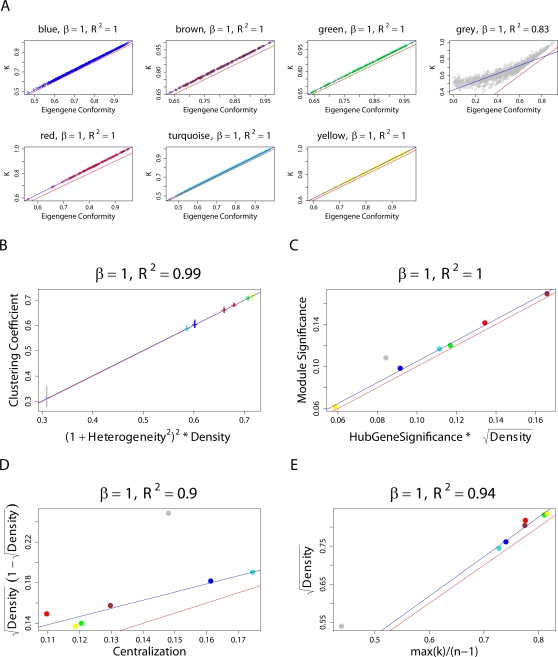
Using the brain cancer data to illustrate Observation 3 regarding the relationships among network concepts. (A) Illustrating Equation 33 regarding the relationship between scaled intramodular connectivity *K_i_* ^(*q*)^ (*y*-axis) and eigengene conformity *a_e_*_,*i*_ (*x*-axis). Each dot corresponds to a gene colored by its module membership. We find a high squared correlation *R* ^2^ even for the grey genes outside properly defined modules. (B) Illustrating Equation 31 regarding the relationship between the clustering coefficient and (1+*Heterogeneity* ^2^)^2^×*Density*. Again each dot represents a gene. The clustering coefficients of grey genes vary more than those of genes in proper modules. The short horizontal lines correspond to the mean clustering coefficient of each module. (C) Illustrating 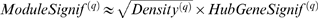 (Equation 37); here each dot corresponds to a module. Since the grey dot corresponds to genes outside of properly defined modules, we have excluded it from the calculation of the squared correlation *R* ^2^. (D) Illustrating 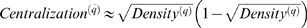 (Equation 40); (E) Illustrating 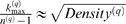 (Equation 38). A reference line (red) with intercept 0 and slope 1 has been added to each plot. The blue line is the regression line through the points representing proper modules (i.e., the grey, non-module genes are left out). A robustness analysis with regard to different network construction methods, e.g., *β*>1, can be found in Text S1.

Figure 8 illustrate the implications of Observation 3 regarding the relationships among network concepts in the cancer coexpression module networks. Figure 8A shows that the scaled connectivity *K_i_* ^(*q*)^ is highly correlated (*R* ^2^>0.99) with *a_e,i_* ^(*q*)^, which illustrates Equation 33. This relationship is highly robust with regard to high soft thresholds *β* as can be seen from Table 3.

Figure 8B illustrates the relationship between the clustering coefficient (the mean corresponds to the short horizontal line) and (1+*Heterogeneity* ^2^)^2^×*Density* (Equation 31). This relationship is diminished for soft thresholds *β*>3 as can be seen from Table 3.

Figure 8C illustrates the relation 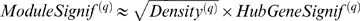 (Equation 37), which is highly robust with regard to different choices of *β* (Table 3). Figure 8D illustrates 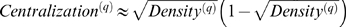 (Equation 40). This relationship is *not* robust with regard to *β*: the *R^2^* value is only 0.058 for *β* = 3. Figure 8E illustrates 
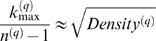
 (Equation 38), which is highly robust with regard to *β* (Table 3).

**Figure 9:**
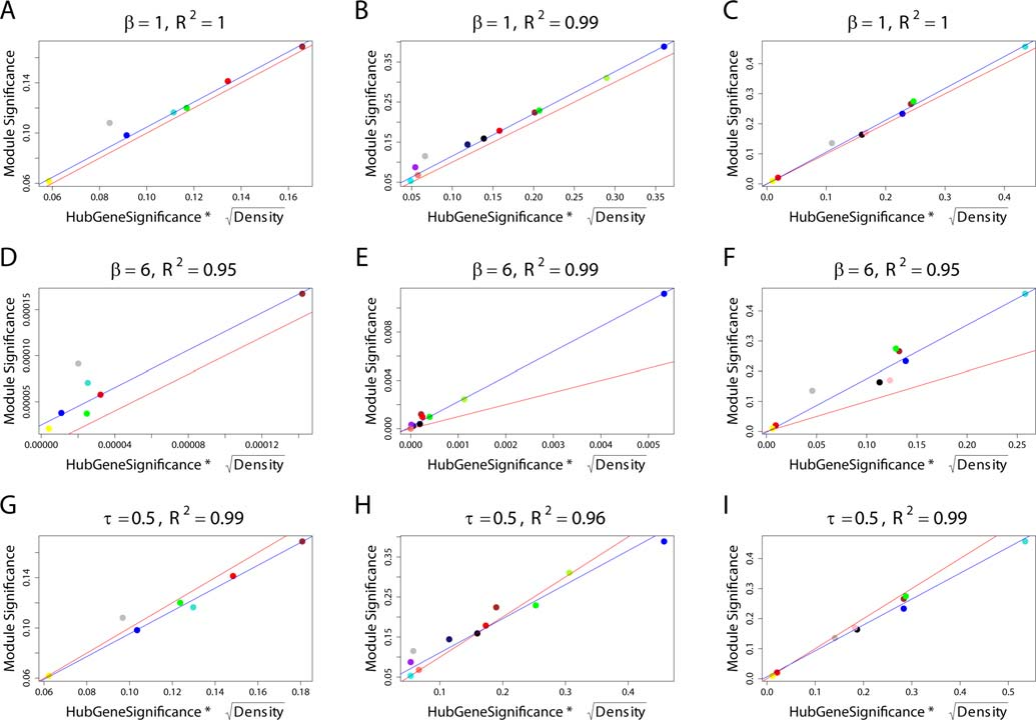
Using three different data (brain cancer, mouse liver, and yeast cell cycle) and three different network construction methods to illustrate Equation 37 regarding the relationship between module significance (*y*-axis) and 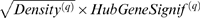 (*x*-axis). Points correspond to modules. The square of the correlation coefficient *R* ^2^ was computed without the grey, improper module. (A,D,G) Corresponding to the brain cancer gene coexpression networks. (B,E,H) Corresponding to mouse liver networks. (C,F,I) Corresponding to yeast networks. (A–C) Corresponding to a weighted network (Equation 2) constructed with soft thresholds *β* = 1. (D–F) Corresponding to *β* = 6. (G–I) Corresponding to an unweighted network (Equation 1) that results from thresholding the correlation matrix at *τ* = 0.5. Overall, we find that the reported relationship is quite robust with respect to our theoretical assumptions (e.g., factorizability). The blue line is the regression line through the points representing proper modules (i.e., the grey, nonmodule genes are left out). A reference line with slope 1 and intercept 0 is shown in red. Additional details can be found in Text S1, Text S2, and Text S3.

Although our theoretical results were derived using relatively restrictive assumptions, we find that most results are robust in the weighted networks, see Figure 9, Table 3, and Text S1. However, in unweighted networks, several relationships have lower *R* ^2^ values and show a strong dependence on the hard threshold *τ* (Table 3).

#### Motivational example: Mouse tissues of an F2 intercross

The mouse tissues came from an F2 intercross between two mouse strains C3H/HeJ and C57BL/6J. The data were already described above and in Figure 1. The 498 genes were part of a body weight related module in liver tissue (the Blue module described in reference [23]). Table 4 presents network concepts and their eigengene-based analogs in the different tissue networks. As predicted by Observation 2, we find a close relationship between the two types of network concepts if the eigengene factorizability of the corresponding network is close to 1. This example also illustrates that our results apply to coexpression networks comprised of relatively few genes (here 498 genes).

**Table 4:** Values of network concepts in the different mouse gender/tissue networks reported in Figure 1.

	Female liver		Female adipose		Female brain		Female muscle	
Network concept	Network	Eigengene	Network	Eigengene	Network	Eigengene	Network	Eigengene
Factorizability	0.92	0.91	0.72	0.46	0.89	0.82	0.79	0.68
Density	0.39	0.39	0.23	0.14	0.32	0.27	0.24	0.19
Centralization	0.19	0.19	0.11	0.19	0.34	0.23	0.17	0.22
Heterogeneity	0.18	0.19	0.22	0.59	0.36	0.54	0.32	0.57
Mean cluster coef	0.42	0.42	0.27	0.26	0.41	0.46	0.30	0.33

	Male liver		Male adipose		Male brain		Male muscle	
Network concept	Network	Eigengene	Network	Eigengene	Network	Eigengene	Network	Eigengene
Factorizability	0.93	0.92	0.76	0.59	0.73	0.46	0.76	0.48
Density	0.37	0.36	0.23	0.16	0.21	0.13	0.25	0.16
Centralization	0.19	0.21	0.15	0.21	0.21	0.20	0.12	0.21
Heterogeneity	0.28	0.32	0.28	0.65	0.27	0.67	0.30	0.64
Mean cluster coef	0.43	0.44	0.27	0.31	0.25	0.26	0.31	0.31

For each network, the table reports the network factorizability *F*(*A*), the eigengene factorizability *EF*(*X*), network concepts, and their eigengene-based analogs. Here we use a soft threshold *β* = 1 (Equation 2).

#### Mouse gene coexpression network application

Here we focus on the female mouse liver tissues of the above-mentioned F2 mouse cross. Specifically, 135 female mice were used to construct a weighted network comprised of 3,400 highly connected genes. The biological significance and gene ontology enrichment analysis of the 12 modules in this large network is described in [23]. In Text S2, Table 5, and Figure 9, we focus on the relationships among the network concepts. We find that many of our theoretical results hold approximately even if the expression factorizability is low. Table 5 shows how the relationship (*R* ^2^ values) between network concepts and their eigengene-based analogs depend on the soft threshold *β*. Overall, we find that our theoretical results are highly robust in weighted networks. The relationship between the clustering coefficient and its eigengene-based analog is diminished (down to 0.44) for *β*>3. The relationship between heterogeneity and its eigengene-based analog is diminished (down to 0.54 when *β*<3).

**Table 5:** Robustness analysis of the mouse coexpression network.

**	Weighted networks						Unweighted networks	
Squared correlation *R^2^* across modules	Soft threshold *β*						Hard threshold *τ*	
Relation	1	2	3	4	5	6	0.65	0.5
*Centralization*≈*Centralization_E_*	0.69	0.74	0.90	0.95	0.94	0.92	0.007	0.66
*Heterogeneity*≈*Heterogeneity_E_*	0.54	0.59	0.71	0.82	0.88	0.86	0.30	0.33
*ClusterCoef_i_*≈*ClusterCoef_E_*	0.94	0.84	0.70	0.59	0.50	0.44	0.09	0.33
*ModuleSignif*≈*ModuleSignif_E_*	0.96	0.96	0.96	0.97	0.98	0.99	0.96	0.96
*HubGeneSignif*≈*HubGeneSignif_E_*	0.98	0.98	0.98	0.99	1.0	1.0.	0.88	0.91
*EigengeneSignif*≈*HubGeneSignif*	0.98	0.98	0.98	0.99	1.0	1.0	0.89	0.92
*ClusterCoef* = (1+*Heterogeneity* ^2^)^2^×*Density*	0.89	0.78	0.70	0.62	0.54	0.48	0.08	0.31
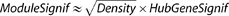	0.99	0.99	0.99	0.99	0.99	0.99	0.90	0.96
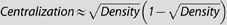	0.52	0.21	0.43	0.73	0.82	0.84	0.60	0.82
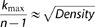	0.95	0.97	0.97	0.98	0.98	0.98	0.93	0.80
*K_i_*≈*a_e_*_,*i*_ (median *R* ^2^)	1.0	0.99	0.98	0.96	0.95	0.94	0.74	0.86

The table reports how the relationships among network concepts change as function of different soft threshold parameters *β* (Equation 2) or hard thresholds *τ* (Equation 1) used in the network construction. For each relationship and each network construction method, the table entry reports the squared correlation *R* ^2^ across the proper modules. For within module comparisons the table reports median *R* ^2^ values. Additional details can be found in Text S2.

The relation 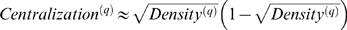 (Equation 40) has a relatively low *R* ^2^ value (down to 0.21) for low values of *β*≤3 but the other relationships among network concepts are highly robust with respect to *β*. For unweighted networks, the *R* ^2^ values tend to be lower and several relationships show a marked dependency on the hard threshold *τ* (Table 5).

#### Yeast gene coexpression network application

In Text S3, we illustrate our theoretical derivations using a yeast gene coexpression network. The yeast microarray data were derived from experiments designed to study the cell cycle [54]. A detailed biological description of the modules and the importance of intramodular connectivity can be found in previous work [33]. In Text S3 and in Figure 9, we use a gene significance measure that encodes knock-out essentiality, i.e., *GS_i_* = 1 if the *i*th gene is known to be essential and 0 otherwise. In contrast to the other applications, *this gene significance measure is not based on a sample trait*. Our theoretical derivations for relating module significance to hub gene significance (Equation 37) assumed a sample-trait based gene significance measure. Although this important assumption is violated for knock-out essentiality, it is striking that the relationship between hub gene significance and module significance can still be observed empirically (Figure 9).

Table 6 shows how the relationship (squared correlation *R* ^2^) between network concepts and their eigengene-based analogs depend on the soft threshold *β*. Overall, we find that our theoretical results are highly robust in weighted networks. The relation 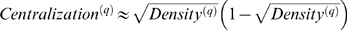 (Equation 40) breaks down for *β* = 3 or 4 but the other relationships among network concepts are highly robust with respect to *β*. For unweighted networks, the *R* ^2^ values tend to be lower and several relationships show a marked dependency on the hard threshold *τ* (Table 6).

**Table 6:** Robustness analysis of the yeast coexpression network.

	Weighted networks							Unweighted networks	
Squared correlation *R^2^* across modules	Soft threshold *β*							Hard threshold *τ*	
Relation	1	2	3	4	5	6	7	0.65	0.5
*Centralization*≈*Centralization_E_*	0.99	0.97	0.97	0.98	0.98	0.98	0.98	0.53	0.60
*Heterogeneity*≈*Heterogeneity_E_*	0.86	0.92	0.94	0.94	0.93	0.92	0.91	0.13	0.006
*ClusterCoef_i_*≈*ClusterCoef_E_*	0.98	0.97	0.94	0.92	0.89	0.86	0.82	0.18	0.25
*ClusterCoef* = (1+*Heterogeneity* ^2^)^2^×*Density*	0.99	0.97	0.95	0.92	0.89	0.86	0.83	0.21	0.27
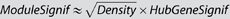	1.0	0.99	0.98	0.98	0.97	0.95	0.94	0.99	0.99
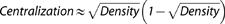	0.51	0.24	0.04	0.06	0.33	0.53	0.68	0.76	0.98
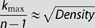	0.89	0.91	0.93	0.94	0.95	0.96	0.96	0.51	0.20
*K_i_*≈*a_e_*_,*i*_ (median *R* ^2^)	1.0	0.99	0.99	0.98	0.97	0.97	0.96	0.93	0.92

The table reports how the relationships among network concepts change as function of different soft threshold parameters *β* (Equation 2) or hard thresholds *τ* (Equation 1) used in the network construction. For each relationship and each network construction method, the table entry reports the squared correlation *R* ^2^ across the proper modules. For within module comparisons the table reports median *R* ^2^ values. Additional details can be found in Text S3.

## Discussion

Network theoretic methods and concepts are increasingly used for the systems biologic analysis of microarray data. We illustrate how network concepts can be used for describing large correlation matrices and for arriving at biologically plausible data reduction techniques. Many alternative approaches for defining gene coexpression networks are possible, e.g., [13], [55]–[61]. Here we define the network adjacency and the gene significance measure in terms of correlations since this allows us to interpret pairwise relations in terms of angles between scaled versions of the variables. For example, the sample trait based gene significance measure of the *i*th gene is determined by the angle between the *i*th gene expression profile and the sample trait *T* (Equation 4); the scaled intramodular connectivity of the *i*th gene (Equation 33) is determined by the angle between the *i*th gene expression profile and the module eigengene; the hub gene significance (Equation 34) is determined by the angle between module eigengene and the sample trait.

The geometric interpretation of gene coexpression network analysis reveals a deep connection to other statistical methods. Since it projects the gene expressions profiles onto the hypersphere in an *m*-dimensional Euclidean space, network analysis can be considered a special case of directional statistics. When focusing on the use of module eigengenes, network analysis can be considered a variant of oblique factor analysis.

A high level view of modules and their centroids (eigengenes) can be used to define eigengene networks [52]. High correlations (small angles) between module eigengenes may suggest close relationships between the corresponding pathways. A low level view of a single module allows us to provide a geometric interpretation of intramodular network concepts. We use the singular value decomposition of module expression data to characterize approximately factorizable gene coexpression networks, i.e., adjacency matrices that satisfy *a_ij_* ^(*q*)^≈*CF_i_* ^(*q*)^ *CF_j_* ^(*q*)^. We provide an intuitive formula of the conformity *CF_i_* ^(*q*)^≈|cor(*x_i_* ^(*q*)^,*E* ^(*q*)^)|*^β^*. Since the module eigengene *E* ^(*q*)^ summarizes the overall behavior of the module, the eigengene conformity |cor(*x_i_* ^(*q*)^,*E* ^(*q*)^)|*^β^* measures how well gene *i* conforms to the overall module. This insight led us to coin the term “conformity”. Using the singular values, we propose a measure of eigengene factorizability (Equation 24) that is analogous to the proportion of variance explained by the module eigengene (Equation 22). We provide a geometric interpretation of network factorizability in Figure 5A.

The derivation of Observation 1 in the Methods section highlights a theoretical advantage of the soft-thresholding approach (Equation 2); the resulting weighted network maintains the approximate factorizability of the underlying correlation matrix: *a_ij_* ^(*q*)^ = |cor(*x_i_* ^(*q*)^,*x_j_* ^(q)^)|*^β^*≈|cor(*x_i_* ^(*q*)^,*E* ^(*q*)^)cor(*x_j_* ^(*q*)^,*E* ^(*q*)^)|*^β^* = |cor(*x_i_* ^(*q*)^,*E* ^(*q*)^)|*^β^*|cor(*x_j_* ^(*q*)^,*E* ^(*q*)^)|*^β^*.

Using multiple different gene coexpression networks from mouse tissues, brain cancer, and yeast, we provide empirical evidence that coexpression modules tend to have high eigengene factorizability and that the maximum conformity assumption (Equation 32) is satisfied for low powers of *β*.

We propose eigengene-based analogs of network concepts (Equation 30). While network concepts are functions of the adjacency matrix, eigengene-based network concepts are analogous functions of the eigengene conformities |cor(*x_i_* ^(*q*)^,*E* ^(*q*)^)|*^β^*. Algebraically, eigengene-based network concepts are closely related to “approximate conformity based” network concepts [8] but they allow for a geometric interpretation.

We use the correspondence between intramodular network concepts and their eigengene-based analogs to provide a geometric interpretation of network concepts. Observation 2 states that network concepts in weighted gene coexpression module networks are approximately equal to their eigengene-based analogs. A major theoretical advantage of eigengene-based network concepts is that they reveal simple relationships. To arrive at particularly simple relationships, we make the maximum conformity assumption (Equation 32) for the results presented in the main text. Table 1 provides a rough dictionary for translating between gene coexpression network analysis and the singular value decomposition if the underlying expression data have high eigengene factorizability (say *EF*(*X* ^(*q*)^)>0.95) and if the maximum conformity assumption (Equation 32) is satisfied. However, even if the maximum conformity assumption does not hold, one can still find simple relationships among the network concepts (Equation 49).

The geometric interpretation of gene coexpression networks facilitates the derivation of several results that should be interesting to network theorists. For example, we argue that highly connected intramodular hub genes cannot be intermediate between two distinct coexpression modules (Figure 5B). The geometric interpretation is particularly useful when studying gene significance and module significance measures that are based on a microarray sample trait (Equation 4). To study the relationship between connectivity and gene significance, we propose a novel measure of hub gene significance (Equation 13). We find that the hub gene significance of a module network is determined by the angle between the module eigengene and the microarray sample trait (Equation 34). Our geometric interpretation of coexpression networks allows us to describe situations when a module has low hub gene significance (Figure 5C and 5D). Our theoretical derivations for relating module significance to hub gene significance (Equation 37) assumes a gene significance measure based on a sample trait. Although this important assumption is violated for the gene significance measure (knock-out essentiality) in the yeast network, it is striking that the relationship between hub gene significance and module significance can still be observed in this application (Figure 9).

We provide a robustness analysis that shows that many of our theoretical results apply even if our underlying assumptions are not satisfied (Figures 6 and 9, Tables 3, 5, and 6, Text S1, Text S2, and Text S3). We find that the correspondence between network concepts and their eigengene-based analogs is often better in weighted networks than in unweighted networks. Further, we find that the results in weighted networks tend to be more robust than those in unweighted networks with regard to changing the network construction thresholds *β* and *τ*, respectively. Thus, weighted coexpression networks are preferable over unweighted networks when a geometric interpretation of network concepts is desirable.

The correspondence between coexpression module networks and the singular value decomposition (Table 1) can break down when a high soft threshold is used for constructing a weighted network or when dealing with an unweighted network. Thus, eigengene-based concepts do not replace network concepts when describing interaction patterns among genes.

While this article has a theoretical bent, we illustrate the results on three different microarray data sets (human, mouse, and yeast) that are described in our online R software tutorials, in Text S1, Text S2, and Text S3. Our theoretical results also apply to networks comprised of genes that are highly correlated with a sample trait. The key assumption underlying our results is high eigengene factorizability *EF*(*X* ^(*q*)^). To illustrate this point, Text S4 describes a brain cancer network comprised of the 500 genes with highest absolute correlation with brain cancer survival time. Our results illustrate that the geometric interpretation of gene coexpression networks has important theoretical and practical implications that may guide the development and application of network methods.

## Materials and Methods

### Network Concept Functions and Fundamental Network Concepts

Analogous to [8], we define a *network concept function* to be function of a square matrix *M* = [*M_ij_*] (1≤*i*,*j*≤*n*) and/or a corresponding vector *G* = (*G*_1_,…,*G_n_*). For example, *M* could be the adjacency matrix (with diagonal set to 0) and *G* could be a corresponding gene significance measure.

We make use of the following network concept functions:
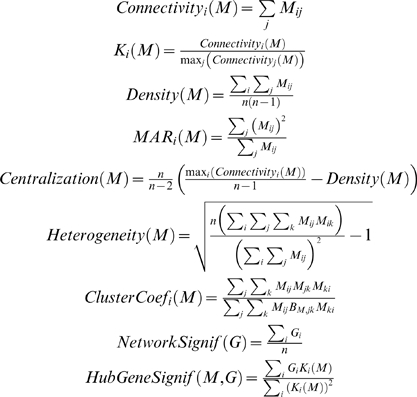
(42)where the components of matrix *B_M_* in the denominator of the clustering coefficient function are given by *b_ij_* = 1 if *i*≠*j* and *b_ii_* = Ind(*m_ii_*>0). Here the indicator function Ind(·) takes on the value 1 if the condition is satisfied and 0 otherwise.

According to our convention, the diagonal elements of the adjacency matrix are set to 1. Therefore, the diagonal elements of *A–I* (where *I* denotes the identity matrix) equal 0. Now we are ready to define the (fundamental) network concepts that are studied in this article.

**Definition of Fundamental Network Concepts:** *The fundamental network concepts of a network A are defined by evaluating the network functions (* *Equation 42* *) on A–I and the gene significance measure GS, i.e.,*

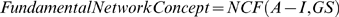



For example, the connectivity is given by
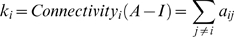
(43)


We define an **intramodular network concept** *NCF*(*A* ^(*q*)^−*I*,*GS* ^(*q*)^) by evaluating the network concept function on the restricted adjacency matrix *A* ^(*q*)^ and the restricted gene significance measure *GS* ^(*q*)^.

We will now define eigengene-based network concepts. Using the eigengene-based adjacency matrix *A_E_* ^(*q*)^ = *a_e_* ^(*q*)^(*a_e_* ^(*q*)^)*^T^* (Equation 28) and the eigengene-based gene significance measure *GS_E,i_* ^(*q*)^ = *a_e,i_* ^(*q*)^ *a_e,t_* ^(*q*)^ (Equation 29), we define an **eigengene-based network concept** as *NCF*(*A_E_* ^(*q*)^,*GS_E_* ^(*q*)^).

As example, consider the eigengene-based connectivity given by
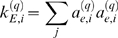
(44)


### Deriving Observation 1: Expression Data with High Eigengene Factorizability Lead to Approximately Factorizable Networks

Here we derive Observation 1, which characterizes approximately factorizable gene coexpression module networks. To simplify the presentation, we omit the superscripts (*q*) in the following, e.g., we will write *EF*(*X*) instead of *EF*(*X* ^(*q*)^). We will argue that if the eigengene factorizability *EF*(*X*) is close to 1, the adjacencies of the weighted coexpression module network *A* = |cor(*X*)|*^β^* and the trait-based gene significance measure *GS_i_* = |cor(*x_i_*,*T*)|*^β^* can be factored as follows
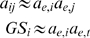
(45)where
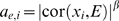
(46)

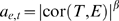
(47)


Since our gene coexpression networks are defined with respect to the correlation matrix [cor(*x_i_*,*x_j_*)], which is scale-invariant, we can assume that the gene expression profiles have been scaled as follows: 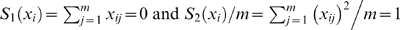 where *m* is the number of microarray samples. Then one can derive the following relationships
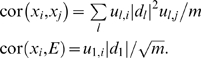
Note that *u*_1,*i*_|*d*_1_|^2^ *u*_1,*j*_/*m* = cor(*x_i_*,*E*)cor(*x_j_*,*E*). Using the fact that *U* is an orthogonal matrix, it is straightforward to show that
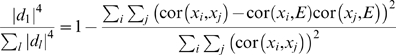



This equation motivates us to propose the following measure of **eigengene factorizability**:
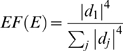
(48)Note that 0≤*EF*(*E*)≤1. By definition *EF*(*E*)≈1 implies that
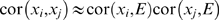
By raising both sides of this equation to a power *β*, we find
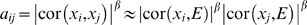
The last step highlights an important theoretical advantage of the soft thresholding method: it preserves the approximate factorizability of the underlying correlation matrix.

An alternative, possibly more direct way of motivating the observation is based on the insight that the squared singular values *|d_l_|^2^* correspond to the eigenvalues of the correlation matrix *COR* = [cor(*x_i_*,*x_j_*)]. For high values of *EF*(*E*), the correlation matrix can be factored as follows
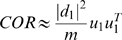
where u_1_ denotes an eigenvector of length 1.

### Relationships among Network Concepts When the Maximum Conformity Assumption Does Not Hold

Here we describe relationships among eigengene-based network concepts if the maximum conformity assumption does not hold (i.e., *a_e_*_,max_^(*q*)^<<1). It is straightforward to derive the following relationships among eigengene-based network concepts:
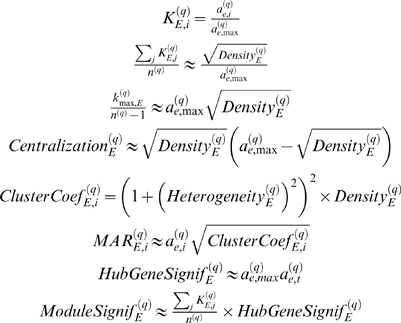
(49)Observation 2 can be used to derive the following

#### Observation 4

*If A^(q)^ = |cor(X^(q)^)|^β^ and the eigengene factorizability is close to 1 (EF(X^(q)^)≈1), the relationships among eigengene-based concepts approximately apply to their network analogs as well.*

For example, we find that
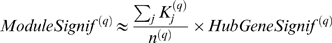



### Deriving the Geometric Interpretation of Factorizability

In the following we provide details on our geometric interpretation of the factorizability. To simplify the notation, we sometimes drop the superscript (*q*) in the following expressions. We denote by *θ_l,i_* the angle between the right singular vector *v_l_* (Equation 20) and the *i*th gene expression profile *x_i_*. The smaller the angle *θ_l,i_*, the bigger the correlation cor(*v_l_*,*x_i_*) = cos(*θ_l_*_,*i*_). Using 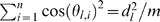, one can reexpress the eigengene factorizability (Equation 24) as follows
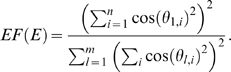
(50)


Thus, *EF*(*X* ^(*q*)^)≈1 if the module gene expressions *x_i_* are approximately orthogonal (cos(*θ_l_*_,*i*_)≈0) to the right singular vectors ***v*** *_l_* for *l*≥2, i.e., if on average the gene expression profiles point in the direction of the module eigengene *v*_1_ = *E*.

Under this assumption, we provide a rough geometric intuition of *a_ij_*≈*a_e,i_a_e,j_* (Equation 25) depicted in Figure 5A. We denote by *θ_i_* = *θ*_1,*i*_ the angle between the module eigengene *E* and the *i*th gene expression profile and by *θ_ij_* the angle between gene expression profiles *i* and *j*. Using the assumptions described in Figure 5A, *θ_ij_*≈|*θ_i_*±*θ_j_*| and sin(*θ_i_*) sin(*θ_j_*)≈0, we find that
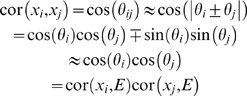
(51)i.e., the correlation matrix is approximately factorizable.

### Heterogeneity Increases with the Soft Threshold *β*

Here we prove that the eigengene-based heterogeneity increases with the soft threshold *β* (Equation 2). Recall that 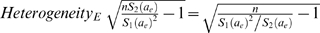 (Equation 30) which implies that it is a decreasing function of
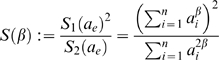
(52)Note that *a_i_* = |cor(*x_i_*,*E*)| is a nonnegative number.

To prove that the heterogeneity increases with *β*, it suffices to prove the following

**Proposition:** *Let {a_i_, i = 1,…,n} be a group of nonnegative number and β>1 then the following inequality holds*:
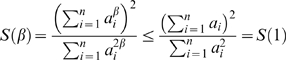
(53)


To prove the Proposition, we will make use of the following

**Lemma:** *Let {u_i_, i = 1,…,n} and {v_i_, i = 1,…,n} be groups of nonnegative numbers, and θ be a number 0≤θ<*1. *Then the following inequality holds:*

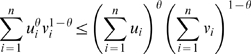
(54)The Lemma can be proved with Hölder's inequality, which is given by
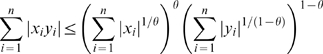
(55)We use the Lemma with *θ*_1_ = *β*/(*2β*−1), *u_i_* = *a_i_*, and *v_i_* = *a_i_^2β^* to derive
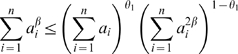
Further, we use the Lemma with *θ*_2_ = (*2β*−2)/(2*β*−1), *u_i_* = *a_i_*, and *v_i_* = *a_i_^2β^* to derive
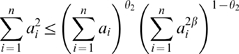
By squaring the first inequality and multiplying it with the second inequality, we arrive at
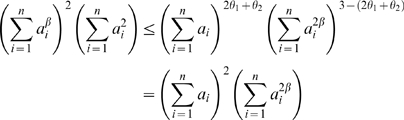
since 2*θ*_1_+*θ*_2_ = 2 and 3−(2*θ*_1_+*θ*_2_) = 1. The last inequality completes the proof since it is equivalent to the inequality in Equation 53.

## Supporting Information

Text S1 Robustness Analysis of the Brain Cancer Gene Coexpression Network. This supporting text provides a detailed analysis of the brain cancer gene coexpression network. The robustness analysis illustrates how the results change with regard to different network construction methods.
(3.83 MB PDF)

Text S2 Robustness Analysis of the Mouse Gene Coexpression Network. This supporting text provides a detailed analysis of the mouse tissue gene coexpression network. The robustness analysis illustrates how the results change with regard to different network construction methods.
(3.76 MB PDF)

Text S3 Robustness Analysis of the Yeast Gene Coexpression Network. This supporting text provides a detailed analysis of the yeast cell cycle gene coexpression network. The robustness analysis illustrates how the results change with regard to different network construction methods.
(2.62 MB PDF)

Text S4 Brain Cancer Network Comprised of 500 Prognostic Genes. Here we analyze a brain cancer network comprised of the 500 genes with highest absolute correlation with brain cancer survival time. The results illustrate that our theoretical results also apply to small networks comprised of sample trait related genes. The robustness analysis illustrates how the results change with regard to different network construction methods.
(0.38 MB PDF)
