# Supplementary material for: Geometric Interpretation of Gene Coexpression Network Analysis
Source: PLoS Comput Biol. 2008 Aug 15;4(8):e1000117. doi: 10.1371/journal.pcbi.1000117 (PMC2446438; doi:10.1371/journal.pcbi.1000117)
Supplement: Text S1 — Robustness Analysis of the Brain Cancer Gene Coexpression Network. This supporting text provides a detailed analysis of the brain cancer gene coexpression network. The robustness analysis illustrates how the results change with regard to different network construction methods. (3.83 MB PDF) [file pcbi.1000117.s001.pdf]

Text S1: Robustness Analysis of the Brain Cancer Gene Co-expression Network

Jun Dong and Steve Horvath*

Dept. of Human Genetics, David Geffen School of Medicine, UCLA
Dept. of Biostatistics, School of Public Health, UCLA
*Correspondence: shorvath@mednet.ucla.edu

Abstract

This is a supplement of the article "Geometric Interpretation of Gene Co-Expression Network Analysis". Here we illustrate our theoretical results using gene expression data from the brain cancer application. In particular, we study the robustness of our theoretical findings with regard to alternative methods of constructing a network. We describe the results for weighted co-expression networks constructed using different soft-thresholds $\beta \geq 1$ in

$$a_{ij} = |cor(\boldsymbol{x}_i, \boldsymbol{x}_j)|^{\beta}.$$

Further, we report the analogous findings for unweighted networks constructed on the basis of

$$a_{ij} = Ind(|cor(\boldsymbol{x}_i, \boldsymbol{x}_j)| \geq \tau),$$

where τ is the 'hard' threshold parameter, and $Ind(\cdot)$ is the indicator function taking value of 1 if the condition is true, and 0 otherwise. We provide empirical evidence that co-expression modules tend to have high eigengene factorizability and that the maximum conformity assumption is satisfied for low powers of β. Our robustness analysis shows that many of our theoretical results apply even if our underlying assumptions are not satisfied.

1 Brain Cancer Gene Co-expression Network Application

In this supplement, we use a weighted gene co-expression network that was constructed on the basis of 55 microarray samples of glioblastoma (brain cancer) patients. A detailed description of the data and methods can be found in (Horvath *et al.*, 2006). To facilitate a comparison with earlier work, we keep the module definition from (Horvath *et al.*, 2006) for the 5 proper modules. We then also added a new red module by lowering our minimum module size requirement since this module is also biologically significant with respect to the trait we consider. We defined 6 modules as branches of an average linkage hierarchical cluster tree (dendrogram), see Figure 3B in the main article. Module membership in the 6 'proper' modules is color-coded by turquoise, blue, brown, yellow, green and red. The biological meaning of these modules is discussed in (Horvath *et al.*, 2006). Grey denotes the color of genes that were not grouped into any of the 6 proper modules. To allow for a comparison, we also report results for the 'improper' module comprised of grey, non-module genes.

We have constructed weighted networks with $\beta = 1, 2, 3, 4, 5$ and 6, and unweighted networks with $\tau = 0.7$ and 0.5. For the unweighted networks, we use the eigengene-based network concepts of weighted networks with $\beta = 1$ for demonstration purposes.

2 Robustness of Module Definition

Our module definition was based on the topological overlap measure (TOM) in conjunction with average linkage hierarchical clustering (refer to the Methods Supplement). In this application, we used TOM of an weighted network with power $\beta = 6$. To facilitate a comparison, we used this network module assignment for the other weighted and unweighted network analysis as well. In each of the figure below, the upper panel is the dendrogram of the average linkage hierarchical clustering method using the specific network construction parameter, and the lower panel shows genes colored by their module membership. As the figures show, our module definition is quite robust with regard to the choice of network construction methods.

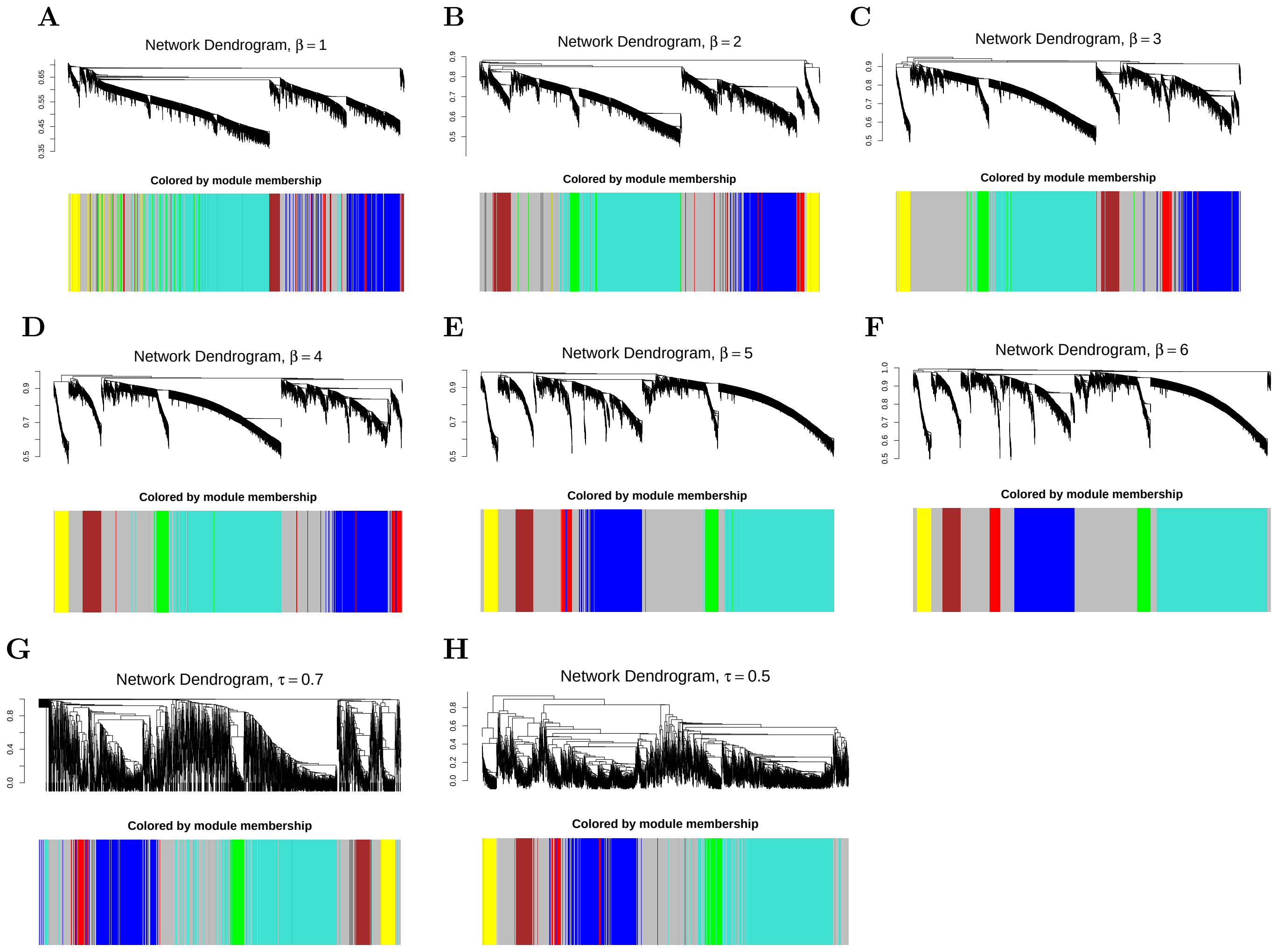

Figure 1: Robustness of module definition. In each figure, genes are colored by their module membership.

3 Summary of Robustness Analysis

To make this supplement self-contained, we repeat the following summary table from our main article. In the rest of this supplement, we provide the details on how we arrived at the R^2 values of this table.

Table 1: Robustness Analysis of the Brain Cancer Gene Co-expression Network Results. The table reports how the relation between network concepts changes as function of different soft threshold parameters β or hard thresholds used in the network construction. For each relationship and each network construction method, the table entry reports the squared correlation R^2 across the proper modules. For within module comparisons the table reports median R^2 values.

Squared Correlation R^2 Across Modules. Relation	Weighted Networks Soft Threshold β 1	2	3	4	5	6	Unweighted Net Hard Threshold τ 0.7	0.5
$Centralization \approx Centralization_E$	1.0	1.0	0.97	0.90	0.87	0.88	0.07	0.93
$Heterogeneity \approx Heterogeneity_E$	1.0	1.0	0.99	0.98	0.97	0.96	0.89	0.87
$ClusterCoef_i \approx ClusterCoef_E$	0.99	0.96	0.88	0.74	0.58	0.45	0.04	0.32
$ModuleSignif \approx ModuleSignif_E$	0.98	0.91	0.87	0.85	0.85	0.86	0.98	0.98
$HubGeneSignif \approx HubGeneSignif_E$	0.96	0.91	0.89	0.90	0.92	0.94	0.93	0.87
$EigengeneSignif \approx HubGeneSignif$	0.96	0.89	0.87	0.88	0.90	0.92	0.93	0.87
$ClusterCoef_i \approx \left(1 + (Heterogeneity)^2\right)^2 \times Density$	0.99	0.96	0.89	0.76	0.61	0.49	0.006	0.32
$ModuleSignif \approx \sqrt{Density} \times HubGeneSignif$	1.0	0.99	0.99	0.98	0.97	0.95	0.85	0.99
$Centralization \approx \sqrt{Density}(1 - \sqrt{Density})$	0.90	0.68	0.058	0.016	0.16	0.35	0.20	1.0
$\frac{k_{max}}{n-1} \approx \sqrt{Density}$	0.94	0.94	0.94	0.94	0.93	0.92	0.95	0.98
$K_i \approx a_{e,i}$ (median R^2)	1.0	1.0	1.0	1.0	1.0	0.99	0.95	0.83

Overall, we find very high R^2 values (> 0.85), which confirm our theoretical results. Only the R^2 values for the relationship between clustering coefficient and its eigengene-based analog is decreased if $\beta > 3$.

The scaled connectivity $K_i^{(q)}$ is highly correlated ($R^2 > 0.99$) with $a_{e,i}^{(q)}$. This relationship is highly robust with regard to high soft thresholds β as can be seen from Table 1.

The relationship between the clustering coefficient and $(1 + Heterogeneity^2)^2 \times Density$ is diminished for soft thresholds $\beta > 3$. The relationship $ModuleSignif^{(q)} \approx \sqrt{Density^{(q)}} \times HubGeneSignif^{(q)}$ is highly robust with regard to different choices of β. The relationship $Centralization^{(q)} \approx \sqrt{Density^{(q)}}(1 - \sqrt{Density^{(q)}})$ is *not* robust with regard to β: the R^2 value is only 0.058 for $\beta = 3$. The relationship

$\frac{k_{max}^{(q)}}{n^{(q)}-1} \approx \sqrt{Density^{(q)}}$ is highly robust with regard to β.

Although our theoretical results were derived using relatively restrictive assumptions, we find that most results are robust in the weighted networks. However, in unweighted networks, several relationships have lower R^2 values and show a strong dependence on the hard threshold τ (Table 1).

Our robustness analysis shows that many of our theoretical results apply even if our underlying assumptions are not satisfied. We find that the correspondence between network concepts and their eigengene-based analogs is often better in weighted networks than in unweighted networks. Further, we find that results in weighted networks tend to be more robust than those in unweighted networks with regard to changing the network construction thresholds β and τ, respectively. Thus, weighted co-expression networks are preferable over unweighted networks when a geometric interpretation of network concepts is desirable.

The correspondence between co-expression module networks and the singular value decomposition (Table 1 in the main article) can break down when a high soft threshold is used for constructing a weighted network or when dealing with an unweighted network. Thus, eigengene-based concepts do not replace network concepts when describing interaction patterns between genes.

4 Weighted Gene Co-Expression Network Results for $\beta = 1$

Table 2: Values of network concepts for a weighted network constructed with a soft threshold of $\beta = 1$.

Module	blue	brown	green	grey	red	turquoise	yellow
Size ($n^{(q)}$)	606	185	136	1313	105	1112	143
Eigengene Factorizability ($EF(X^{(q)})$)	0.974	0.986	0.991	0.662	0.981	0.978	0.991
$VarExplained(\boldsymbol{E}^{(q)})$	0.591	0.655	0.702	0.278	0.677	0.57	0.71
$max(a_{e,i})$	0.971	0.966	0.975	0.908	0.951	0.979	0.976
$Density$	0.58	0.647	0.692	0.291	0.668	0.554	0.699
$Density_E$	0.581	0.652	0.699	0.233	0.678	0.554	0.706
$Centralization$	0.161	0.13	0.121	0.148	0.11	0.174	0.119
$Centralization_E$	0.16	0.132	0.121	0.206	0.111	0.175	0.119
$Heterogeneity$	0.137	0.1	0.105	0.174	0.0913	0.17	0.11
$Heterogeneity_E$	0.138	0.101	0.105	0.44	0.0906	0.171	0.111
$Mean(ClusterCoef)$	0.603	0.66	0.707	0.323	0.68	0.587	0.716
$ClusterCoef_E$	0.603	0.662	0.71	0.331	0.683	0.587	0.718
$ModuleSignif$	0.088	0.117	0.21	0.105	0.158	0.142	0.0652
$ModuleSignif_E$	0.018	0.0927	0.211	0.00759	0.157	0.13	0.0388
$HubGeneSignif$	0.107	0.142	0.249	0.151	0.184	0.185	0.0737
$HubGeneSignif_E$	0.0229	0.111	0.247	0.0143	0.182	0.171	0.0452
$EigengeneSignif$	0.0236	0.115	0.253	0.0157	0.192	0.175	0.0463

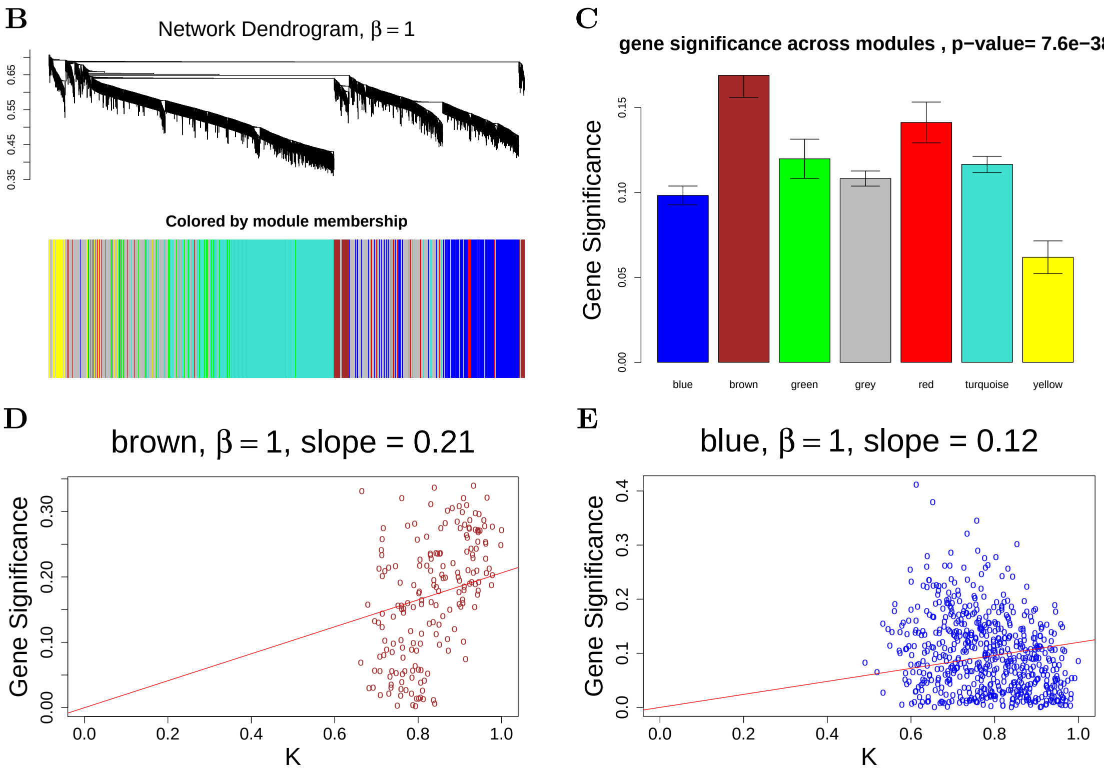

Figure 2: This figure is analogous to Figure 3 in the main article. The only difference is that we use a different dissimilarity for constructing the cluster tree in Figure B. Specifically, we use the topological overlap matrix based dissimilarity corresponding to a weighted network constructed with $\beta = 1$. Figure B depicts the hierarchical cluster tree of genes. Modules correspond to branches of the tree. The branches and module genes are assigned a color as can be seen from the color-bands underneath the tree. Grey denotes genes outside of proper modules. Figure C shows the module significance (average gene significance) of the modules. The underlying gene significance is defined with respect to the patient survival time. Figures D and E show scatter plots of gene significance GS (y-axis) versus scaled connectivity K (x-axis) in the brown and blue module, respectively. The hub gene significance is defined as the slope of the red line, which results from a regression model without an intercept term.

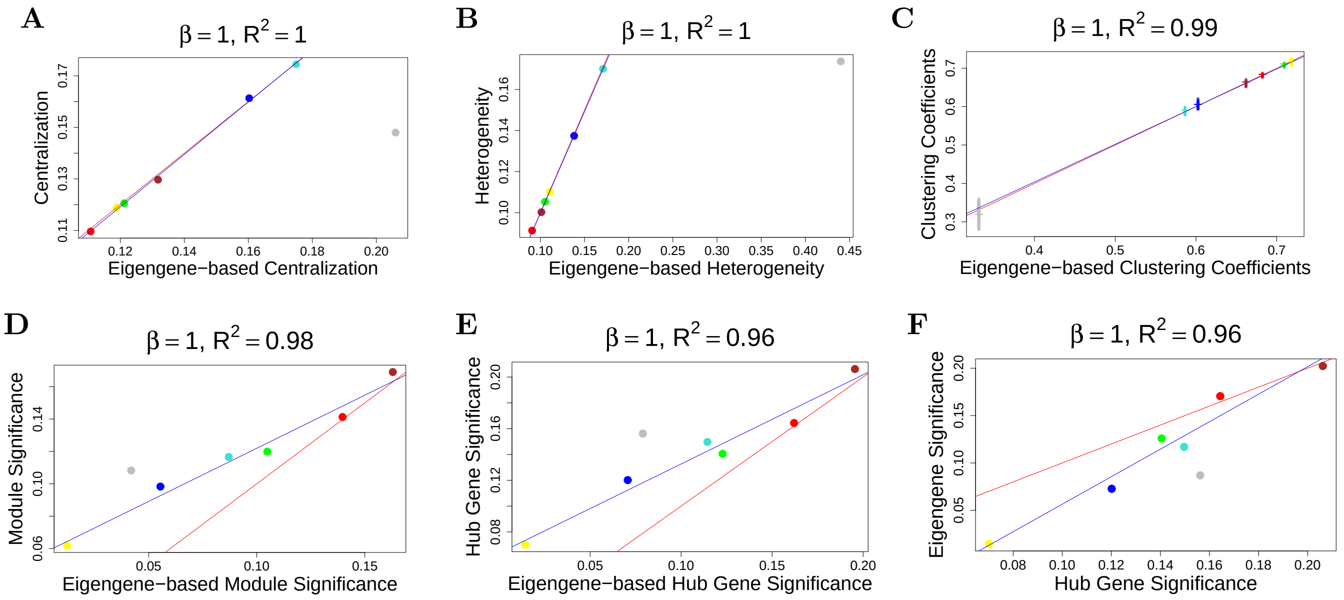

Figure 3: This figure is analogous to Figure 6 in the main article, corresponding to a weighted network constructed with a soft threshold of $\beta = 1$. It illustrates Observation 2 regarding the relationship between network concepts (y-axis) and their eigengene-based analogs (x-axis) in the brain cancer data. Each point corresponds to a module. Figure A: Centralization (y-axis) versus eigengene-based Centralization$_E$ (x-axis); analogous plots for Figure B: Heterogeneity; Figure C: clustering coefficient; Figure D: module significance; and Figure E: hub gene significance. Figure F illustrates the relationship between eigengene significance and hub gene significance. The blue line is the regression line through the points representing proper modules (i.e., the grey, non-module genes are left out). While the red reference line (slope 1, intercept 0) does not always fit well, we observe high squared correlations R^2 between network concepts and their analogs. Since the grey point corresponds to the genes outside properly defined modules, we did not include it in calculations.

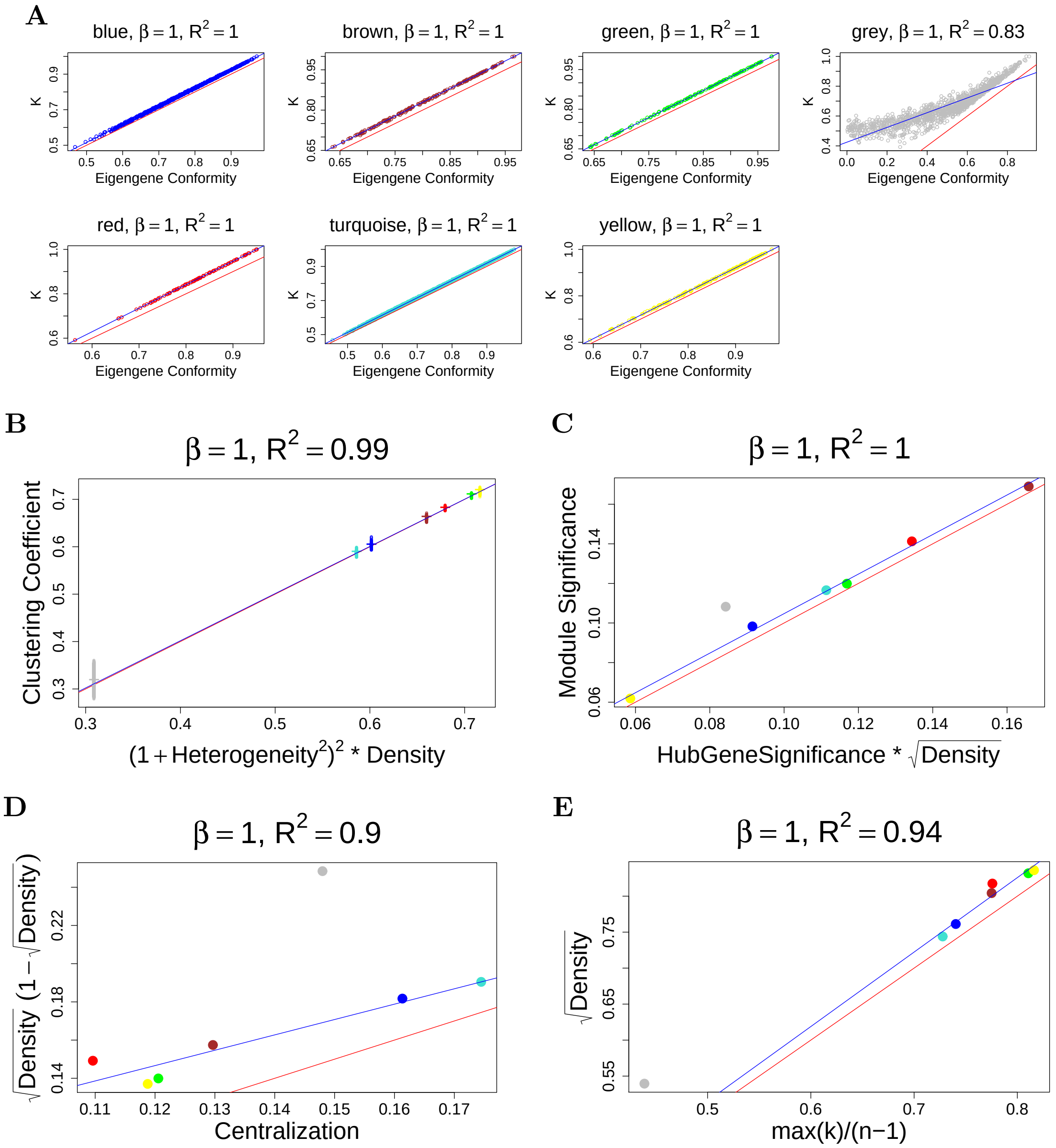

Figure 4: This figure is analogous to Figure 8 in the main article, corresponding to a weighted network constructed with a soft threshold of $\beta = 1$. It illustrates Observation 3 regarding the relationships among network concepts. Figure A illustrates Equation (33) in the main article regarding the relationship between scaled intramodular connectivity $K_i^{(q)}$ (y-axis) and eigengene conformity $a_{e,i}$ (x-axis). Each dot corresponds to a gene colored by its module membership. We find a high squared correlation R^2 even for the grey genes outside properly defined modules. Figure B illustrates Equation (31) regarding the relationship between the clustering coefficient and $(1 + Heterogeneity^2)^2 \times Density$. Again each dot represents a gene. The clustering coefficients of grey genes vary more than those of genes in proper modules. The short horizontal lines correspond to the mean clustering coefficient of each module. Figure C illustrates $ModuleSignif^{(q)} \approx \sqrt{Density^{(q)}} \times HubGeneSignif^{(q)}$ (Equation 37); here each dot corresponds to a module. Since the grey dot corresponds to genes outside of properly defined modules, we have excluded it from the calculation of the squared correlation R^2. Figure D illustrates $Centralization^{(q)} \approx \sqrt{Density^{(q)}}(1 - \sqrt{Density^{(q)}})$ (Equation 40); Figure E illustrates $\frac{k_{max}^{(q)}}{n^{(q)}-1} \approx \sqrt{Density^{(q)}}$ (Equation 38). A reference line (red) with intercept 0 and slope 1 has been added to each plot. The blue line is the regression line through the points representing proper modules (i.e., the grey, non-module genes are left out).

5 Weighted Gene Co-Expression Network Results for $\beta = 2$

Table 3: Values of network concepts for a weighted network constructed with a soft threshold of $\beta = 2$.

Module	blue	brown	green	grey	red	turquoise	yellow
Size ($n^{(q)}$)	606	185	136	1313	105	1112	143
Eigengene Factorizability ($EF(X^{(q)})$)	0.974	0.986	0.991	0.662	0.981	0.978	0.991
$VarExplained(\boldsymbol{E}^{(q)})$	0.591	0.655	0.702	0.278	0.677	0.57	0.71
$max(a_{e,i})$	0.943	0.934	0.951	0.825	0.904	0.958	0.953
$Density$	0.358	0.432	0.493	0.116	0.462	0.332	0.505
$Density_E$	0.35	0.432	0.496	0.0771	0.463	0.325	0.507
$Centralization$	0.201	0.181	0.175	0.12	0.153	0.215	0.172
$Centralization_E$	0.209	0.186	0.179	0.152	0.158	0.222	0.176
$Heterogeneity$	0.253	0.194	0.2	0.287	0.169	0.32	0.209
$Heterogeneity_E$	0.269	0.2	0.203	0.68	0.176	0.336	0.213
$Mean(ClusterCoef)$	0.409	0.466	0.534	0.162	0.492	0.404	0.55
$ClusterCoef_E$	0.402	0.465	0.534	0.165	0.487	0.402	0.55
$ModuleSignif$	0.0118	0.0188	0.0525	0.0167	0.0311	0.0281	0.00652
$ModuleSignif_E$	0.000329	0.00868	0.0449	6.87e-05	0.0248	0.0175	0.00152
$HubGeneSignif$	0.0155	0.0276	0.0723	0.0294	0.0415	0.0438	0.00766
$HubGeneSignif_E$	0.000525	0.0124	0.0608	0.000204	0.0332	0.0294	0.00204
$EigengeneSignif$	0.000557	0.0133	0.0639	0.000248	0.0367	0.0307	0.00214

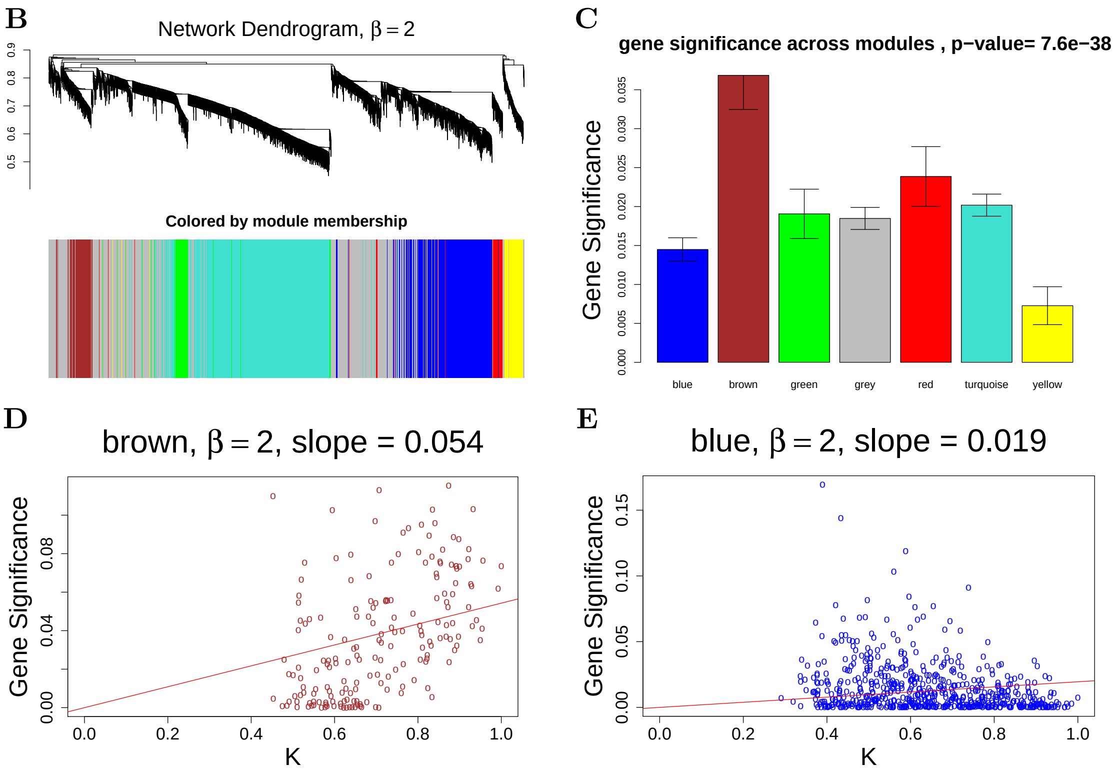

Figure 5: This figure is analogous to Figure 3 in the main article. The only difference is that we use a different dissimilarity for constructing the cluster tree in Figure B. Specifically, we use the topological overlap matrix based dissimilarity corresponding to a weighted network constructed with $\beta = 2$. Figure B depicts the hierarchical cluster tree of genes. Modules correspond to branches of the tree. The branches and module genes are assigned a color as can be seen from the color-bands underneath the tree. Grey denotes genes outside of proper modules. Figure C shows the module significance (average gene significance) of the modules. The underlying gene significance is defined with respect to the patient survival time. Figures D and E show scatter plots of gene significance GS (y-axis) versus scaled connectivity K (x-axis) in the brown and blue module, respectively. The hub gene significance is defined as the slope of the red line, which results from a regression model without an intercept term.

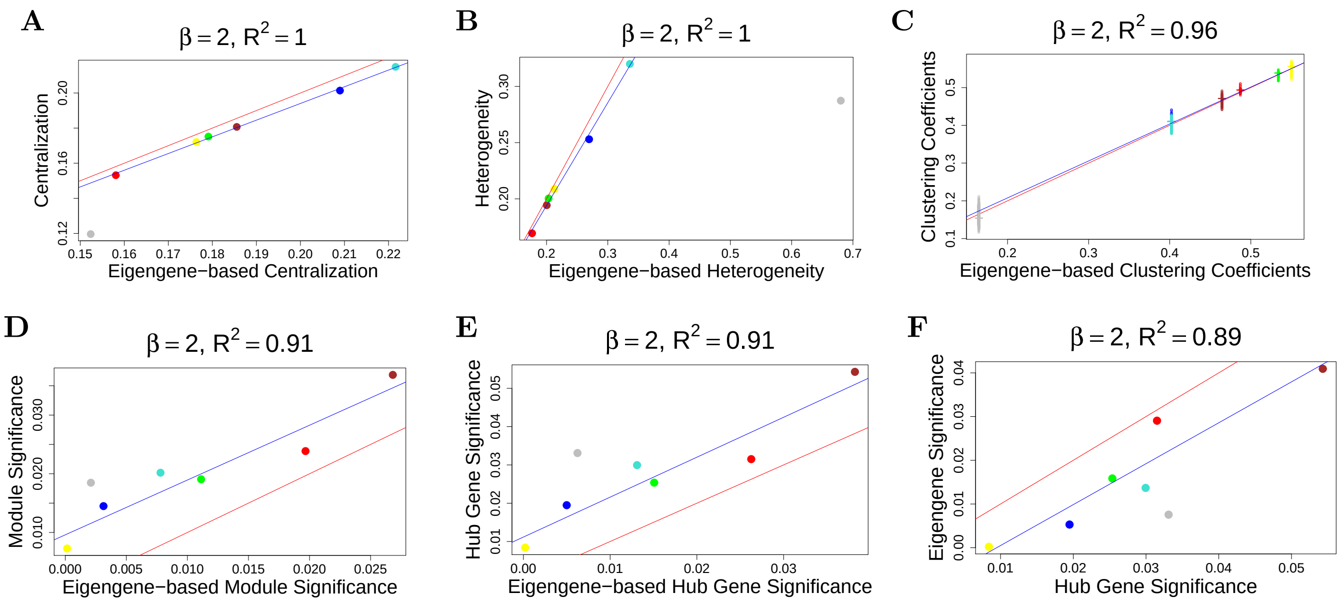

Figure 6: This figure is analogous to Figure 6 in the main article, corresponding to a weighted network constructed with a soft threshold of $\beta = 2$. It illustrates Observation 2 regarding the relationship between network concepts (y-axis) and their eigengene-based analogs (x-axis) in the brain cancer data. Each point corresponds to a module. Figure A: Centralization (y-axis) versus eigengene-based Centralization$_E$ (x-axis); analogous plots for Figure B: Heterogeneity; Figure C: clustering coefficient; Figure D: module significance; and Figure E: hub gene significance. Figure F illustrates the relationship between eigengene significance and hub gene significance. The blue line is the regression line through the points representing proper modules (i.e., the grey, non-module genes are left out). While the red reference line (slope 1, intercept 0) does not always fit well, we observe high squared correlations R^2 between network concepts and their analogs. Since the grey point corresponds to the genes outside properly defined modules, we did not include it in calculations.

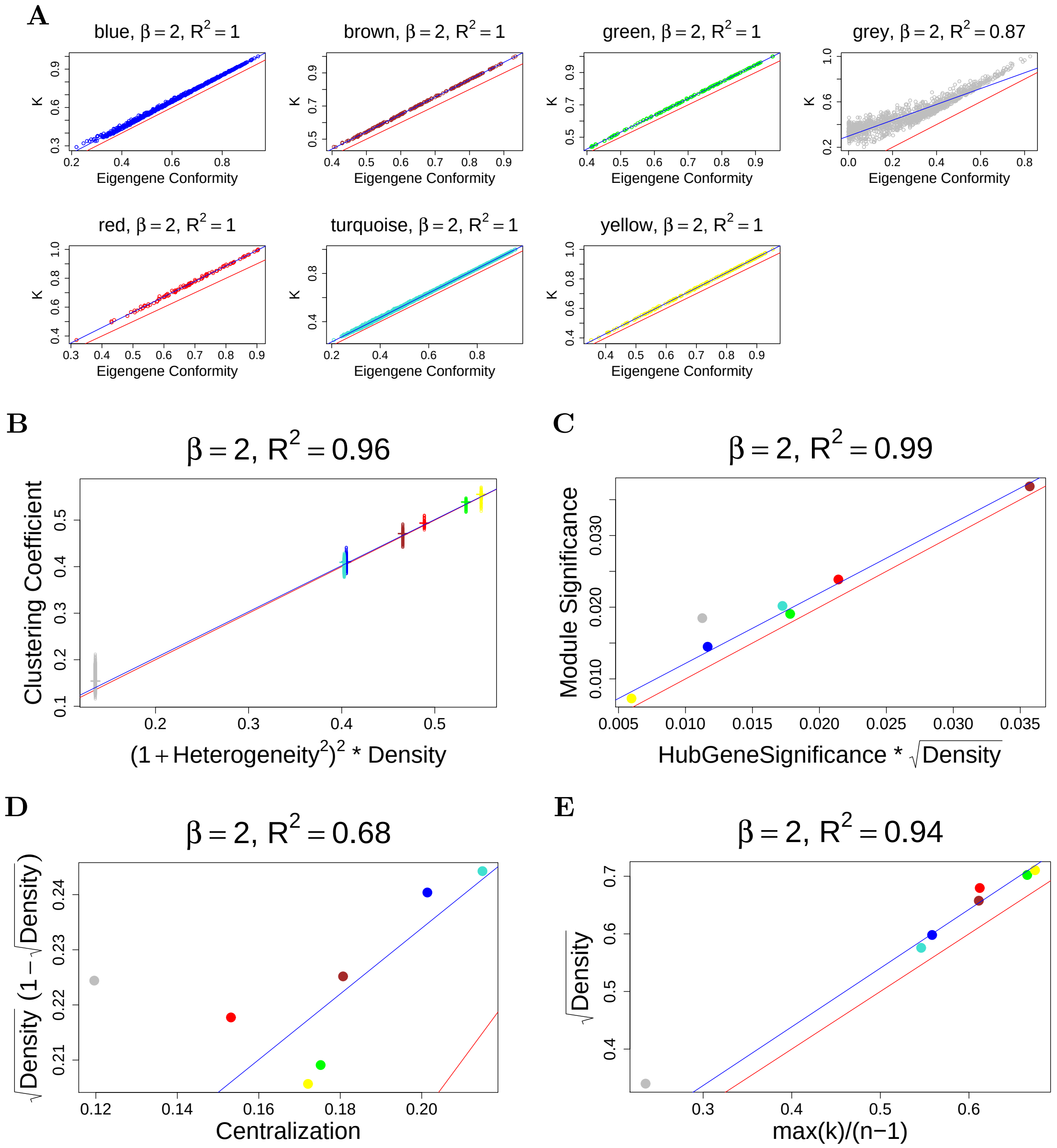

Figure 7: This figure is analogous to Figure 8 in the main article, corresponding to a weighted network constructed with a soft threshold of $\beta = 2$. It illustrates Observation 3 regarding the relationships among network concepts. Figure A illustrates Equation (33) in the main article regarding the relationship between scaled intramodular connectivity $K_i^{(q)}$ (y-axis) and eigengene conformity $a_{e,i}$ (x-axis). Each dot corresponds to a gene colored by its module membership. We find a high squared correlation R^2 even for the grey genes outside properly defined modules. Figure B illustrates Equation (31) regarding the relationship between the clustering coefficient and $(1 + Heterogeneity^2)^2 \times Density$. Again each dot represents a gene. The clustering coefficients of grey genes vary more than those of genes in proper modules. The short horizontal lines correspond to the mean clustering coefficient of each module. Figure C illustrates $ModuleSignif^{(q)} \approx \sqrt{Density^{(q)}} \times HubGeneSignif^{(q)}$ (Equation 37); here each dot corresponds to a module. Since the grey dot corresponds to genes outside of properly defined modules, we have excluded it from the calculation of the squared correlation R^2. Figure D illustrates $Centralization^{(q)} \approx \sqrt{Density^{(q)}}(1 - \sqrt{Density^{(q)}})$ (Equation 40); Figure E illustrates $\frac{k_{max}^{(q)}}{n^{(q)}-1} \approx \sqrt{Density^{(q)}}$ (Equation 38). A reference line (red) with intercept 0 and slope 1 has been added to each plot. The blue line is the regression line through the points representing proper modules (i.e., the grey, non-module genes are left out).

6 Weighted Gene Co-Expression Network Results for $\beta = 3$

Table 4: Values of network concepts for a weighted network constructed with a soft threshold of $\beta = 3$.

Module	blue	brown	green	grey	red	turquoise	yellow
Size ($n^{(q)}$)	606	185	136	1313	105	1112	143
Eigengene Factorizability ($EF(X^{(q)})$)	0.974	0.986	0.991	0.662	0.981	0.978	0.991
$VarExplained(\boldsymbol{E}^{(q)})$	0.591	0.655	0.702	0.278	0.677	0.57	0.71
$max(a_{e,i})$	0.916	0.902	0.928	0.75	0.859	0.937	0.93
$Density$	0.232	0.297	0.361	0.0532	0.328	0.211	0.375
$Density_E$	0.219	0.291	0.359	0.0298	0.321	0.201	0.372
$Centralization$	0.197	0.194	0.195	0.0842	0.165	0.211	0.192
$Centralization_E$	0.211	0.199	0.202	0.0999	0.172	0.22	0.2
$Heterogeneity$	0.356	0.284	0.287	0.377	0.239	0.458	0.297
$Heterogeneity_E$	0.395	0.297	0.295	0.881	0.259	0.493	0.307
$Mean(ClusterCoef)$	0.3	0.349	0.423	0.098	0.375	0.309	0.444
$ClusterCoef_E$	0.291	0.343	0.421	0.094	0.362	0.311	0.443
$ModuleSignif$	0.00203	0.00352	0.0142	0.00331	0.007	0.00654	0.000844
$ModuleSignif_E$	6.14e-06	0.000821	0.00965	6.72e-07	0.00396	0.00241	6.03e-05
$HubGeneSignif$	0.00267	0.00612	0.0221	0.00665	0.0103	0.0114	0.000955
$HubGeneSignif_E$	1.2e-05	0.00138	0.015	2.92e-06	0.00604	0.00504	9.23e-05
$EigengeneSignif$	1.31e-05	0.00153	0.0162	3.9e-06	0.00703	0.00537	9.92e-05

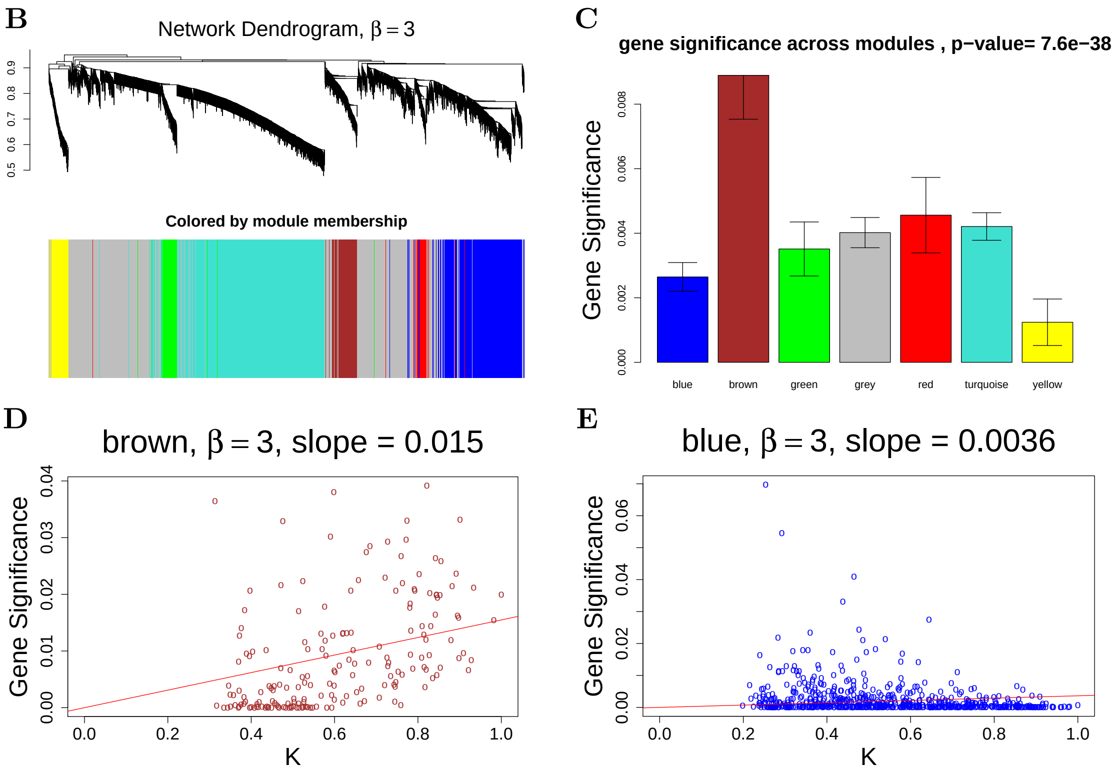

Figure 8: This figure is analogous to Figure 3 in the main article. The only difference is that we use a different dissimilarity for constructing the cluster tree in Figure B. Specifically, we use the topological overlap matrix based dissimilarity corresponding to a weighted network constructed with $\beta = 3$. Figure B depicts the hierarchical cluster tree of genes. Modules correspond to branches of the tree. The branches and module genes are assigned a color as can be seen from the color-bands underneath the tree. Grey denotes genes outside of proper modules. Figure C shows the module significance (average gene significance) of the modules. The underlying gene significance is defined with respect to the patient survival time. Figures D and E show scatter plots of gene significance GS (y-axis) versus scaled connectivity K (x-axis) in the brown and blue module, respectively. The hub gene significance is defined as the slope of the red line, which results from a regression model without an intercept term.

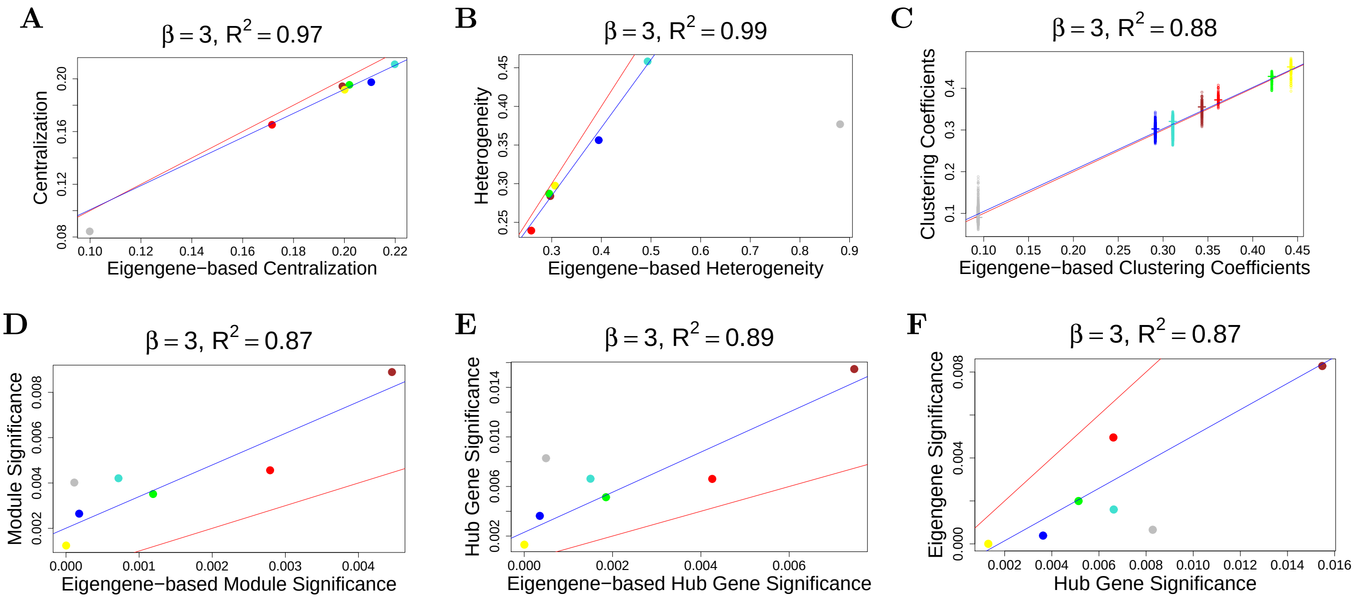

Figure 9: This figure is analogous to Figure 6 in the main article, corresponding to a weighted network constructed with a soft threshold of $\beta = 3$. It illustrates Observation 2 regarding the relationship between network concepts (y-axis) and their eigengene-based analogs (x-axis) in the brain cancer data. Each point corresponds to a module. Figure A: Centralization (y-axis) versus eigengene-based Centralization$_E$ (x-axis); analogous plots for Figure B: Heterogeneity; Figure C: clustering coefficient; Figure D: module significance; and Figure E: hub gene significance. Figure F illustrates the relationship between eigengene significance and hub gene significance. The blue line is the regression line through the points representing proper modules (i.e., the grey, non-module genes are left out). While the red reference line (slope 1, intercept 0) does not always fit well, we observe high squared correlations R^2 between network concepts and their analogs. Since the grey point corresponds to the genes outside properly defined modules, we did not include it in calculations.

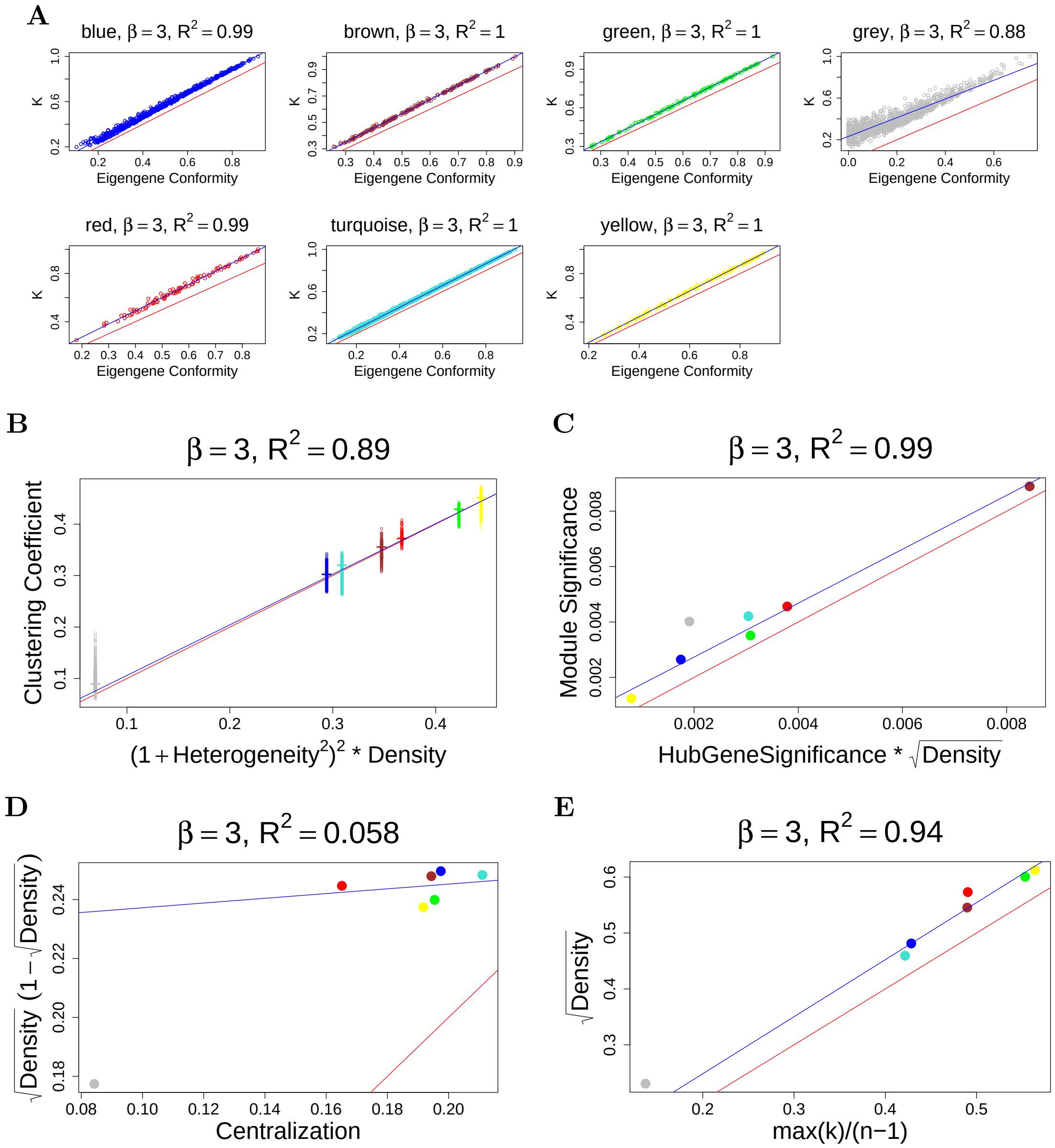

Figure 10: This figure is analogous to Figure 8 in the main article, corresponding to a weighted network constructed with a soft threshold of $\beta = 3$. It illustrates Observation 3 regarding the relationships among network concepts. Figure A illustrates Equation (33) in the main article regarding the relationship between scaled intramodular connectivity $K_i^{(q)}$ (y-axis) and eigengene conformity $a_{e,i}$ (x-axis). Each dot corresponds to a gene colored by its module membership. We find a high squared correlation R^2 even for the grey genes outside properly defined modules. Figure B illustrates Equation (31) regarding the relationship between the clustering coefficient and $(1 + Heterogeneity^2)^2 \times Density$. Again each dot represents a gene. The clustering coefficients of grey genes vary more than those of genes in proper modules. The short horizontal lines correspond to the mean clustering coefficient of each module. Figure C illustrates $ModuleSignif^{(q)} \approx \sqrt{Density^{(q)}} \times HubGeneSignif^{(q)}$ (Equation 37); here each dot corresponds to a module. Since the grey dot corresponds to genes outside of properly defined modules, we have excluded it from the calculation of the squared correlation R^2. Figure D illustrates $Centralization^{(q)} \approx \sqrt{Density^{(q)}}(1 - \sqrt{Density^{(q)}})$ (Equation 40); Figure E illustrates $\frac{k_{max}^{(q)}}{n^{(q)}-1} \approx \sqrt{Density^{(q)}}$ (Equation 38). A reference line (red) with intercept 0 and slope 1 has been added to each plot. The blue line is the regression line through the points representing proper modules (i.e., the grey, non-module genes are left out).

7 Weighted Gene Co-Expression Network Results for $\beta = 4$

Table 5: Values of network concepts for a weighted network constructed with a soft threshold of $\beta = 4$.

Module	blue	brown	green	grey	red	turquoise	yellow
Size ($n^{(q)}$)	606	185	136	1313	105	1112	143
Eigengene Factorizability ($EF(X^{(q)})$)	0.974	0.986	0.991	0.662	0.981	0.978	0.991
$VarExplained(\boldsymbol{E}^{(q)})$	0.591	0.655	0.702	0.278	0.677	0.57	0.71
$max(a_{e,i})$	0.889	0.872	0.905	0.681	0.817	0.917	0.908
$Density$	0.156	0.21	0.27	0.0269	0.239	0.141	0.285
$Density_E$	0.141	0.201	0.265	0.0127	0.225	0.131	0.279
$Centralization$	0.178	0.191	0.198	0.0578	0.162	0.194	0.194
$Centralization_E$	0.194	0.193	0.206	0.0642	0.168	0.201	0.205
$Heterogeneity$	0.451	0.37	0.367	0.454	0.304	0.588	0.378
$Heterogeneity_E$	0.515	0.392	0.38	1.07	0.339	0.641	0.394
$Mean(ClusterCoef)$	0.234	0.272	0.348	0.0671	0.297	0.253	0.372
$ClusterCoef_E$	0.225	0.265	0.345	0.0587	0.278	0.26	0.369
$ModuleSignif$	0.000421	0.000722	0.00406	0.000761	0.00175	0.00168	0.000133
$ModuleSignif_E$	1.16e-07	7.84e-05	0.0021	6.91e-09	0.000637	0.00034	2.42e-06
$HubGeneSignif$	0.00052	0.00145	0.00699	0.00165	0.00274	0.0031	0.000137
$HubGeneSignif_E$	2.76e-07	0.000153	0.0037	4.18e-08	0.0011	0.000863	4.17e-06
$EigengeneSignif$	3.1e-07	0.000176	0.00409	6.13e-08	0.00135	0.000941	4.59e-06

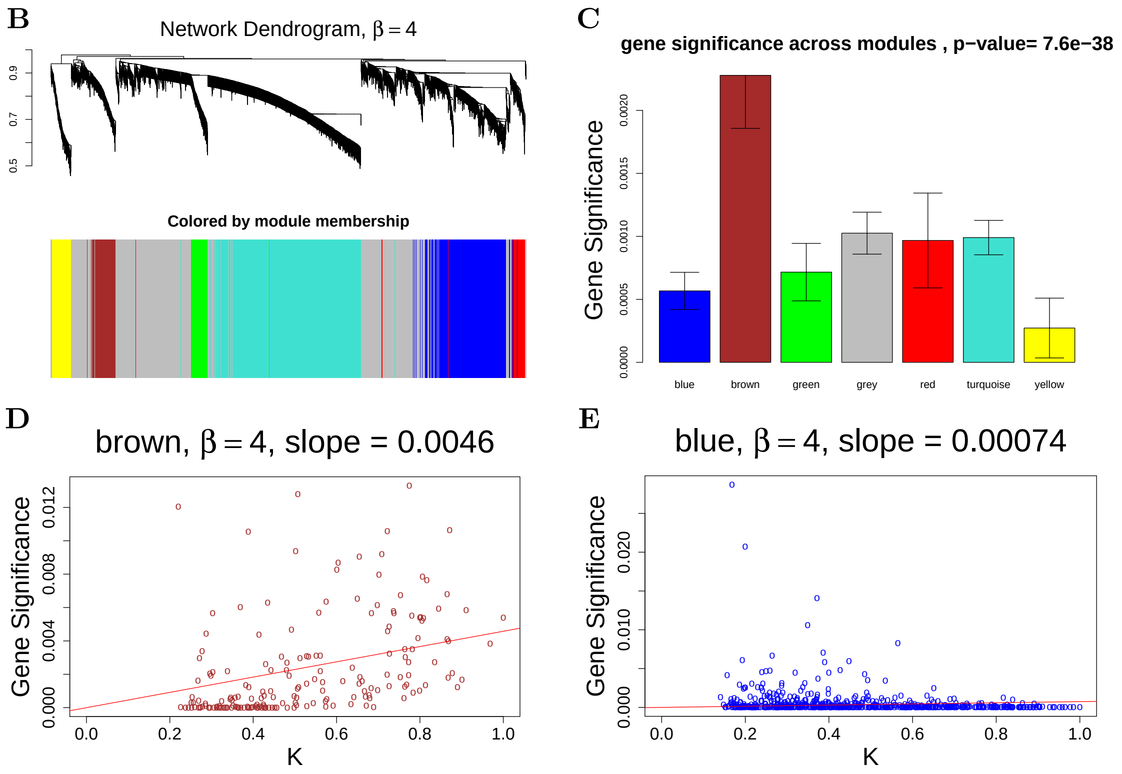

Figure 11: This figure is analogous to Figure 3 in the main article. The only difference is that we use a different dissimilarity for constructing the cluster tree in Figure B. Specifically, we use the topological overlap matrix based dissimilarity corresponding to a weighted network constructed with $\beta = 4$. Figure B depicts the hierarchical cluster tree of genes. Modules correspond to branches of the tree. The branches and module genes are assigned a color as can be seen from the color-bands underneath the tree. Grey denotes genes outside of proper modules. Figure C shows the module significance (average gene significance) of the modules. The underlying gene significance is defined with respect to the patient survival time. Figures D and E show scatter plots of gene significance GS (y-axis) versus scaled connectivity K (x-axis) in the brown and blue module, respectively. The hub gene significance is defined as the slope of the red line, which results from a regression model without an intercept term.

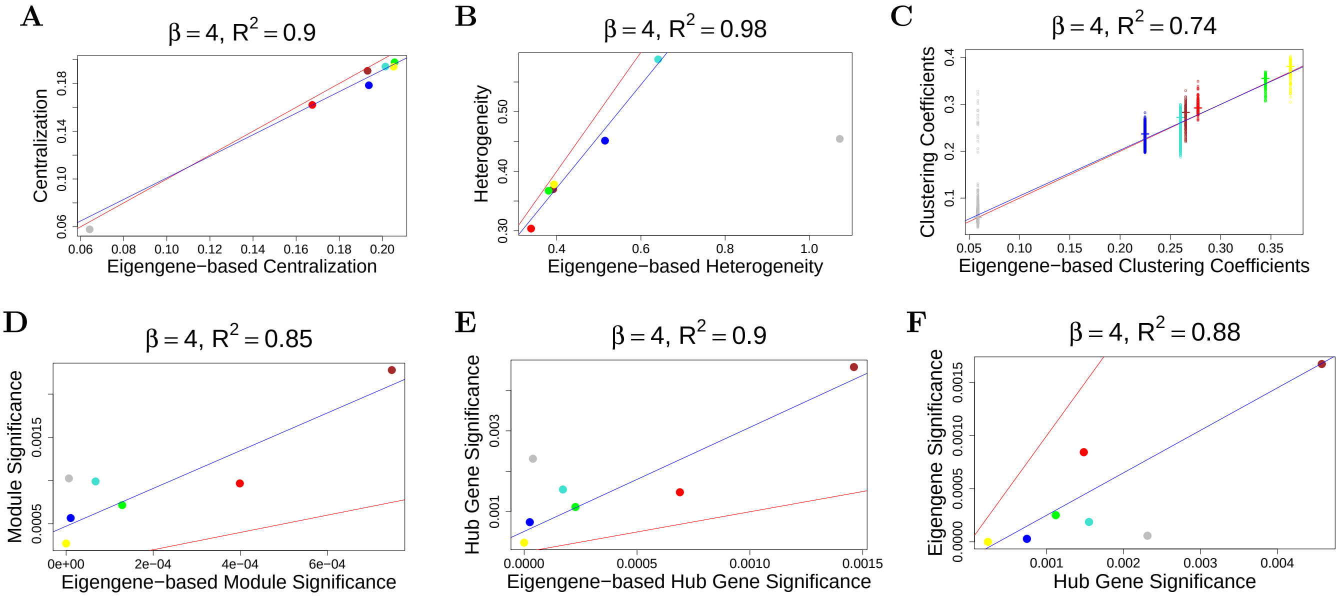

Figure 12: This figure is analogous to Figure 6 in the main article, corresponding to a weighted network constructed with a soft threshold of $\beta = 4$. It illustrates Observation 2 regarding the relationship between network concepts (y-axis) and their eigengene-based analogs (x-axis) in the brain cancer data. Each point corresponds to a module. Figure A: Centralization (y-axis) versus eigengene-based Centralization_E (x-axis); analogous plots for Figure B: Heterogeneity; Figure C: clustering coefficient; Figure D: module significance; and Figure E: hub gene significance. Figure F illustrates the relationship between eigengene significance and hub gene significance. The blue line is the regression line through the points representing proper modules (i.e., the grey, non-module genes are left out). While the red reference line (slope 1, intercept 0) does not always fit well, we observe high squared correlations R^2 between network concepts and their analogs. Since the grey point corresponds to the genes outside properly defined modules, we did not include it in calculations.

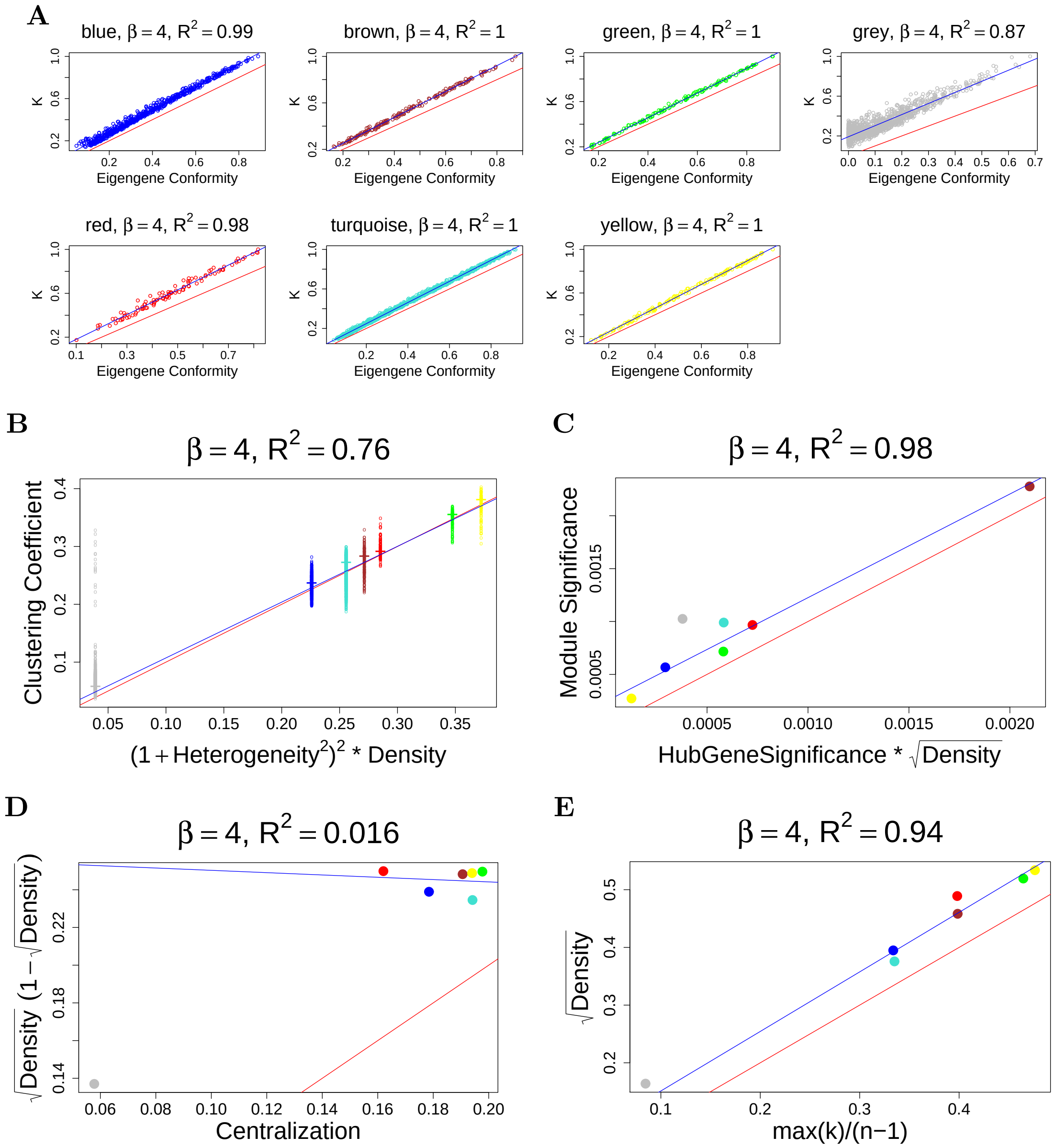

Figure 13: This figure is analogous to Figure 8 in the main article, corresponding to a weighted network constructed with a soft threshold of $\beta = 4$. It illustrates Observation 3 regarding the relationships among network concepts. Figure A illustrates Equation (33) in the main article regarding the relationship between scaled intramodular connectivity $K_i^{(q)}$ (y-axis) and eigengene conformity $a_{e,i}$ (x-axis). Each dot corresponds to a gene colored by its module membership. We find a high squared correlation R^2 even for the grey genes outside properly defined modules. Figure B illustrates Equation (31) regarding the relationship between the clustering coefficient and $(1 + Heterogeneity^2)^2 \times Density$. Again each dot represents a gene. The clustering coefficients of grey genes vary more than those of genes in proper modules. The short horizontal lines correspond to the mean clustering coefficient of each module. Figure C illustrates $ModuleSignif^{(q)} \approx \sqrt{Density^{(q)}} \times HubGeneSignif^{(q)}$ (Equation 37); here each dot corresponds to a module. Since the grey dot corresponds to genes outside of properly defined modules, we have excluded it from the calculation of the squared correlation R^2. Figure D illustrates $Centralization^{(q)} \approx \sqrt{Density^{(q)}}(1 - \sqrt{Density^{(q)}})$ (Equation 40); Figure E illustrates $\frac{k_{max}^{(q)}}{n^{(q)}-1} \approx \sqrt{Density^{(q)}}$ (Equation 38). A reference line (red) with intercept 0 and slope 1 has been added to each plot. The blue line is the regression line through the points representing proper modules (i.e., the grey, non-module genes are left out).

8 Weighted Gene Co-Expression Network Results for $\beta = 5$

Table 6: Values of network concepts for a weighted network constructed with a soft threshold of $\beta = 5$.

Module	blue	brown	green	grey	red	turquoise	yellow
Size ($n^{(q)}$)	606	185	136	1313	105	1112	143
Eigengene Factorizability ($EF(X^{(q)})$)	0.974	0.986	0.991	0.662	0.981	0.978	0.991
$VarExplained(\boldsymbol{E}^{(q)})$	0.591	0.655	0.702	0.278	0.677	0.57	0.71
$max(a_{e,i})$	0.863	0.843	0.883	0.619	0.777	0.898	0.887
$Density$	0.108	0.152	0.206	0.0145	0.178	0.0985	0.221
$Density_E$	0.0934	0.14	0.199	0.0058	0.161	0.0888	0.213
$Centralization$	0.156	0.179	0.191	0.0397	0.152	0.175	0.188
$Centralization_E$	0.171	0.178	0.199	0.0414	0.155	0.179	0.2
$Heterogeneity$	0.54	0.452	0.441	0.525	0.364	0.71	0.451
$Heterogeneity_E$	0.63	0.483	0.461	1.27	0.417	0.778	0.474
$Mean(ClusterCoef)$	0.19	0.22	0.294	0.0505	0.244	0.217	0.319
$ClusterCoef_E$	0.182	0.213	0.29	0.0393	0.219	0.229	0.317
$ModuleSignif$	0.000103	0.000157	0.00121	0.000194	0.000487	0.000469	2.42e-05
$ModuleSignif_E$	2.24e-09	7.56e-06	0.000459	7.35e-11	0.000103	4.91e-05	9.77e-08
$HubGeneSignif$	0.000113	0.000358	0.00226	0.000437	0.000772	0.000881	2.18e-05
$HubGeneSignif_E$	6.33e-09	1.7e-05	0.000912	5.97e-10	0.000201	0.000148	1.89e-07
$EigengeneSignif$	7.33e-09	2.02e-05	0.00103	9.64e-10	0.000258	0.000165	2.13e-07

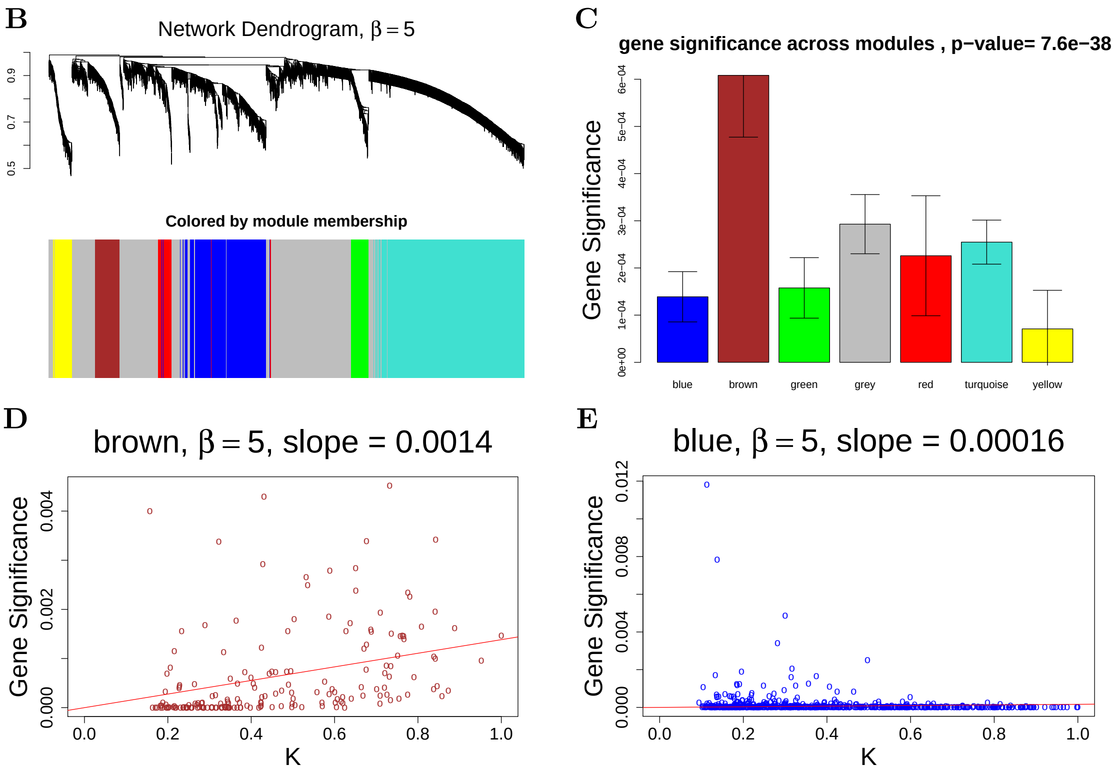

Figure 14: This figure is analogous to Figure 3 in the main article. The only difference is that we use a different dissimilarity for constructing the cluster tree in Figure B. Specifically, we use the topological overlap matrix based dissimilarity corresponding to a weighted network constructed with $\beta = 5$. Figure B depicts the hierarchical cluster tree of genes. Modules correspond to branches of the tree. The branches and module genes are assigned a color as can be seen from the color-bands underneath the tree. Grey denotes genes outside of proper modules. Figure C shows the module significance (average gene significance) of the modules. The underlying gene significance is defined with respect to the patient survival time. Figures D and E show scatter plots of gene significance GS (y-axis) versus scaled connectivity K (x-axis) in the brown and blue module, respectively. The hub gene significance is defined as the slope of the red line, which results from a regression model without an intercept term.

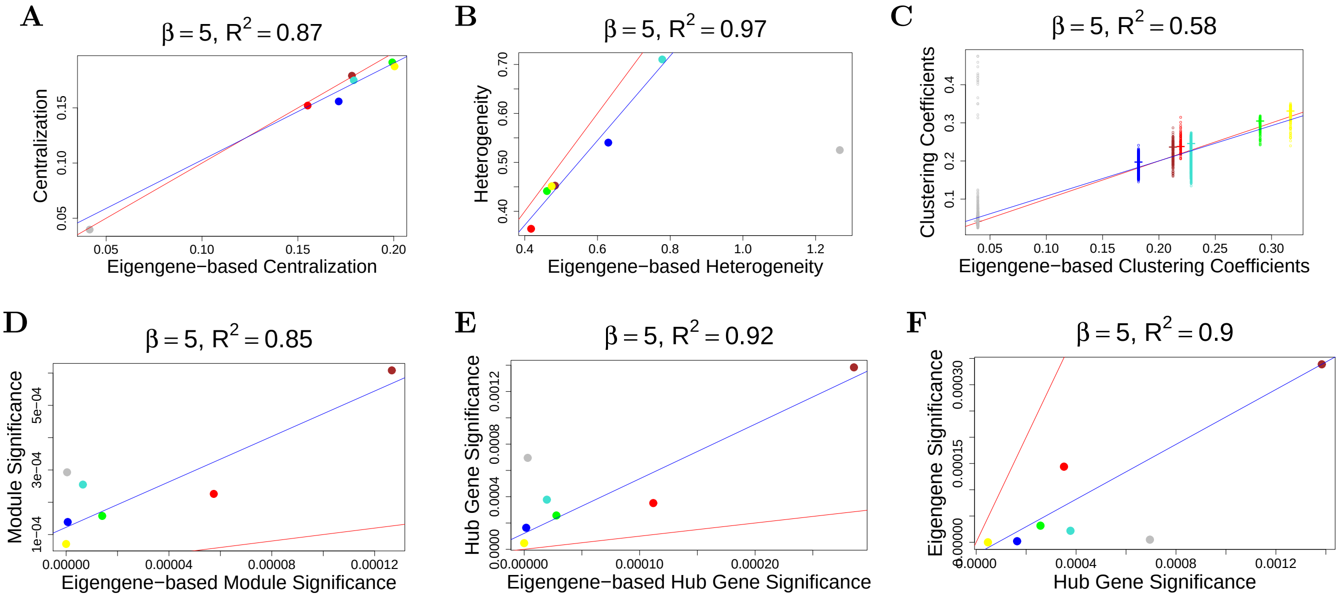

Figure 15: This figure is analogous to Figure 6 in the main article, corresponding to a weighted network constructed with a soft threshold of $\beta = 5$. It illustrates Observation 2 regarding the relationship between network concepts (y-axis) and their eigengene-based analogs (x-axis) in the brain cancer data. Each point corresponds to a module. Figure A: Centralization (y-axis) versus eigengene-based Centralization$_E$ (x-axis); analogous plots for Figure B: Heterogeneity; Figure C: clustering coefficient; Figure D: module significance; and Figure E: hub gene significance. Figure F illustrates the relationship between eigengene significance and hub gene significance. The blue line is the regression line through the points representing proper modules (i.e., the grey, non-module genes are left out). While the red reference line (slope 1, intercept 0) does not always fit well, we observe high squared correlations R^2 between network concepts and their analogs. Since the grey point corresponds to the genes outside properly defined modules, we did not include it in calculations.

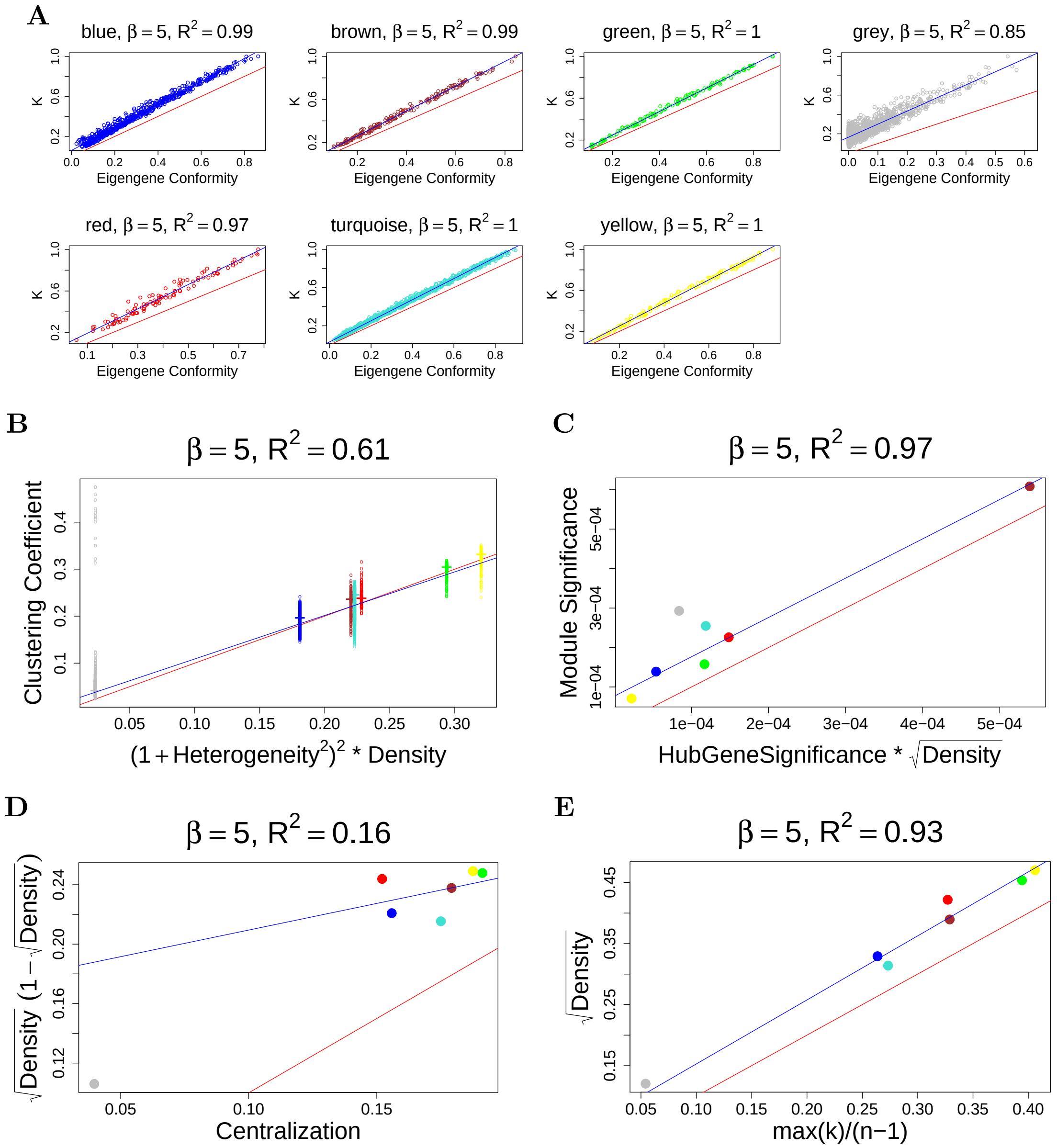

Figure 16: This figure is analogous to Figure 8 in the main article, corresponding to a weighted network constructed with a soft threshold of $\beta = 5$. It illustrates Observation 3 regarding the relationships among network concepts. Figure A illustrates Equation (33) in the main article regarding the relationship between scaled intramodular connectivity $K_i^{(q)}$ (y-axis) and eigengene conformity $a_{e,i}$ (x-axis). Each dot corresponds to a gene colored by its module membership. We find a high squared correlation R^2 even for the grey genes outside properly defined modules. Figure B illustrates Equation (31) regarding the relationship between the clustering coefficient and $(1 + Heterogeneity^2)^2 \times Density$. Again each dot represents a gene. The clustering coefficients of grey genes vary more than those of genes in proper modules. The short horizontal lines correspond to the mean clustering coefficient of each module. Figure C illustrates $ModuleSignif^{(q)} \approx \sqrt{Density^{(q)}} \times HubGeneSignif^{(q)}$ (Equation 37); here each dot corresponds to a module. Since the grey dot corresponds to genes outside of properly defined modules, we have excluded it from the calculation of the squared correlation R^2. Figure D illustrates $Centralization^{(q)} \approx \sqrt{Density^{(q)}}(1 - \sqrt{Density^{(q)}})$ (Equation 40); Figure E illustrates $\frac{k_{max}^{(q)}}{n^{(q)}-1} \approx \sqrt{Density^{(q)}}$ (Equation 38). A reference line (red) with intercept 0 and slope 1 has been added to each plot. The blue line is the regression line through the points representing proper modules (i.e., the grey, non-module genes are left out).

9 Weighted Gene Co-Expression Network Results for $\beta = 6$

Table 7: Values of network concepts for a weighted network constructed with a soft threshold of $\beta = 6$.

Module	blue	brown	green	grey	red	turquoise	yellow
Size ($n^{(q)}$)	606	185	136	1313	105	1112	143
Eigengene Factorizability ($EF(X^{(q)})$)	0.974	0.986	0.991	0.662	0.981	0.978	0.991
$VarExplained(\boldsymbol{E}^{(q)})$	0.591	0.655	0.702	0.278	0.677	0.57	0.71
$max(a_{e,i})$	0.838	0.814	0.861	0.562	0.738	0.878	0.865
$Density$	0.0775	0.112	0.16	0.00829	0.135	0.0713	0.175
$Density_E$	0.0637	0.1	0.151	0.0028	0.116	0.0626	0.165
$Centralization$	0.137	0.165	0.181	0.0279	0.139	0.157	0.177
$Centralization_E$	0.149	0.16	0.188	0.027	0.139	0.158	0.19
$Heterogeneity$	0.624	0.532	0.51	0.593	0.422	0.826	0.518
$Heterogeneity_E$	0.74	0.572	0.537	1.47	0.494	0.905	0.549
$Mean(ClusterCoef)$	0.159	0.183	0.254	0.0405	0.207	0.191	0.278
$ClusterCoef_E$	0.152	0.175	0.249	0.0279	0.178	0.207	0.277
$ModuleSignif$	2.92e-05	3.58e-05	0.000377	5.38e-05	0.000151	0.000139	4.84e-06
$ModuleSignif_E$	4.36e-11	7.34e-07	0.000101	8.03e-13	1.68e-05	7.22e-06	3.98e-09
$HubGeneSignif$	2.73e-05	9.07e-05	0.000746	0.000122	0.000231	0.000257	3.8e-06
$HubGeneSignif_E$	1.45e-10	1.9e-06	0.000225	8.53e-12	3.65e-05	2.54e-05	8.52e-09
$EigengeneSignif$	1.73e-10	2.33e-06	0.000261	1.52e-11	4.95e-05	2.89e-05	9.84e-09

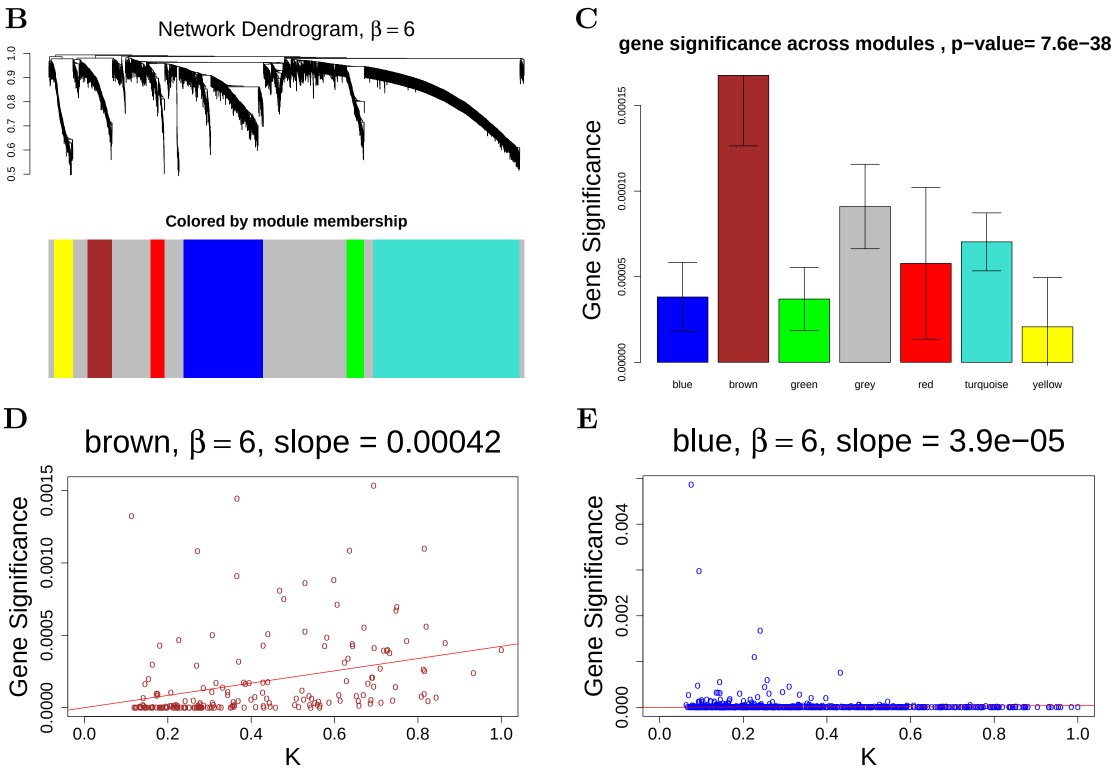

Figure 17: This figure is analogous to Figure 3 in the main article. The only difference is that we use a different dissimilarity for constructing the cluster tree in Figure B. Specifically, we use the topological overlap matrix based dissimilarity corresponding to a weighted network constructed with $\beta = 6$. Figure B depicts the hierarchical cluster tree of genes. Modules correspond to branches of the tree. The branches and module genes are assigned a color as can be seen from the color-bands underneath the tree. Grey denotes genes outside of proper modules. Figure C shows the module significance (average gene significance) of the modules. The underlying gene significance is defined with respect to the patient survival time. Figures D and E show scatter plots of gene significance GS (y-axis) versus scaled connectivity K (x-axis) in the brown and blue module, respectively. The hub gene significance is defined as the slope of the red line, which results from a regression model without an intercept term.

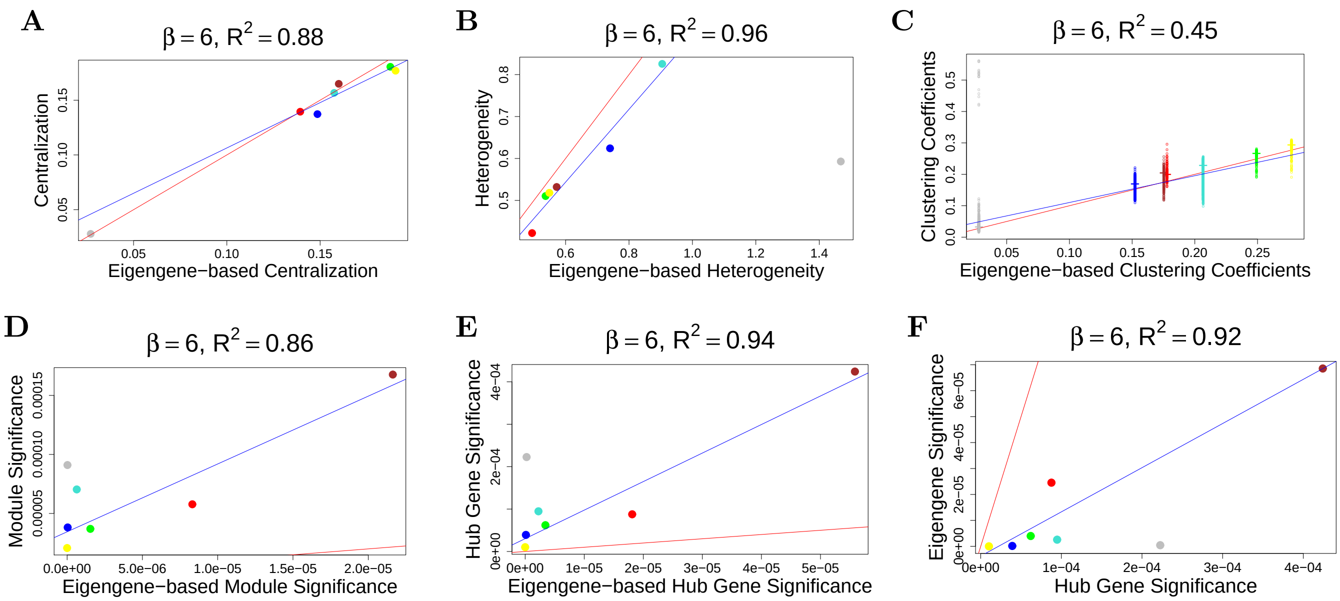

Figure 18: This figure is analogous to Figure 6 in the main article, corresponding to a weighted network constructed with a soft threshold of $\beta = 6$. It illustrates Observation 2 regarding the relationship between network concepts (y-axis) and their eigengene-based analogs (x-axis) in the brain cancer data. Each point corresponds to a module. Figure A: Centralization (y-axis) versus eigengene-based Centralization$_E$ (x-axis); analogous plots for Figure B: Heterogeneity; Figure C: clustering coefficient; Figure D: module significance; and Figure E: hub gene significance. Figure F illustrates the relationship between eigengene significance and hub gene significance. The blue line is the regression line through the points representing proper modules (i.e., the grey, non-module genes are left out). While the red reference line (slope 1, intercept 0) does not always fit well, we observe high squared correlations R^2 between network concepts and their analogs. Since the grey point corresponds to the genes outside properly defined modules, we did not include it in calculations.

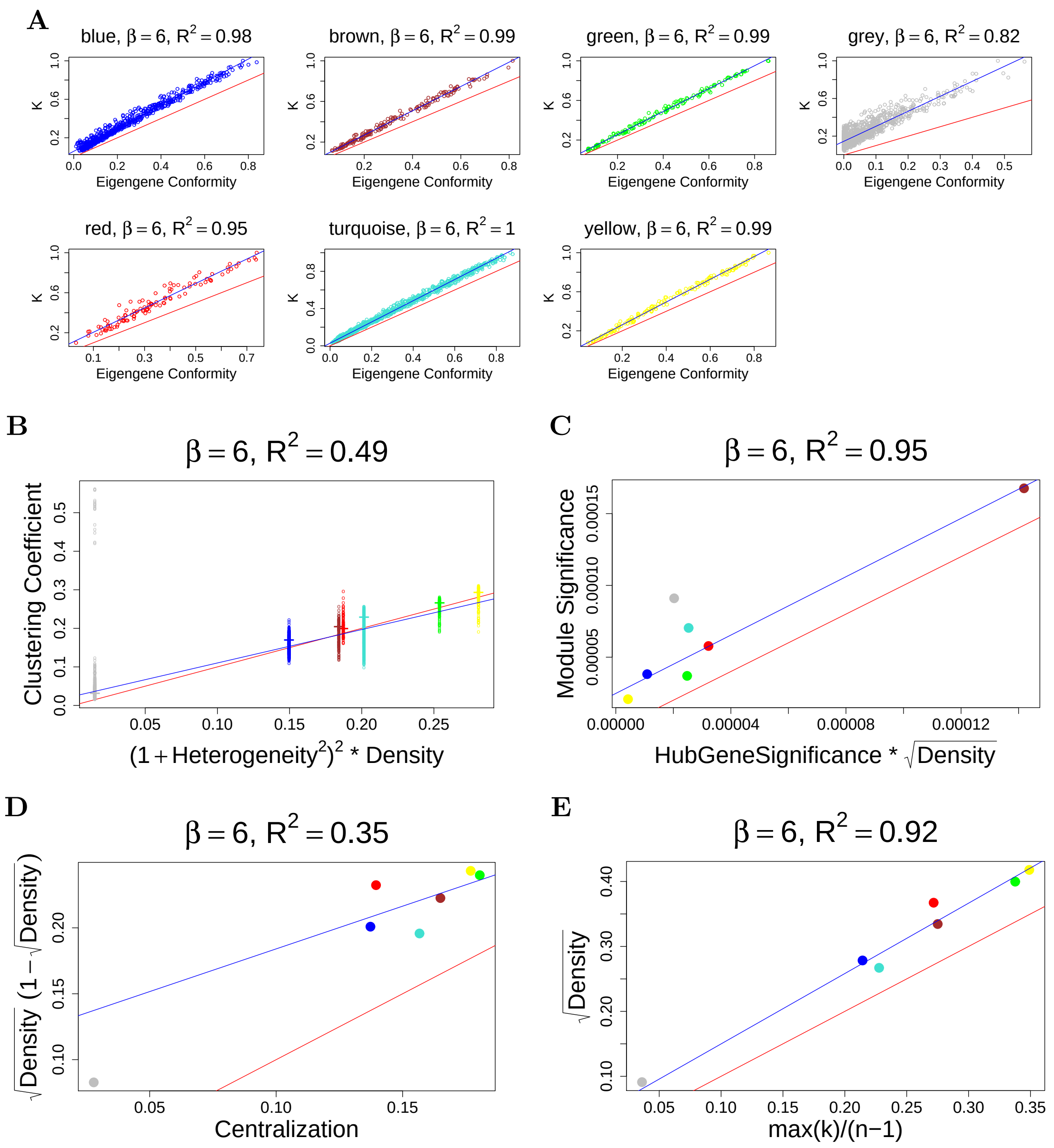

Figure 19: This figure is analogous to Figure 8 in the main article, corresponding to a weighted network constructed with a soft threshold of $\beta = 6$. It illustrates Observation 3 regarding the relationships among network concepts. Figure A illustrates Equation (33) in the main article regarding the relationship between scaled intramodular connectivity $K_i^{(q)}$ (y-axis) and eigengene conformity $a_{e,i}$ (x-axis). Each dot corresponds to a gene colored by its module membership. We find a high squared correlation R^2 even for the grey genes outside properly defined modules. Figure B illustrates Equation (31) regarding the relationship between the clustering coefficient and $(1 + Heterogeneity^2)^2 \times Density$. Again each dot represents a gene. The clustering coefficients of grey genes vary more than those of genes in proper modules. The short horizontal lines correspond to the mean clustering coefficient of each module. Figure C illustrates $ModuleSignif^{(q)} \approx \sqrt{Density^{(q)}} \times HubGeneSignif^{(q)}$ (Equation 37); here each dot corresponds to a module. Since the grey dot corresponds to genes outside of properly defined modules, we have excluded it from the calculation of the squared correlation R^2. Figure D illustrates $Centralization^{(q)} \approx \sqrt{Density^{(q)}}(1 - \sqrt{Density^{(q)}})$ (Equation 40); Figure E illustrates $\frac{k_{max}^{(q)}}{n^{(q)}-1} \approx \sqrt{Density^{(q)}}$ (Equation 38). A reference line (red) with intercept 0 and slope 1 has been added to each plot. The blue line is the regression line through the points representing proper modules (i.e., the grey, non-module genes are left out).

10 Unweighted Gene Co-Expression Network Results for $\tau = 0.7$

Table 8: Values of network concepts for an unweighted network constructed with a hard threshold of $\tau = 0.7$.

Module	blue	brown	green	grey	red	turquoise	yellow
Size ($n^{(q)}$)	606	185	136	1313	105	1112	143
Eigengene Factorizability ($EF(X^{(q)})$)	0.974	0.986	0.991	0.662	0.981	0.978	0.991
$VarExplained(\boldsymbol{E}^{(q)})$	0.591	0.655	0.702	0.278	0.677	0.57	0.71
$max(a_{e,i})$	0.971	0.966	0.975	0.908	0.951	0.979	0.976
$Density$	0.214	0.328	0.49	0.0084	0.425	0.177	0.51
$Density_E$	0.581	0.652	0.699	0.233	0.678	0.554	0.706
$Centralization$	0.428	0.46	0.39	0.084	0.43	0.355	0.369
$Centralization_E$	0.16	0.132	0.121	0.206	0.111	0.175	0.119
$Heterogeneity$	0.796	0.649	0.514	1.23	0.45	1.01	0.51
$Heterogeneity_E$	0.138	0.101	0.105	0.44	0.0906	0.171	0.111
$Mean(ClusterCoef)$	0.687	0.724	0.792	0.308	0.733	0.674	0.818
$ClusterCoef_E$	0.603	0.662	0.71	0.331	0.683	0.587	0.718
$ModuleSignif$	0.088	0.117	0.21	0.105	0.158	0.142	0.0652
$HubGeneSignif$	0.132	0.231	0.331	0.395	0.279	0.241	0.0814
$EigengeneSignif$	0.0236	0.115	0.253	0.0157	0.192	0.175	0.0463

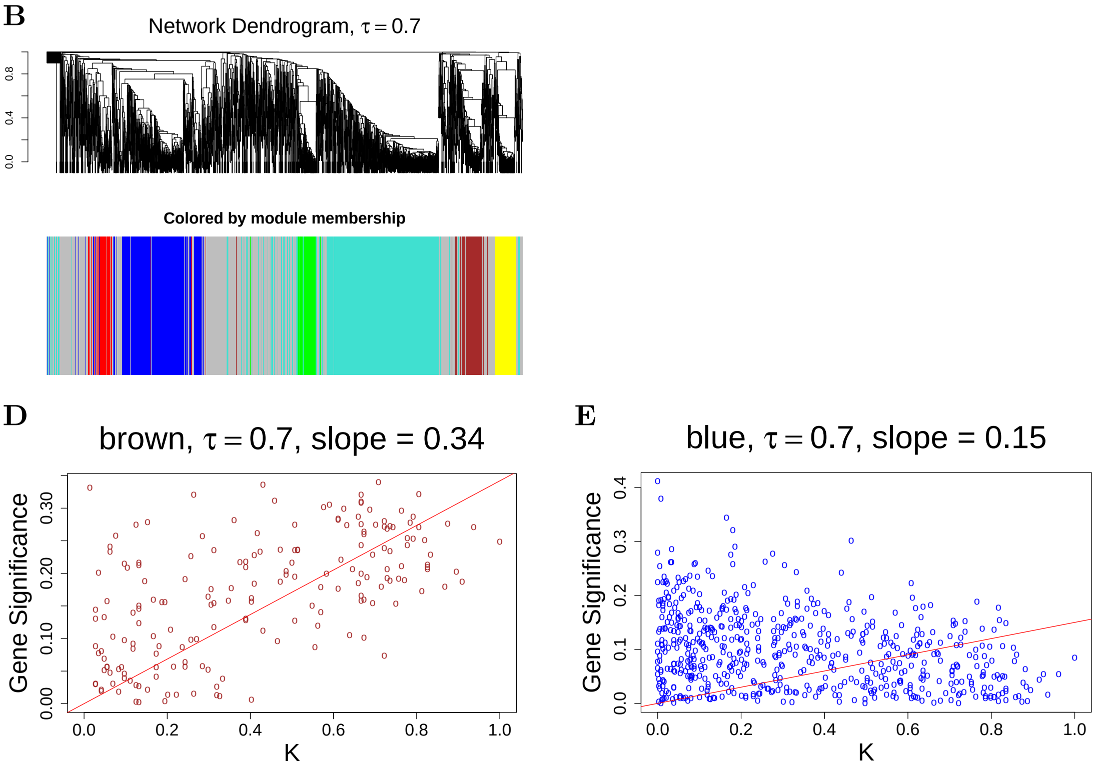

Figure 20: This figure is analogous to Figure 3 in the main article. The only difference is that we use a different dissimilarity for constructing the cluster tree in Figure B. Specifically, we use the topological overlap matrix based dissimilarity corresponding to an unweighted network constructed with $\tau = 0.7$. Figure B depicts the hierarchical cluster tree of genes. Modules correspond to branches of the tree. The branches and module genes are assigned a color as can be seen from the color-bands underneath the tree. Grey denotes genes outside of proper modules. Figure C would be identical to the case of $\beta = 1$, and thus is omitted here. Figures D and E show scatter plots of gene significance GS (y-axis) versus scaled connectivity K (x-axis) in the brown and blue module, respectively. The underlying gene significance is defined with respect to the patient survival time. The hub gene significance is defined as the slope of the red line, which results from a regression model without an intercept term.

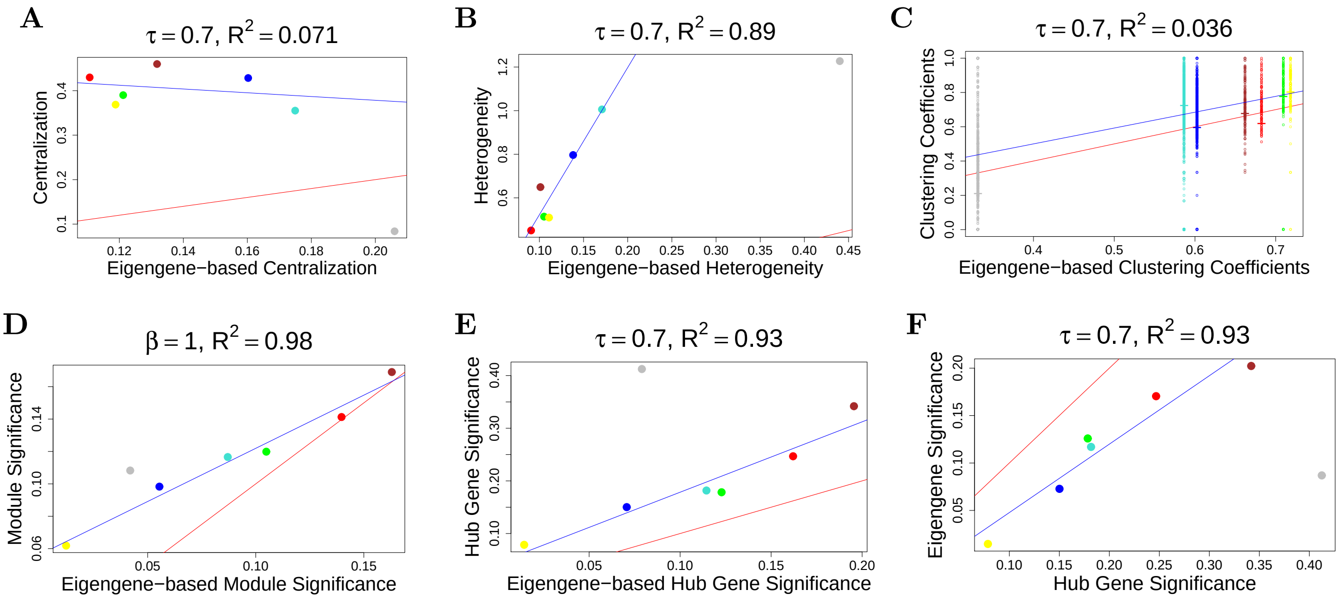

Figure 21: This figure is analogous to Figure 6 in the main article, corresponding to an unweighted network constructed with a hard threshold of $\tau = 0.7$. It illustrates Observation 2 regarding the relationship between network concepts (y-axis) and their eigengene-based analogs (x-axis) in the brain cancer data. Each point corresponds to a module. Figure A: Centralization (y-axis) versus eigengene-based Centralization$_E$ (x-axis); analogous plots for Figure B: Heterogeneity; Figure C: clustering coefficient; Figure D: module significance; and Figure E: hub gene significance. Figure D is identical to the case of $\beta = 1$. Figure F illustrates the relationship between eigengene significance and hub gene significance. The blue line is the regression line through the points representing proper modules (i.e., the grey, non-module genes are left out). While the red reference line (slope 1, intercept 0) does not always fit well, we observe high squared correlations R^2 between network concepts and their analogs. Since the grey point corresponds to the genes outside properly defined modules, we did not include it in calculations.

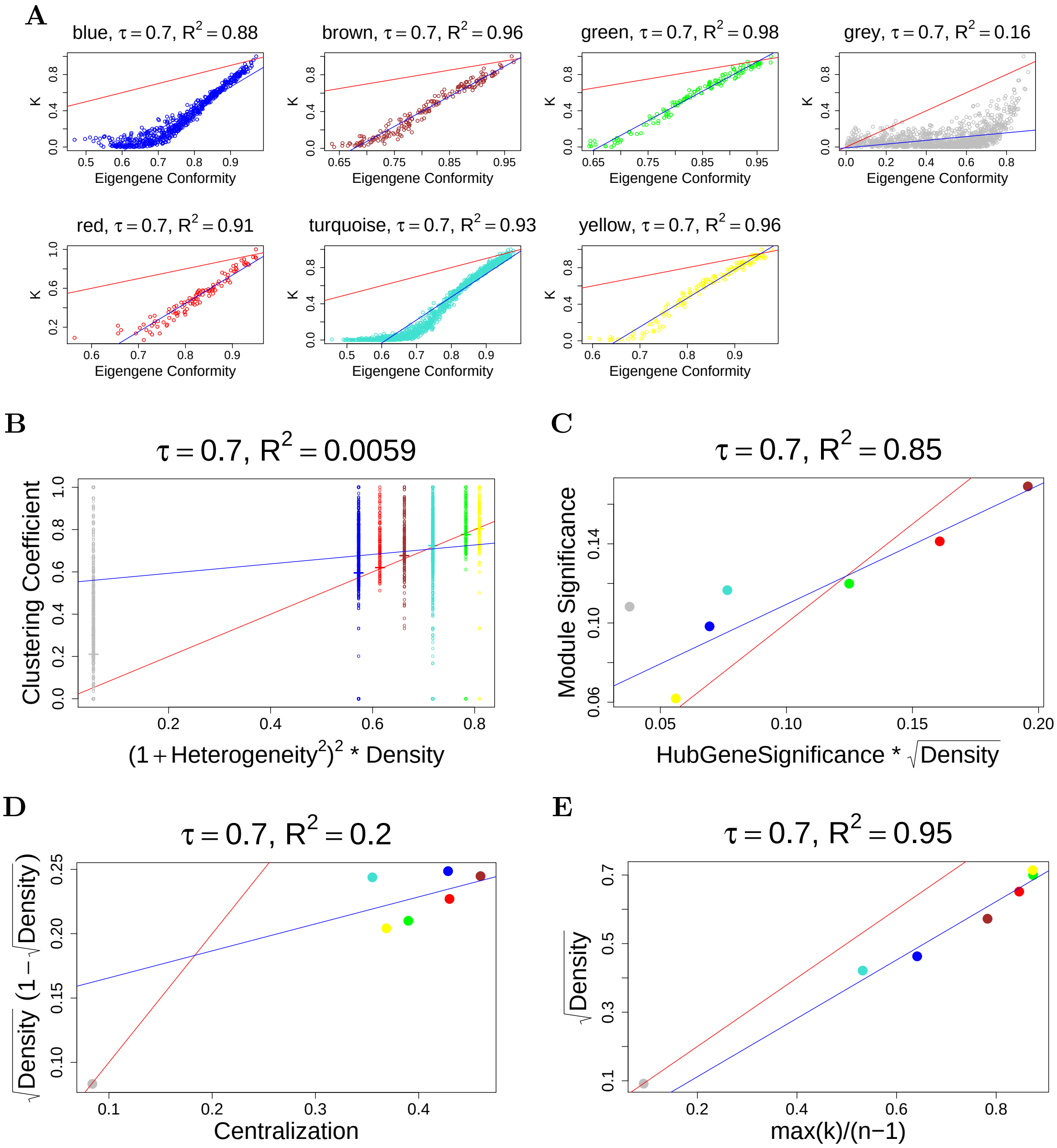

Figure 22: This figure is analogous to Figure 8 in the main article, corresponding to an unweighted network constructed with a hard threshold of $\tau = 0.7$. It illustrates Observation 3 regarding the relationships among network concepts. Figure A illustrates Equation (33) in the main article regarding the relationship between scaled intramodular connectivity $K_i^{(q)}$ (y-axis) and eigengene conformity $a_{e,i}$ (x-axis). Each dot corresponds to a gene colored by its module membership. We find a high squared correlation R^2 even for the grey genes outside properly defined modules. Figure B illustrates Equation (31) regarding the relationship between the clustering coefficient and $(1 + Heterogeneity^2)^2 \times Density$. Again each dot represents a gene. The clustering coefficients of grey genes vary more than those of genes in proper modules. The short horizontal lines correspond to the mean clustering coefficient of each module. Figure C illustrates $ModuleSignif^{(q)} \approx \sqrt{Density^{(q)}} \times HubGeneSignif^{(q)}$ (Equation 37); here each dot corresponds to a module. Since the grey dot corresponds to genes outside of properly defined modules, we have excluded it from the calculation of the squared correlation R^2. Figure D illustrates $Centralization^{(q)} \approx \sqrt{Density^{(q)}}(1 - \sqrt{Density^{(q)}})$ (Equation 40); Figure E illustrates $\frac{k_{max}^{(q)}}{n^{(q)}-1} \approx \sqrt{Density^{(q)}}$ (Equation 38). A reference line (red) with intercept 0 and slope 1 has been added to each plot. The blue line is the regression line through the points representing proper modules (i.e., the grey, non-module genes are left out).

11 Unweighted Gene Co-Expression Network Results for $\tau = 0.5$

Table 9: Values of network concepts for an unweighted network constructed with a hard threshold of $\tau = 0.5$.

Module	blue	brown	green	grey	red	turquoise	yellow
Size ($n^{(q)}$)	606	185	136	1313	105	1112	143
Eigengene Factorizability ($EF(X^{(q)})$)	0.974	0.986	0.991	0.662	0.981	0.978	0.991
$VarExplained(\boldsymbol{E}^{(q)})$	0.591	0.655	0.702	0.278	0.677	0.57	0.71
$max(a_{e,i})$	0.971	0.966	0.975	0.908	0.951	0.979	0.976
$Density$	0.716	0.889	0.941	0.136	0.901	0.642	0.932
$Density_E$	0.581	0.652	0.699	0.233	0.678	0.554	0.706
$Centralization$	0.278	0.112	0.0599	0.319	0.101	0.35	0.0689
$Centralization_E$	0.16	0.132	0.121	0.206	0.111	0.175	0.119
$Heterogeneity$	0.262	0.117	0.0843	0.567	0.126	0.342	0.0944
$Heterogeneity_E$	0.138	0.101	0.105	0.44	0.0906	0.171	0.111
$Mean(ClusterCoef)$	0.84	0.917	0.954	0.497	0.933	0.824	0.95
$ClusterCoef_E$	0.603	0.662	0.71	0.331	0.683	0.587	0.718
$ModuleSignif$	0.088	0.117	0.21	0.105	0.158	0.142	0.0652
$HubGeneSignif$	0.107	0.132	0.225	0.248	0.174	0.203	0.068
$EigengeneSignif$	0.0236	0.115	0.253	0.0157	0.192	0.175	0.0463

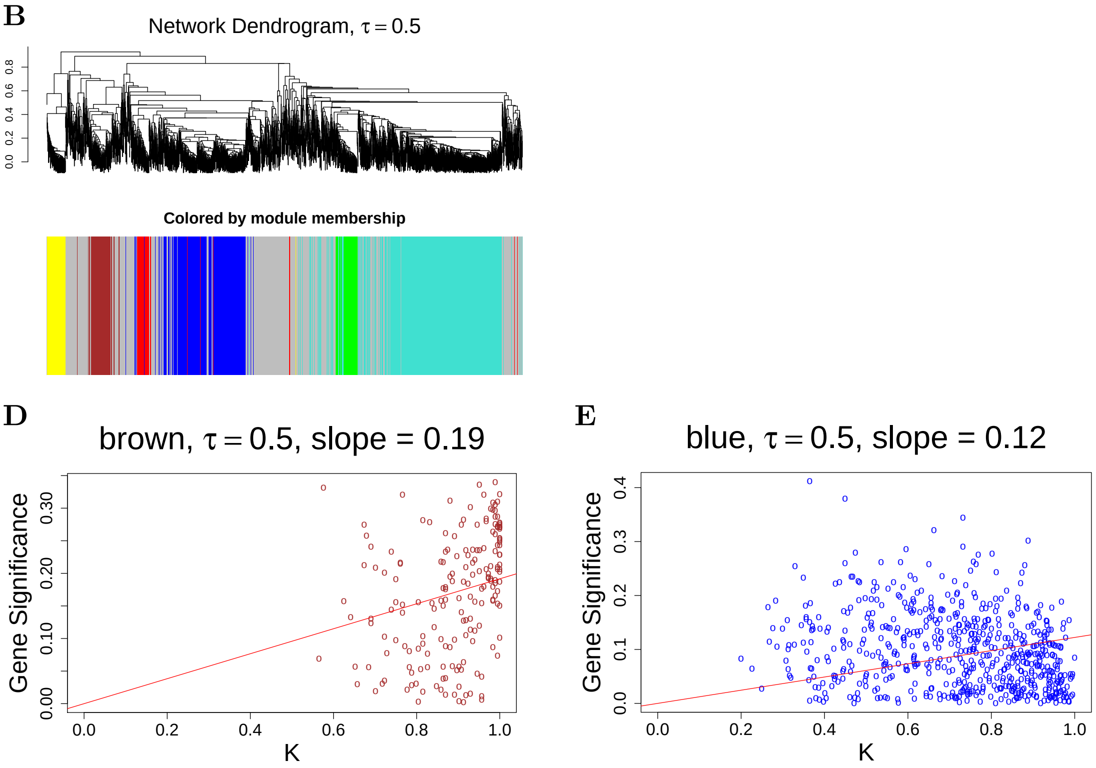

Figure 23: This figure is analogous to Figure 3 in the main article. The only difference is that we use a different dissimilarity for constructing the cluster tree in Figure B. Specifically, we use the topological overlap matrix based dissimilarity corresponding to an unweighted network constructed with $\tau = 0.5$. Figure B depicts the hierarchical cluster tree of genes. Modules correspond to branches of the tree. The branches and module genes are assigned a color as can be seen from the color-bands underneath the tree. Grey denotes genes outside of proper modules. Figure C would be identical to the case of $\beta = 1$, and thus is omitted here. Figures D and E show scatter plots of gene significance GS (y-axis) versus scaled connectivity K (x-axis) in the brown and blue module, respectively. The underlying gene significance is defined with respect to the patient survival time. The hub gene significance is defined as the slope of the red line, which results from a regression model without an intercept term.

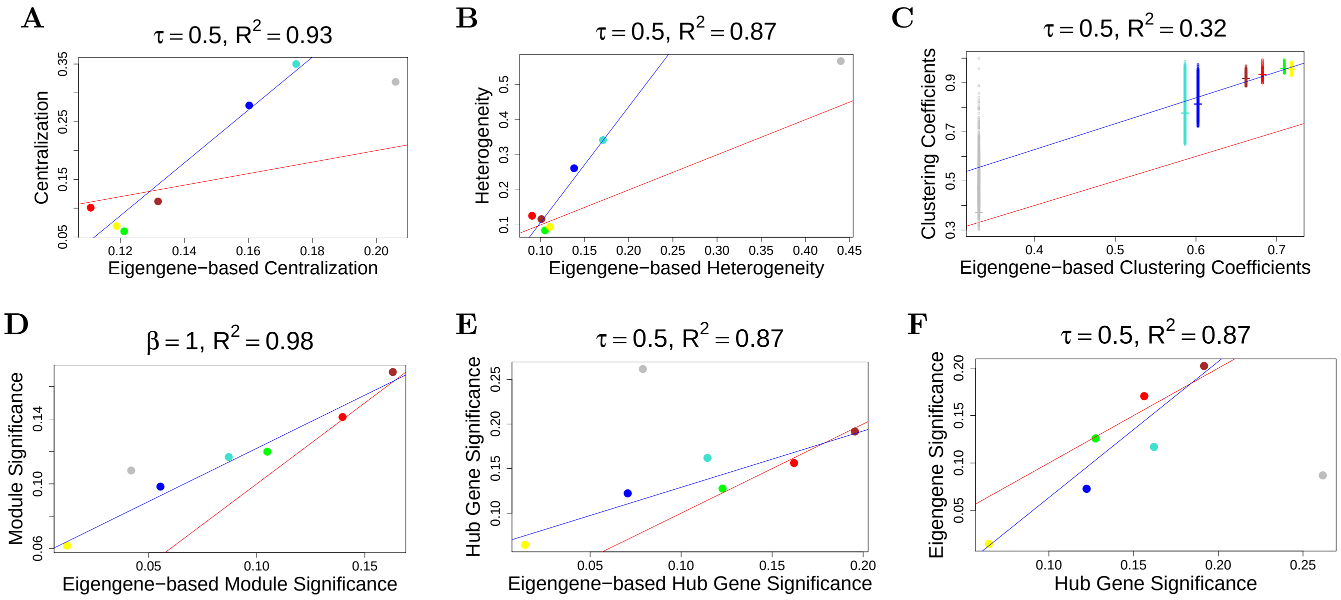

Figure 24: This figure is analogous to Figure 6 in the main article, corresponding to an unweighted network constructed with a hard threshold of $\tau = 0.5$. It illustrates Observation 2 regarding the relationship between network concepts (y-axis) and their eigengene-based analogs (x-axis) in the brain cancer data. Each point corresponds to a module. Figure A: Centralization (y-axis) versus eigengene-based Centralization$_E$ (x-axis); analogous plots for Figure B: Heterogeneity; Figure C: clustering coefficient; Figure D: module significance; and Figure E: hub gene significance. Figure D is identical to the case of $\beta = 1$. Figure F illustrates the relationship between eigengene significance and hub gene significance. The blue line is the regression line through the points representing proper modules (i.e., the grey, non-module genes are left out). While the red reference line (slope 1, intercept 0) does not always fit well, we observe high squared correlations R^2 between network concepts and their analogs. Since the grey point corresponds to the genes outside properly defined modules, we did not include it in calculations.

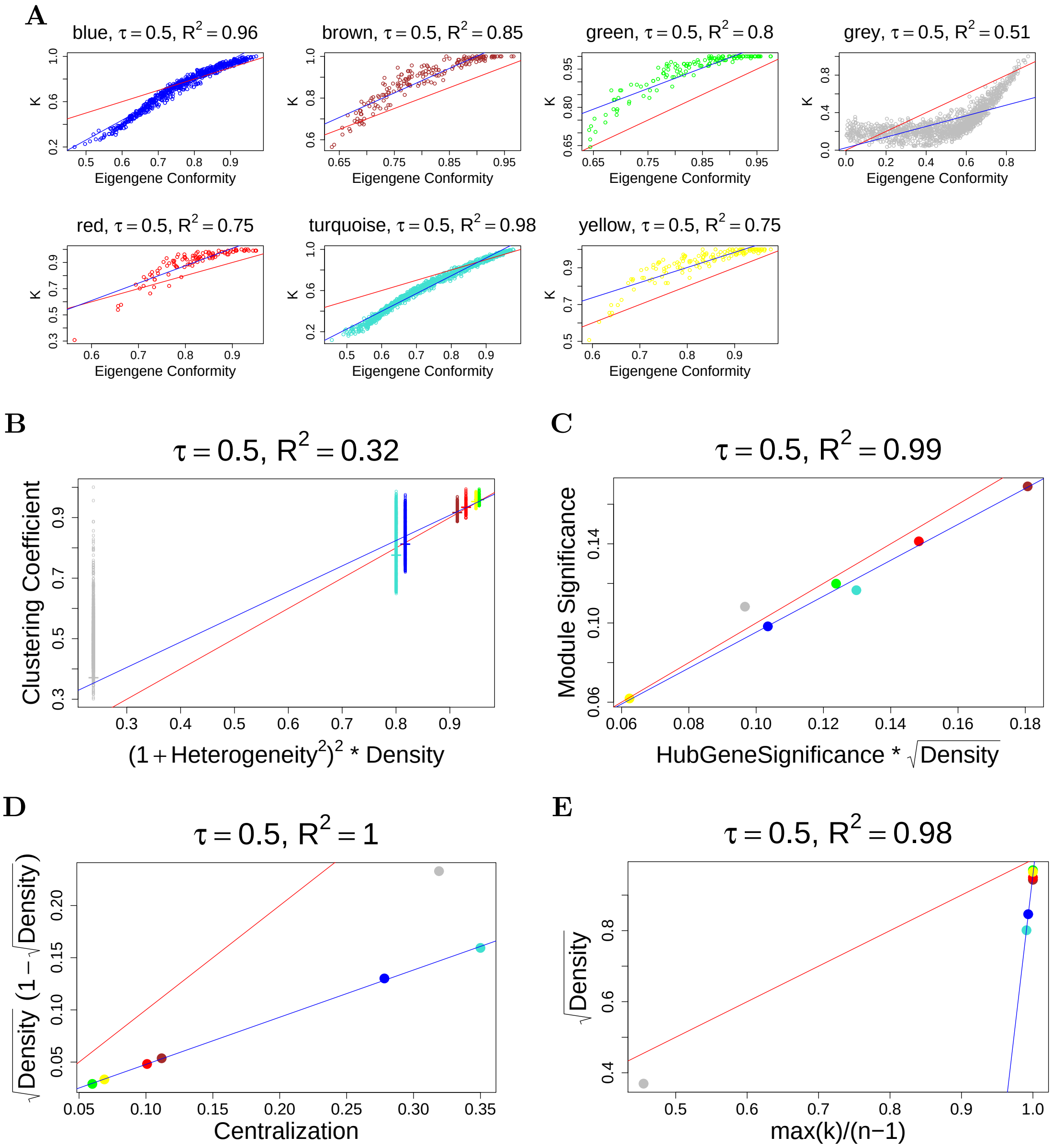

Figure 25: This figure is analogous to Figure 8 in the main article, corresponding to an unweighted network constructed with a hard threshold of $\tau = 0.5$. It illustrates Observation 3 regarding the relationships among network concepts. Figure A illustrates Equation (33) in the main article regarding the relationship between scaled intramodular connectivity $K_i^{(q)}$ (y-axis) and eigengene conformity $a_{e,i}$ (x-axis). Each dot corresponds to a gene colored by its module membership. We find a high squared correlation R^2 even for the grey genes outside properly defined modules. Figure B illustrates Equation (31) regarding the relationship between the clustering coefficient and $(1 + Heterogeneity^2)^2 \times Density$. Again each dot represents a gene. The clustering coefficients of grey genes vary more than those of genes in proper modules. The short horizontal lines correspond to the mean clustering coefficient of each module. Figure C illustrates $ModuleSignif^{(q)} \approx \sqrt{Density^{(q)}} \times HubGeneSignif^{(q)}$ (Equation 37); here each dot corresponds to a module. Since the grey dot corresponds to genes outside of properly defined modules, we have excluded it from the calculation of the squared correlation R^2. Figure D illustrates $Centralization^{(q)} \approx \sqrt{Density^{(q)}}(1 - \sqrt{Density^{(q)}})$ (Equation 40); Figure E illustrates $\frac{k_{max}^{(q)}}{n^{(q)}-1} \approx \sqrt{Density^{(q)}}$ (Equation 38). A reference line (red) with intercept 0 and slope 1 has been added to each plot. The blue line is the regression line through the points representing proper modules (i.e., the grey, non-module genes are left out).

References

Horvath, S., Zhang, B., Carlson, M., Lu, K.V., Zhu, S., Felciano, R.M., Laurance, M.F., Zhao, W., Shu, Q., Lee, Y., Scheck, A.C., Liau, L.M., Wu, H., Geschwind, D.H., Febbo, P.G., Kornblum, H.I., T.F., Cloughesy, Nelson, S.F. and Mischel, P.S. (2006). Analysis of oncogenic signaling networks in glioblastoma identifies aspm as a novel molecular target. *PNAS*, **103**(46), 17402–7.
