# Supplementary material for: Geometric Interpretation of Gene Coexpression Network Analysis
Source: PLoS Comput Biol. 2008 Aug 15;4(8):e1000117. doi: 10.1371/journal.pcbi.1000117 (PMC2446438; doi:10.1371/journal.pcbi.1000117)
Supplement: Text S3 — Robustness Analysis of the Yeast Gene Coexpression Network. This supporting text provides a detailed analysis of the yeast cell cycle gene coexpression network. The robustness analysis illustrates how the results change with regard to different network construction methods. (2.62 MB PDF) [file pcbi.1000117.s003.pdf]

Text S3: Robustness Analysis of the Yeast Gene Co-expression Networks

Jun Dong and Steve Horvath*

Dept. of Human Genetics, David Geffen School of Medicine, UCLA
Dept. of Biostatistics, School of Public Health, UCLA
*Correspondence: shorvath@mednet.ucla.edu

Abstract

This is a supplement of the article "Geometric Interpretation of Gene Co-Expression Network Analysis". Here we illustrate our theoretical results using gene expression data from the yeast application. In particular, we study the robustness of our theoretical findings with regard to alternative methods of constructing a network. We describe the results for weighted co-expression networks constructed using different soft-thresholds $\beta \geq 1$ in

$$a_{ij} = |cor(\boldsymbol{x}_i, \boldsymbol{x}_j)|^{\beta}.$$

Further, we report the analogous findings for unweighted networks constructed on the basis of

$$a_{ij} = Ind(|cor(\boldsymbol{x}_i, \boldsymbol{x}_j)| \geq \tau),$$

where τ is the 'hard' threshold parameter, and $Ind(\cdot)$ is the indicator function taking value of 1 if the condition is true, and 0 otherwise. We provide empirical evidence that co-expression modules tend to have high eigengene factorizability and that the maximum conformity assumption is satisfied for low powers of β. Our robustness analysis shows that many of our theoretical results apply even if our underlying assumptions are not satisfied.

1 Yeast Gene Co-expression Network Application

In this supplement, we illustrate our theoretical derivations using a yeast gene co-expression network. The yeast microarray data were derived from experiments designed to test the Cell Cycle (Spellman *et al.*, 1998). A detailed biological description of the modules and the importance of intramodular connectivity can be found in (Carlson *et al.*, 2006). In this supplement and in Figure 9 of the main article, we focus on the relationships between the network concepts and a gene significance measure that encodes knock out essentiality, i.e. $GS_i = 1$ if the i-th gene is known to be essential and 0 otherwise. In contrast to the other applications, this gene significance measure is not based on a sample trait. Our theoretical derivations for relating module significance to hub gene significance assumed a sample trait based gene significance measure. Although this important assumption is violated for knock-out essentiality, it is striking that the relationship between hub gene significance and module significance can still be observed empirically (Figure 9 of the main article).

We have constructed weighted networks with $\beta = 1, 2, 3, 4, 5, 6$ and 7, and unweighted networks with $\tau = 0.65$ and 0.5. For the unweighted networks, we use the eigengene-based network concepts of weighted networks with $\beta = 1$ for demonstration purposes. Since the gene significance measure in this application is not based on a sample trait, several network concepts are not available, including the eigengene significance and all the eigengene-based significance measures. Therefore the panels (D,E,F) in the second figure of each section are also not available.

2 Robustness of Module Definition

Our module definition was based on the topological overlap measure (TOM) in conjunction with average linkage hierarchical clustering (refer to the Methods Supplement). In this application, we used TOM of an weighted network with power $\beta = 7$. To facilitate a comparison, we used this network module assignment for the other weighted and unweighted network analysis as well. In each of the figure below, the upper panel is the dendrogram of the average linkage hierarchical clustering method using the specific network construction parameter, and the lower panel shows genes colored by their module membership. As the figures show, our module definition is quite robust with regard to the choice of network construction methods.

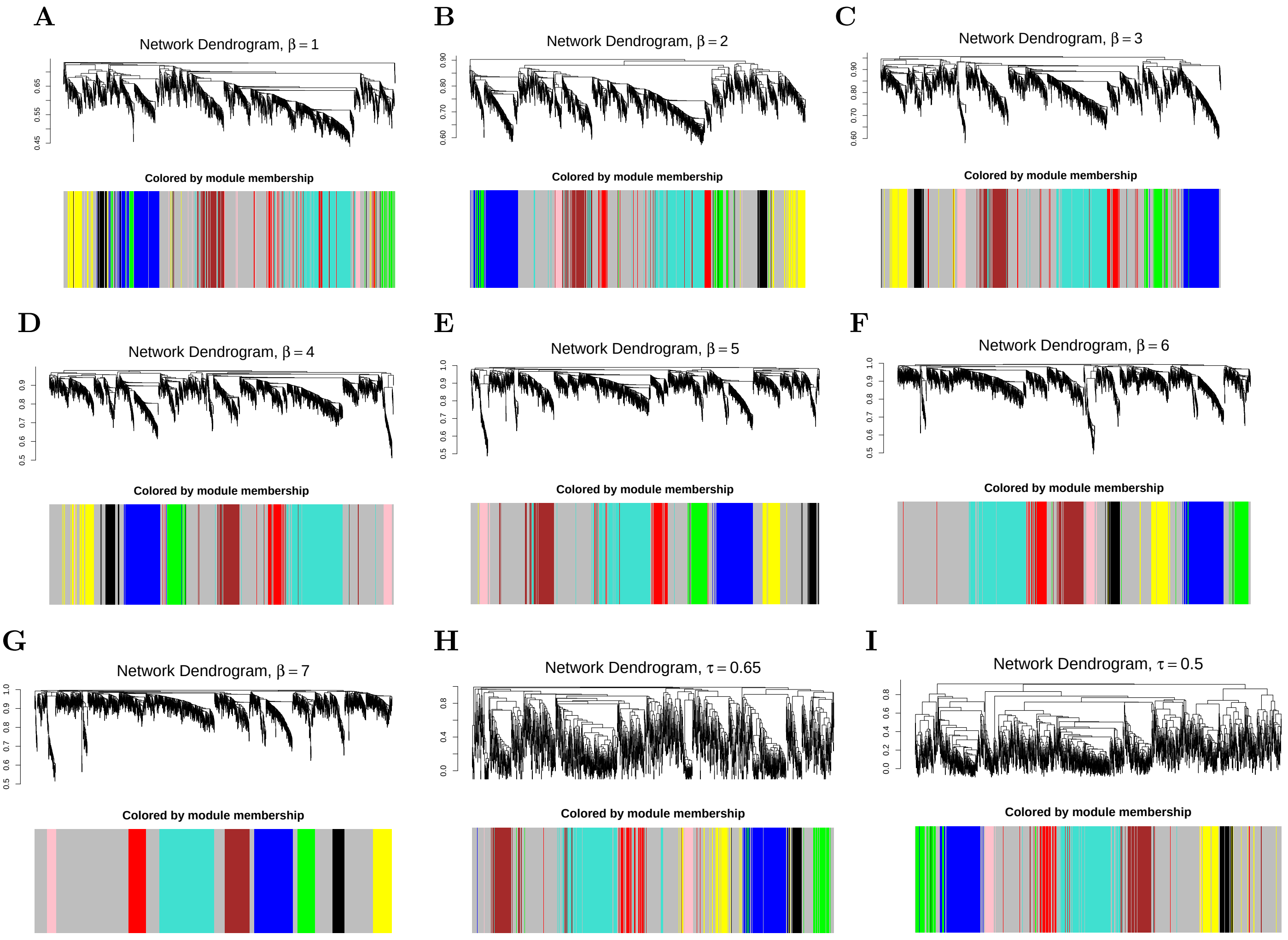

Figure 1: Robustness of module definition. In each figure, genes are colored by their module membership.

3 Summary of Robustness Analysis

To make this supplement self-contained, we repeat the following summary table from our main article. In the rest of this supplement, we provide the details on how we arrived at the R^2 values of this table.

Table 1: Robustness Analysis of the Yeast Co-expression Network. The table reports how the relation between network concepts changes as function of different soft threshold parameters β or hard thresholds used in the network construction. For each relationship and each network construction method, the table entry reports the squared correlation R^2 across the proper modules. For within module comparisons the table reports median R^2 values.

Squared Correlation R^2 Across Modules.	Weighted Networks Soft Threshold β							Unweighted Net Hard Threshold τ	
Relation	1	2	3	4	5	6	7	0.65	0.5
$Centralization \approx Centralization_E$	0.99	0.97	0.97	0.98	0.98	0.98	0.98	0.53	0.60
$Heterogeneity \approx Heterogeneity_E$	0.86	0.92	0.94	0.94	0.93	0.92	0.91	0.13	0.006
$ClusterCoef_i \approx ClusterCoef_E$	0.98	0.97	0.94	0.92	0.89	0.86	0.82	0.18	0.25
$ClusterCoef_i \approx \left(1 + (Heterogeneity)^2\right)^2 \times Density$	0.99	0.97	0.95	0.92	0.89	0.86	0.83	0.21	0.27
$ModuleSignif \approx \sqrt{Density} \times HubGeneSignif$	1.0	0.99	0.98	0.98	0.97	0.95	0.94	0.99	0.99
$Centralization \approx \sqrt{Density}(1 - \sqrt{Density})$	0.51	0.24	0.04	0.06	0.33	0.53	0.68	0.76	0.98
$\frac{k_{max}}{n-1} \approx \sqrt{Density}$	0.89	0.91	0.93	0.94	0.95	0.96	0.96	0.51	0.20
$K_i \approx a_{e,i}$ (median R^2)	1.0	0.99	0.99	0.98	0.97	0.97	0.96	0.93	0.92

Overall, we find that our theoretical results are highly robust in weighted networks. The relation $Centralization^{(q)} \approx \sqrt{Density^{(q)}}(1 - \sqrt{Density^{(q)}})$ breaks down for $\beta = 3, 4$, but the other relationships between network concepts are highly robust with respect to β. For unweighted networks, the R^2 values tend to be lower and several relationships show a marked dependency on the hard threshold τ (Table 1).

Our robustness analysis shows that many of our theoretical results apply even if our underlying assumptions are not satisfied. We find that the correspondence between network concepts and their eigengene-based analogs is often better in weighted networks than in unweighted networks. Further, we find that results in weighted networks tend to be more robust than those in unweighted networks with regard to changing the network construction thresholds β and τ, respectively. Thus, weighted co-expression networks are preferable over unweighted networks when a geometric interpretation of network concepts is desirable.

The correspondence between co-expression module networks and the singular value decomposition (Table 1 in the main article) can break down when a high soft threshold is used for constructing a weighted network or when dealing with an unweighted network. Thus, eigengene-based concepts do not replace network concepts when describing interaction patterns between genes.

4 Weighted Gene Co-Expression Network Results for $\beta = 1$

Table 2: Values of network concepts for a weighted network constructed with a soft threshold of $\beta = 1$.

Module	black	blue	brown	green	grey	pink	red	turquoise	yellow
Size ($n^{(q)}$)	67	215	139	98	920	53	96	308	105
Eigengene Factorizability ($EF(X^{(q)})$)	0.969	0.964	0.977	0.967	0.553	0.97	0.975	0.972	0.975
$VarExplained(\boldsymbol{E}^{(q)})$	0.633	0.58	0.62	0.599	0.25	0.697	0.608	0.576	0.596
$max(a_{e,i})$	0.964	0.962	0.917	0.929	0.945	0.965	0.965	0.933	0.943
$Density$	0.615	0.567	0.606	0.588	0.285	0.737	0.597	0.566	0.583
$Density_E$	0.63	0.571	0.615	0.598	0.203	0.732	0.607	0.568	0.596
$Centralization$	0.144	0.159	0.109	0.128	0.144	0.0996	0.155	0.136	0.14
$Centralization_E$	0.145	0.159	0.108	0.127	0.223	0.106	0.151	0.137	0.138
$Heterogeneity$	0.138	0.133	0.108	0.102	0.152	0.127	0.102	0.124	0.103
$Heterogeneity_E$	0.137	0.133	0.108	0.102	0.477	0.146	0.101	0.125	0.1
$Mean(ClusterCoef)$	0.641	0.589	0.621	0.602	0.314	0.761	0.61	0.585	0.596
$ClusterCoef_E$	0.644	0.589	0.624	0.604	0.306	0.749	0.613	0.584	0.602
$ModuleSignif$	0.164	0.233	0.266	0.276	0.136	0.17	0.0208	0.458	0.00952
$HubGeneSignif$	0.204	0.303	0.311	0.322	0.205	0.195	0.0244	0.579	0.0121

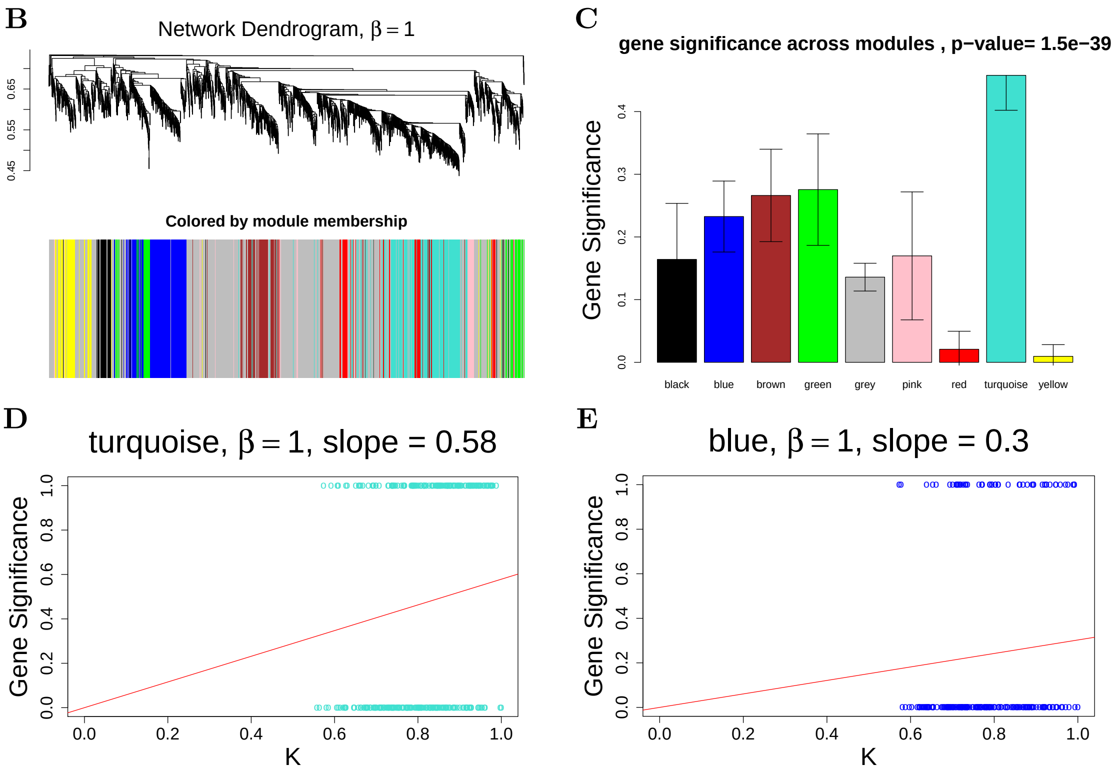

Figure 2: This figure is analogous to Figure 3 in the main article. The only difference is that we use a different dissimilarity for constructing the cluster tree in Figure B. Specifically, we use the topological overlap matrix based dissimilarity corresponding to a weighted network constructed with $\beta = 1$. Figure B depicts the hierarchical cluster tree of genes. Modules correspond to branches of the tree. The branches and module genes are assigned a color as can be seen from the color-bands underneath the tree. Grey denotes genes outside of proper modules. Figure C shows the module significance (average gene significance) of the modules. The underlying gene significance is defined as the knock out essentiality. Figures D and E show scatter plots of gene significance GS (y-axis) versus scaled connectivity K (x-axis) in the turquoise and blue module, respectively. The hub gene significance is defined as the slope of the red line, which results from a regression model without an intercept term.

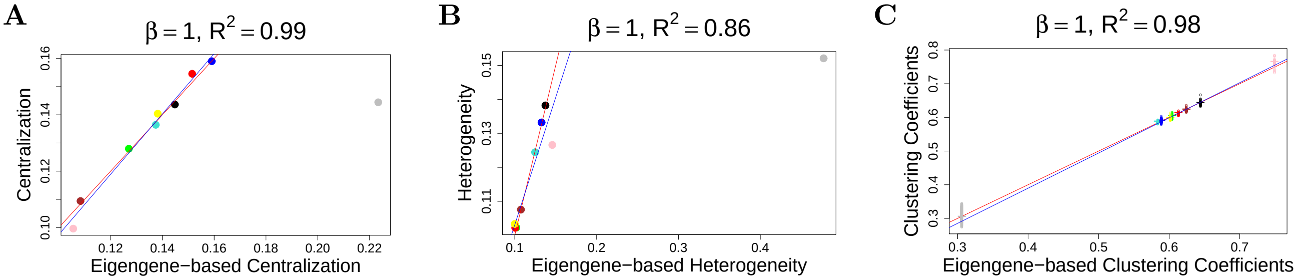

Figure 3: This figure is analogous to Figure 6 in the main article, corresponding to a weighted network constructed with a soft threshold of $\beta = 1$. It illustrates Observation 2 regarding the relationship between network concepts (y-axis) and their eigengene-based analogs (x-axis) in the mouse data. Each point corresponds to a module. Figure A: Centralization (y-axis) versus eigengene-based Centralization$_E$ (x-axis); analogous plots for Figure B: Heterogeneity; Figure C: clustering coefficient. The blue line is the regression line through the points representing proper modules (i.e., the grey, non-module genes are left out). While the red reference line (slope 1, intercept 0) does not always fit well, we observe high squared correlations R^2 between network concepts and their analogs. Since the grey point corresponds to the genes outside properly defined modules, we did not include it in calculations.

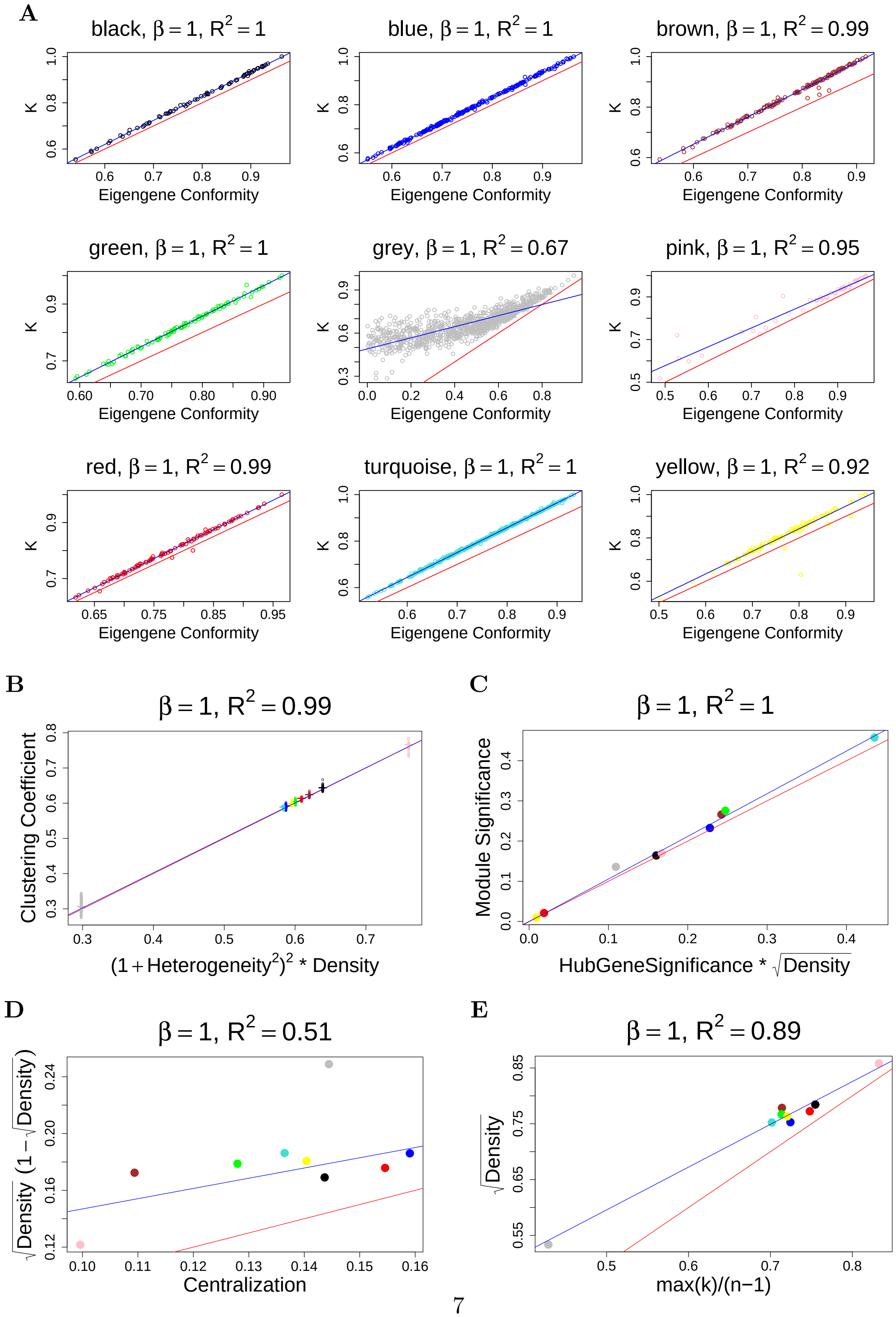

Figure 4: This figure is analogous to Figure 8 in the main article, corresponding to a weighted network constructed with a soft threshold of $\beta = 1$. It illustrates Observation 3 regarding the relationships among network concepts.

5 Weighted Gene Co-Expression Network Results for $\beta = 2$

Table 3: Values of network concepts for a weighted network constructed with a soft threshold of $\beta = 2$.

Module	black	blue	brown	green	grey	pink	red	turquoise	yellow
Size ($n^{(q)}$)	67	215	139	98	920	53	96	308	105
Eigengene Factorizability ($EF(X^{(q)})$)	0.969	0.964	0.977	0.967	0.553	0.97	0.975	0.972	0.975
$VarExplained(\boldsymbol{E}^{(q)})$	0.633	0.58	0.62	0.599	0.25	0.697	0.608	0.576	0.596
$max(a_{e,i})$	0.928	0.926	0.841	0.864	0.892	0.932	0.93	0.871	0.889
$Density$	0.404	0.345	0.384	0.364	0.113	0.568	0.372	0.339	0.355
$Density_E$	0.406	0.337	0.384	0.361	0.0622	0.549	0.372	0.332	0.358
$Centralization$	0.184	0.192	0.136	0.156	0.118	0.14	0.199	0.164	0.175
$Centralization_E$	0.196	0.204	0.141	0.164	0.161	0.154	0.203	0.172	0.18
$Heterogeneity$	0.244	0.242	0.195	0.186	0.254	0.217	0.193	0.227	0.188
$Heterogeneity_E$	0.263	0.264	0.206	0.201	0.743	0.255	0.201	0.243	0.197
$Mean(ClusterCoef)$	0.46	0.393	0.417	0.395	0.157	0.622	0.402	0.379	0.384
$ClusterCoef_E$	0.457	0.383	0.414	0.387	0.15	0.611	0.399	0.371	0.383
$ModuleSignif$	0.164	0.233	0.266	0.276	0.136	0.17	0.0208	0.458	0.00952
$HubGeneSignif$	0.238	0.363	0.35	0.361	0.27	0.211	0.0273	0.688	0.0145

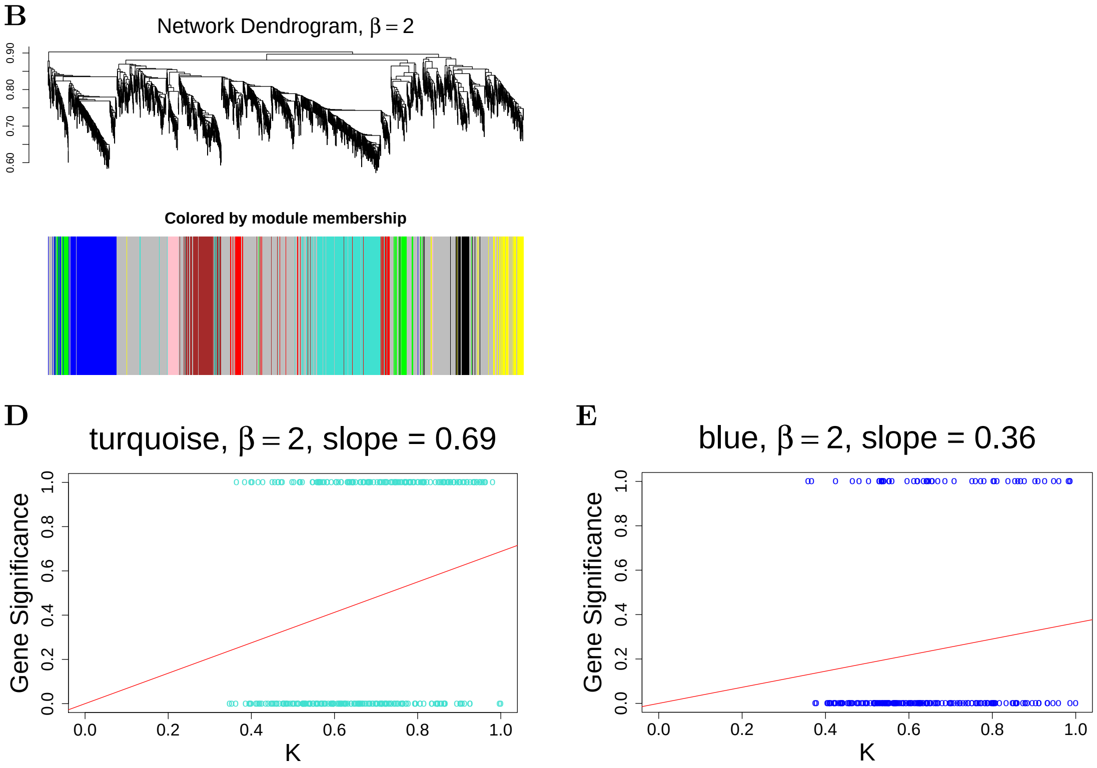

Figure 5: This figure is analogous to Figure 3 in the main article. The only difference is that we use a different dissimilarity for constructing the cluster tree in Figure B. Specifically, we use the topological overlap matrix based dissimilarity corresponding to a weighted network constructed with $\beta = 2$. Figure B depicts the hierarchical cluster tree of genes. Modules correspond to branches of the tree. The branches and module genes are assigned a color as can be seen from the color-bands underneath the tree. Grey denotes genes outside of proper modules. Figure C would be identical to the case of $\beta = 1$, and thus is omitted here. Figures D and E show scatter plots of gene significance GS (y-axis) versus scaled connectivity K (x-axis) in the turquoise and blue module, respectively. The underlying gene significance is defined as the knock out essentiality. The hub gene significance is defined as the slope of the red line, which results from a regression model without an intercept term.

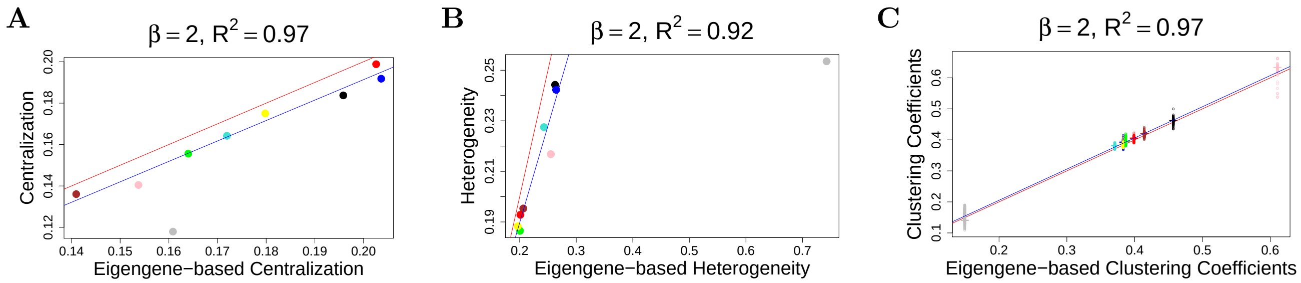

Figure 6: This figure is analogous to Figure 6 in the main article, corresponding to a weighted network constructed with a soft threshold of $\beta = 2$. It illustrates Observation 2 regarding the relationship between network concepts (y-axis) and their eigengene-based analogs (x-axis) in the mouse data. Each point corresponds to a module. Figure A: Centralization (y-axis) versus eigengene-based Centralization$_E$ (x-axis); analogous plots for Figure B: Heterogeneity; Figure C: clustering coefficient. The blue line is the regression line through the points representing proper modules (i.e., the grey, non-module genes are left out). While the red reference line (slope 1, intercept 0) does not always fit well, we observe high squared correlations R^2 between network concepts and their analogs. Since the grey point corresponds to the genes outside properly defined modules, we did not include it in calculations.

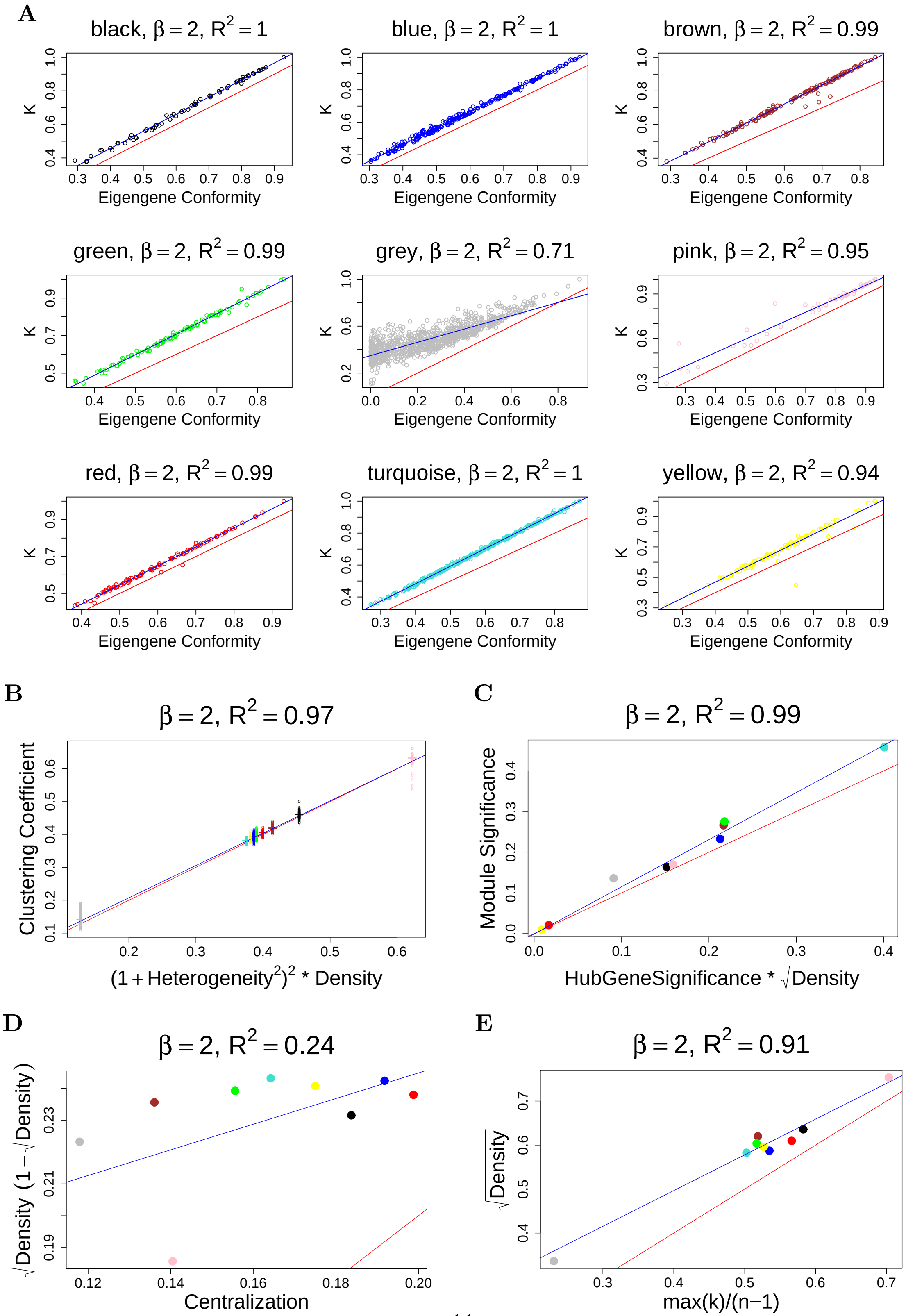

Figure 7: This figure is analogous to Figure 8 in the main article, corresponding to a weighted network constructed with a soft threshold of $\beta = 2$. It illustrates Observation 3 regarding the relationships among network concepts.

6 Weighted Gene Co-Expression Network Results for $\beta = 3$

Table 4: Values of network concepts for a weighted network constructed with a soft threshold of $\beta = 3$.

Module	black	blue	brown	green	grey	pink	red	turquoise	yellow
Size ($n^{(q)}$)	67	215	139	98	920	53	96	308	105
Eigengene Factorizability ($EF(X^{(q)})$)	0.969	0.964	0.977	0.967	0.553	0.97	0.975	0.972	0.975
$VarExplained(\boldsymbol{E}^{(q)})$	0.633	0.58	0.62	0.599	0.25	0.697	0.608	0.576	0.596
$max(a_{e,i})$	0.894	0.891	0.771	0.803	0.843	0.899	0.897	0.813	0.838
$Density$	0.278	0.221	0.252	0.235	0.0526	0.451	0.24	0.213	0.224
$Density_E$	0.27	0.205	0.245	0.223	0.0228	0.424	0.233	0.199	0.22
$Centralization$	0.187	0.183	0.132	0.15	0.0844	0.156	0.198	0.155	0.17
$Centralization_E$	0.204	0.201	0.14	0.162	0.105	0.174	0.207	0.165	0.178
$Heterogeneity$	0.336	0.338	0.271	0.261	0.333	0.285	0.276	0.318	0.265
$Heterogeneity_E$	0.377	0.395	0.298	0.297	0.964	0.339	0.302	0.355	0.293
$Mean(ClusterCoef)$	0.352	0.284	0.296	0.277	0.0963	0.525	0.282	0.263	0.262
$ClusterCoef_E$	0.347	0.273	0.288	0.261	0.0848	0.517	0.275	0.252	0.257
$ModuleSignif$	0.164	0.233	0.266	0.276	0.136	0.17	0.0208	0.458	0.00952
$HubGeneSignif$	0.269	0.413	0.383	0.393	0.333	0.222	0.0296	0.785	0.0169

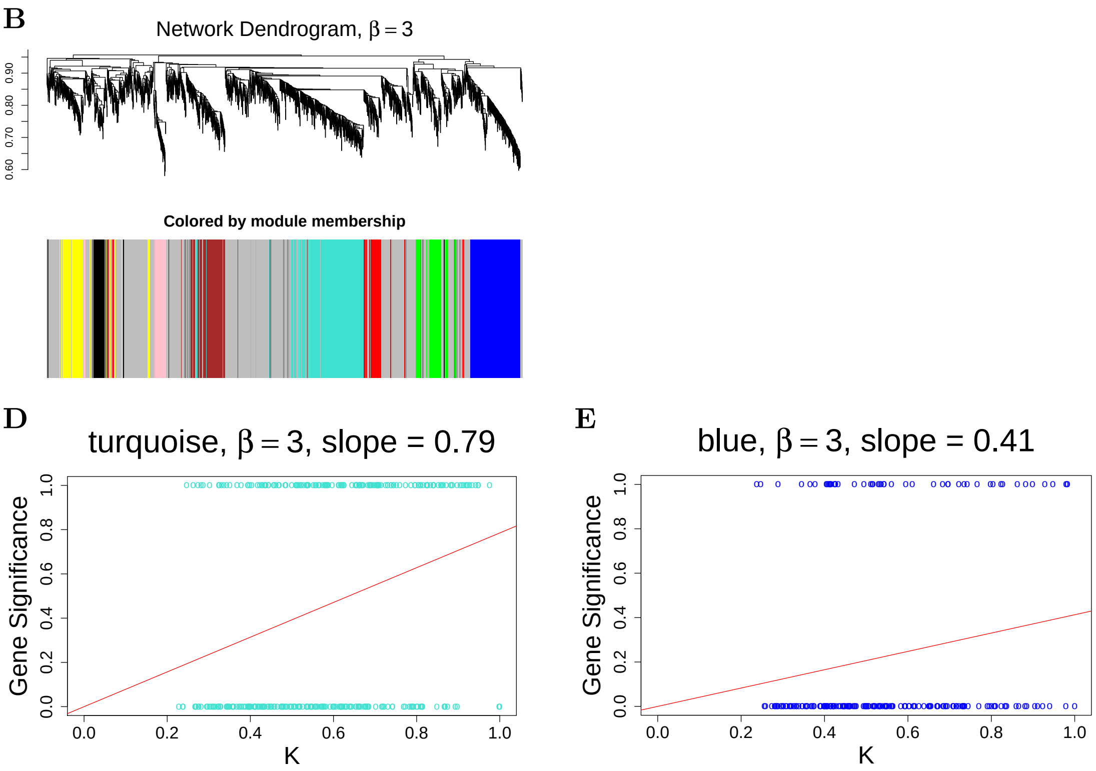

Figure 8: This figure is analogous to Figure 3 in the main article. The only difference is that we use a different dissimilarity for constructing the cluster tree in Figure B. Specifically, we use the topological overlap matrix based dissimilarity corresponding to a weighted network constructed with $\beta = 3$. Figure B depicts the hierarchical cluster tree of genes. Modules correspond to branches of the tree. The branches and module genes are assigned a color as can be seen from the color-bands underneath the tree. Grey denotes genes outside of proper modules. Figure C would be identical to the case of $\beta = 1$, and thus is omitted here. Figures D and E show scatter plots of gene significance GS (y-axis) versus scaled connectivity K (x-axis) in the turquoise and blue module, respectively. The underlying gene significance is defined as the knock out essentiality. The hub gene significance is defined as the slope of the red line, which results from a regression model without an intercept term.

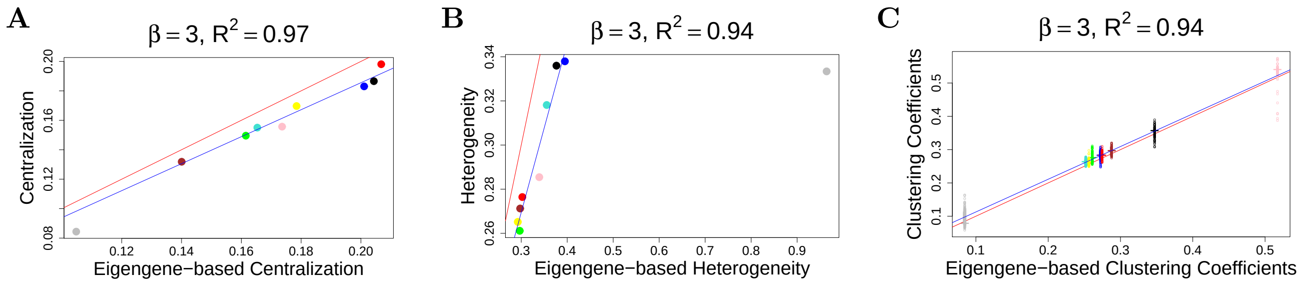

Figure 9: This figure is analogous to Figure 6 in the main article, corresponding to a weighted network constructed with a soft threshold of $\beta = 3$. It illustrates Observation 2 regarding the relationship between network concepts (y-axis) and their eigengene-based analogs (x-axis) in the mouse data. Each point corresponds to a module. Figure A: Centralization (y-axis) versus eigengene-based Centralization$_E$ (x-axis); analogous plots for Figure B: Heterogeneity; Figure C: clustering coefficient. The blue line is the regression line through the points representing proper modules (i.e., the grey, non-module genes are left out). While the red reference line (slope 1, intercept 0) does not always fit well, we observe high squared correlations R^2 between network concepts and their analogs. Since the grey point corresponds to the genes outside properly defined modules, we did not include it in calculations.

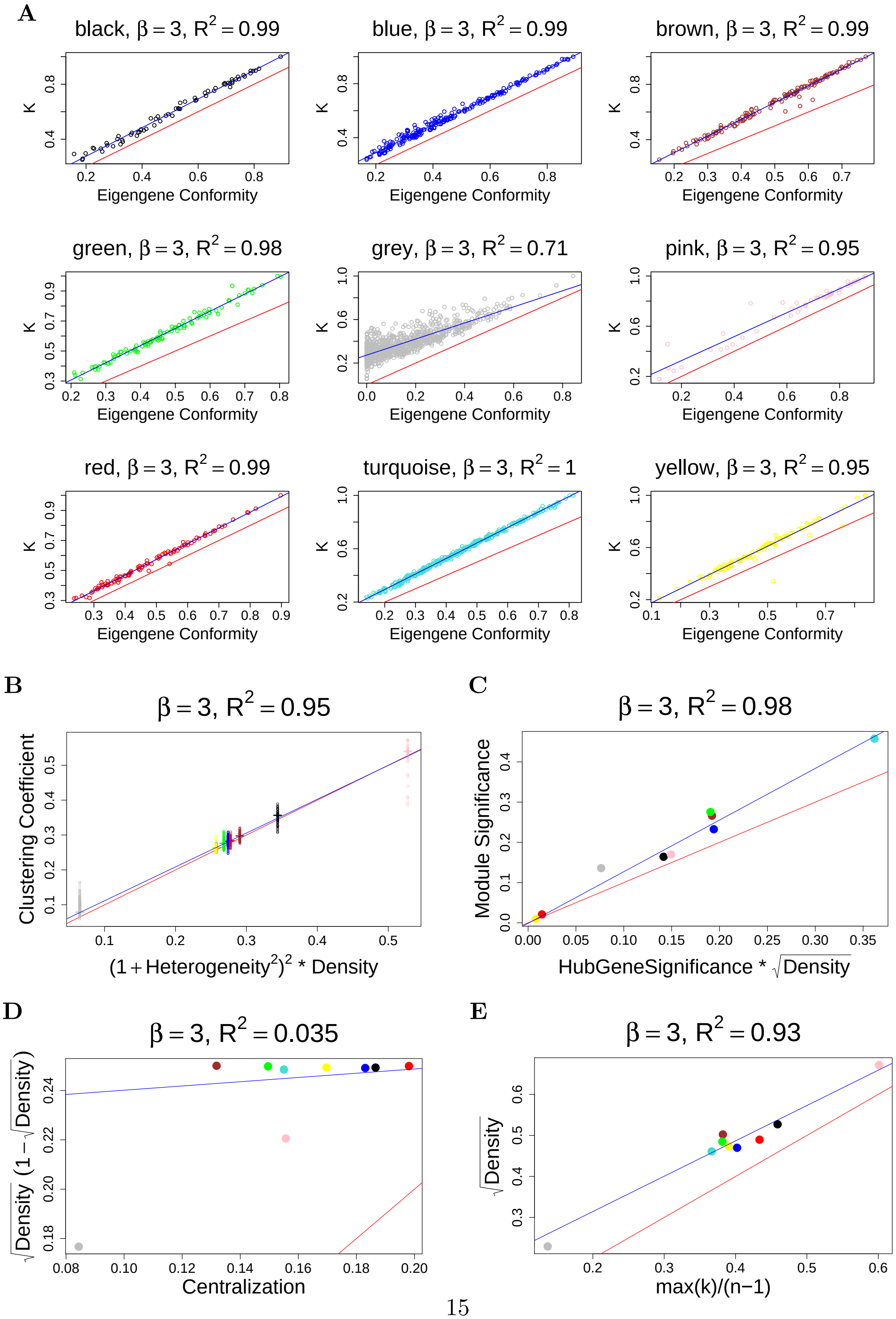

Figure 10: This figure is analogous to Figure 8 in the main article, corresponding to a weighted network constructed with a soft threshold of $\beta = 3$. It illustrates Observation 3 regarding the relationships among network concepts.

7 Weighted Gene Co-Expression Network Results for $\beta = 4$

Table 5: Values of network concepts for a weighted network constructed with a soft threshold of $\beta = 4$.

Module	black	blue	brown	green	grey	pink	red	turquoise	yellow
Size ($n^{(q)}$)	67	215	139	98	920	53	96	308	105
Eigengene Factorizability ($EF(X^{(q)})$)	0.969	0.964	0.977	0.967	0.553	0.97	0.975	0.972	0.975
$VarExplained(\boldsymbol{E}^{(q)})$	0.633	0.58	0.62	0.599	0.25	0.697	0.608	0.576	0.596
$max(a_{e,i})$	0.862	0.857	0.707	0.746	0.796	0.868	0.866	0.759	0.79
$Density$	0.198	0.147	0.17	0.156	0.0269	0.366	0.159	0.138	0.145
$Density_E$	0.186	0.129	0.159	0.14	0.00931	0.335	0.149	0.123	0.137
$Centralization$	0.176	0.162	0.117	0.135	0.059	0.161	0.18	0.135	0.15
$Centralization_E$	0.194	0.181	0.126	0.144	0.0677	0.179	0.191	0.145	0.16
$Heterogeneity$	0.418	0.425	0.339	0.331	0.402	0.34	0.355	0.4	0.338
$Heterogeneity_E$	0.481	0.524	0.382	0.391	1.18	0.406	0.404	0.463	0.389
$Mean(ClusterCoef)$	0.281	0.218	0.218	0.204	0.0662	0.45	0.208	0.192	0.187
$ClusterCoef_E$	0.277	0.209	0.207	0.184	0.0532	0.446	0.199	0.181	0.18
$ModuleSignif$	0.164	0.233	0.266	0.276	0.136	0.17	0.0208	0.458	0.00952
$HubGeneSignif$	0.295	0.455	0.411	0.423	0.392	0.232	0.0313	0.873	0.0191

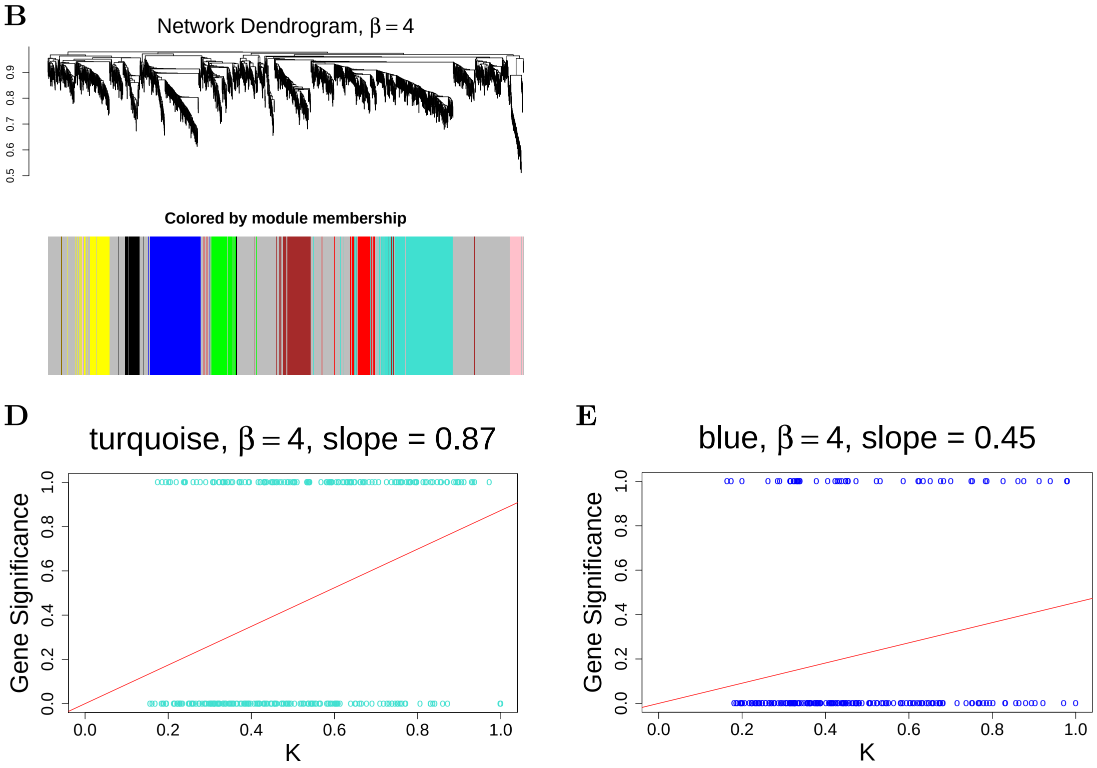

Figure 11: This figure is analogous to Figure 3 in the main article. The only difference is that we use a different dissimilarity for constructing the cluster tree in Figure B. Specifically, we use the topological overlap matrix based dissimilarity corresponding to a weighted network constructed with $\beta = 4$. Figure B depicts the hierarchical cluster tree of genes. Modules correspond to branches of the tree. The branches and module genes are assigned a color as can be seen from the color-bands underneath the tree. Grey denotes genes outside of proper modules. Figure C would be identical to the case of $\beta = 1$, and thus is omitted here. Figures D and E show scatter plots of gene significance GS (y-axis) versus scaled connectivity K (x-axis) in the turquoise and blue module, respectively. The underlying gene significance is defined as the knock out essentiality. The hub gene significance is defined as the slope of the red line, which results from a regression model without an intercept term.

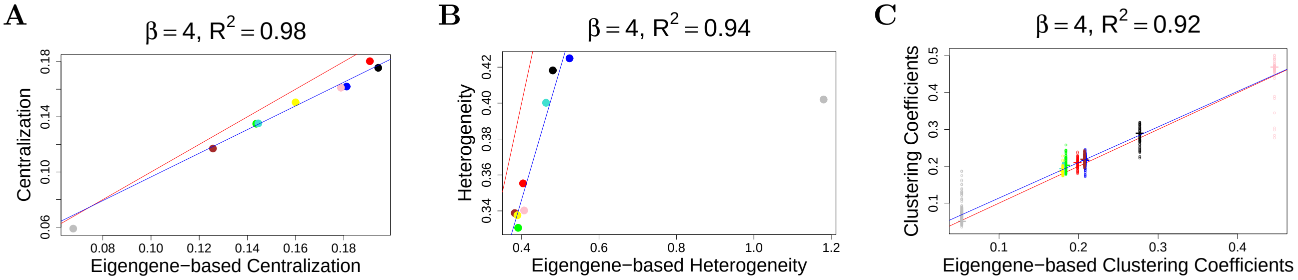

Figure 12: This figure is analogous to Figure 6 in the main article, corresponding to a weighted network constructed with a soft threshold of $\beta = 4$. It illustrates Observation 2 regarding the relationship between network concepts (y-axis) and their eigengene-based analogs (x-axis) in the mouse data. Each point corresponds to a module. Figure A: Centralization (y-axis) versus eigengene-based Centralization$_E$ (x-axis); analogous plots for Figure B: Heterogeneity; Figure C: clustering coefficient. The blue line is the regression line through the points representing proper modules (i.e., the grey, non-module genes are left out). While the red reference line (slope 1, intercept 0) does not always fit well, we observe high squared correlations R^2 between network concepts and their analogs. Since the grey point corresponds to the genes outside properly defined modules, we did not include it in calculations.

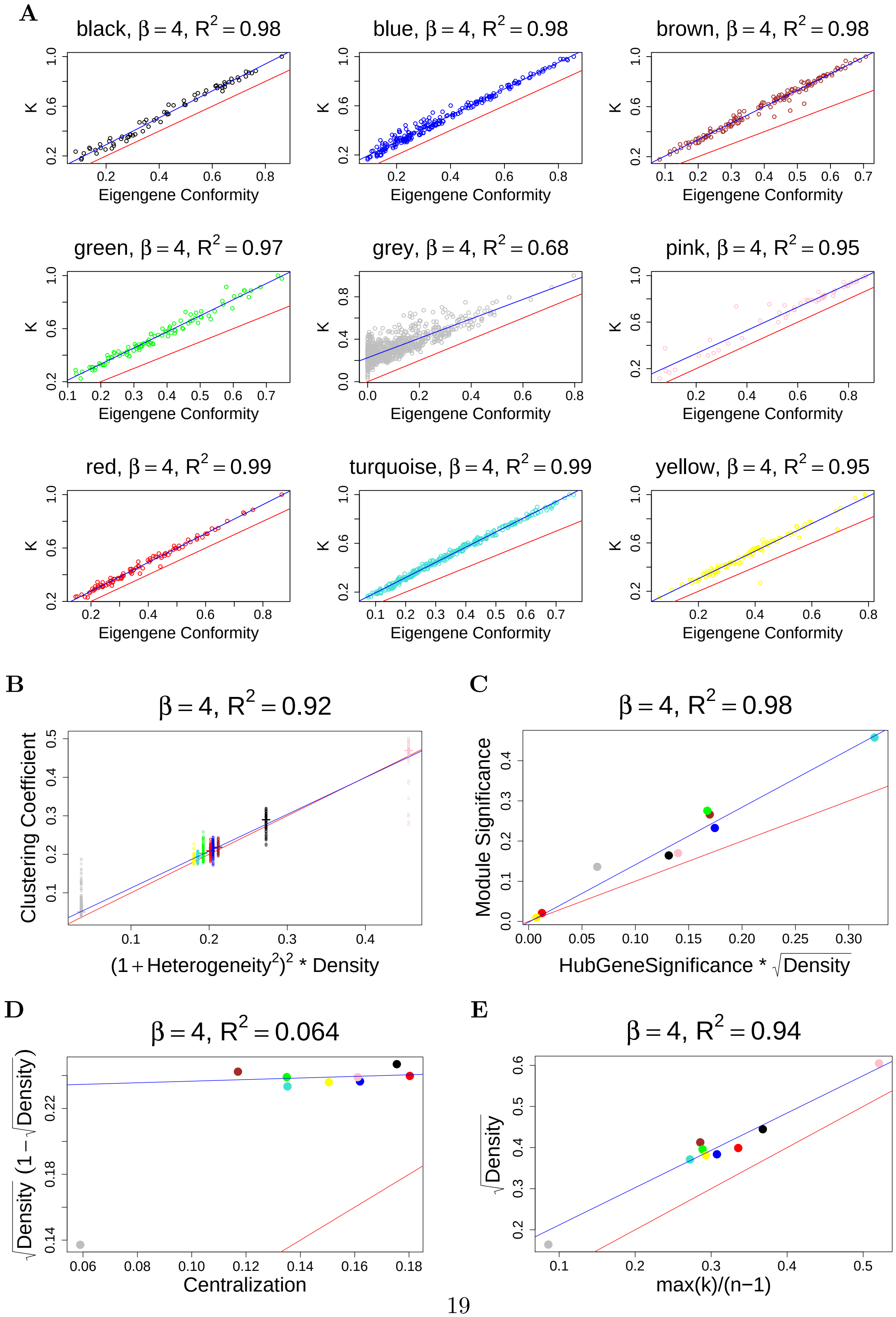

Figure 13: This figure is analogous to Figure 8 in the main article, corresponding to a weighted network constructed with a soft threshold of $\beta = 4$. It illustrates Observation 3 regarding the relationships among network concepts.

8 Weighted Gene Co-Expression Network Results for $\beta = 5$

Table 6: Values of network concepts for a weighted network constructed with a soft threshold of $\beta = 5$.

Module	black	blue	brown	green	grey	pink	red	turquoise	yellow
Size ($n^{(q)}$)	67	215	139	98	920	53	96	308	105
Eigengene Factorizability ($EF(X^{(q)})$)	0.969	0.964	0.977	0.967	0.553	0.97	0.975	0.972	0.975
$VarExplained(\boldsymbol{E}^{(q)})$	0.633	0.58	0.62	0.599	0.25	0.697	0.608	0.576	0.596
$max(a_{e,i})$	0.83	0.824	0.648	0.694	0.752	0.838	0.835	0.708	0.745
$Density$	0.145	0.101	0.118	0.107	0.0148	0.301	0.108	0.092	0.0968
$Density_E$	0.131	0.0837	0.105	0.0895	0.00411	0.269	0.0966	0.0778	0.0872
$Centralization$	0.16	0.139	0.0998	0.118	0.0413	0.16	0.157	0.114	0.128
$Centralization_E$	0.177	0.157	0.107	0.122	0.0442	0.176	0.168	0.121	0.137
$Heterogeneity$	0.493	0.506	0.4	0.397	0.464	0.386	0.431	0.476	0.407
$Heterogeneity_E$	0.576	0.651	0.462	0.484	1.41	0.462	0.506	0.567	0.488
$Mean(ClusterCoef)$	0.232	0.174	0.166	0.156	0.0493	0.39	0.159	0.145	0.139
$ClusterCoef_E$	0.228	0.169	0.153	0.135	0.0364	0.389	0.151	0.135	0.132
$ModuleSignif$	0.164	0.233	0.266	0.276	0.136	0.17	0.0208	0.458	0.00952
$HubGeneSignif$	0.32	0.489	0.436	0.45	0.447	0.239	0.0323	0.954	0.0211

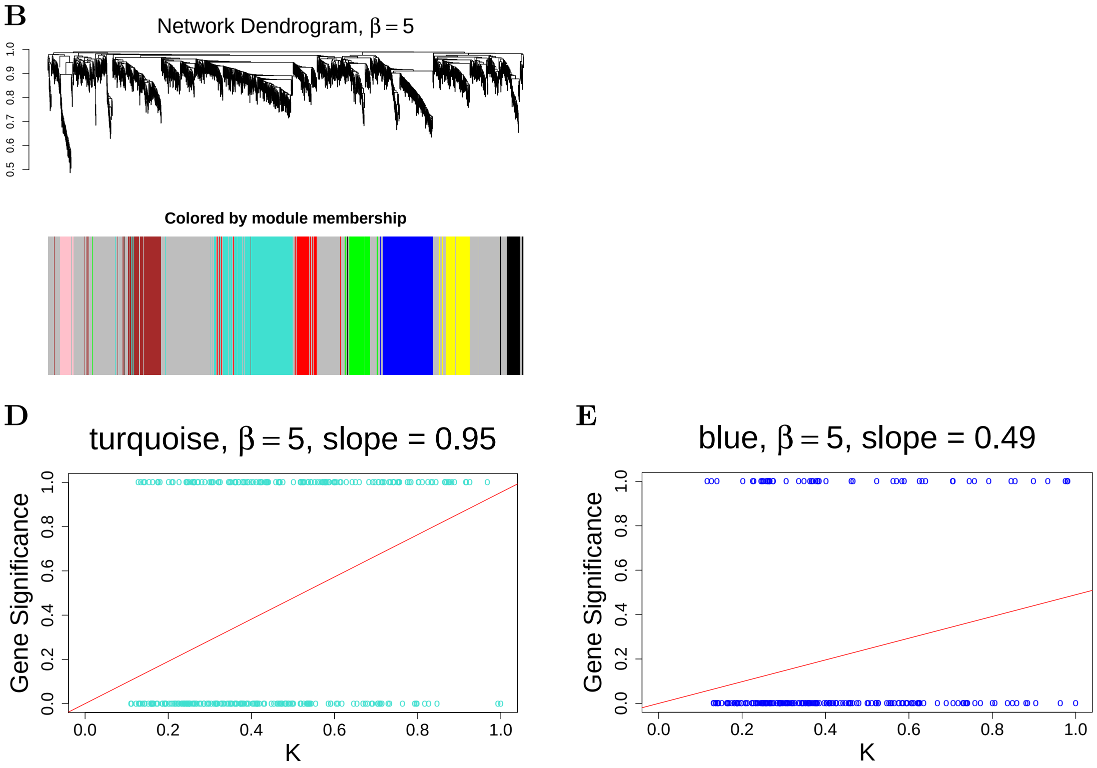

Figure 14: This figure is analogous to Figure 3 in the main article. The only difference is that we use a different dissimilarity for constructing the cluster tree in Figure B. Specifically, we use the topological overlap matrix based dissimilarity corresponding to a weighted network constructed with $\beta = 5$. Figure B depicts the hierarchical cluster tree of genes. Modules correspond to branches of the tree. The branches and module genes are assigned a color as can be seen from the color-bands underneath the tree. Grey denotes genes outside of proper modules. Figure C would be identical to the case of $\beta = 1$, and thus is omitted here. Figures D and E show scatter plots of gene significance GS (y-axis) versus scaled connectivity K (x-axis) in the turquoise and blue module, respectively. The underlying gene significance is defined as the knock out essentiality. The hub gene significance is defined as the slope of the red line, which results from a regression model without an intercept term.

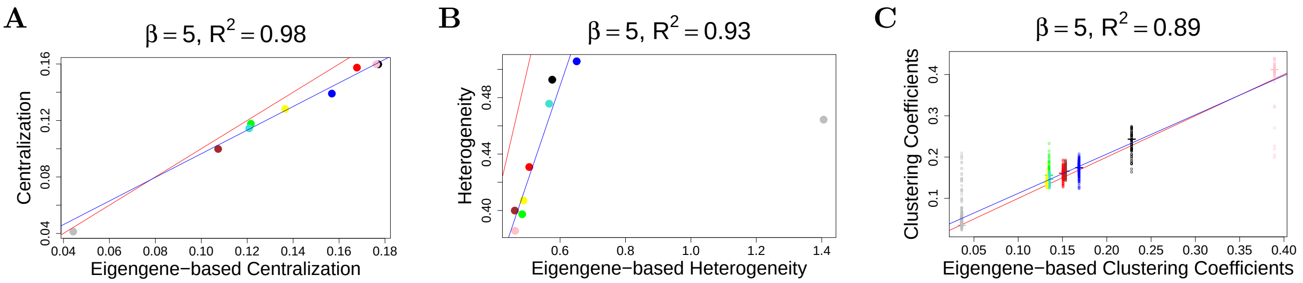

Figure 15: This figure is analogous to Figure 6 in the main article, corresponding to a weighted network constructed with a soft threshold of $\beta = 5$. It illustrates Observation 2 regarding the relationship between network concepts (y-axis) and their eigengene-based analogs (x-axis) in the mouse data. Each point corresponds to a module. Figure A: Centralization (y-axis) versus eigengene-based Centralization$_E$ (x-axis); analogous plots for Figure B: Heterogeneity; Figure C: clustering coefficient. The blue line is the regression line through the points representing proper modules (i.e., the grey, non-module genes are left out). While the red reference line (slope 1, intercept 0) does not always fit well, we observe high squared correlations R^2 between network concepts and their analogs. Since the grey point corresponds to the genes outside properly defined modules, we did not include it in calculations.

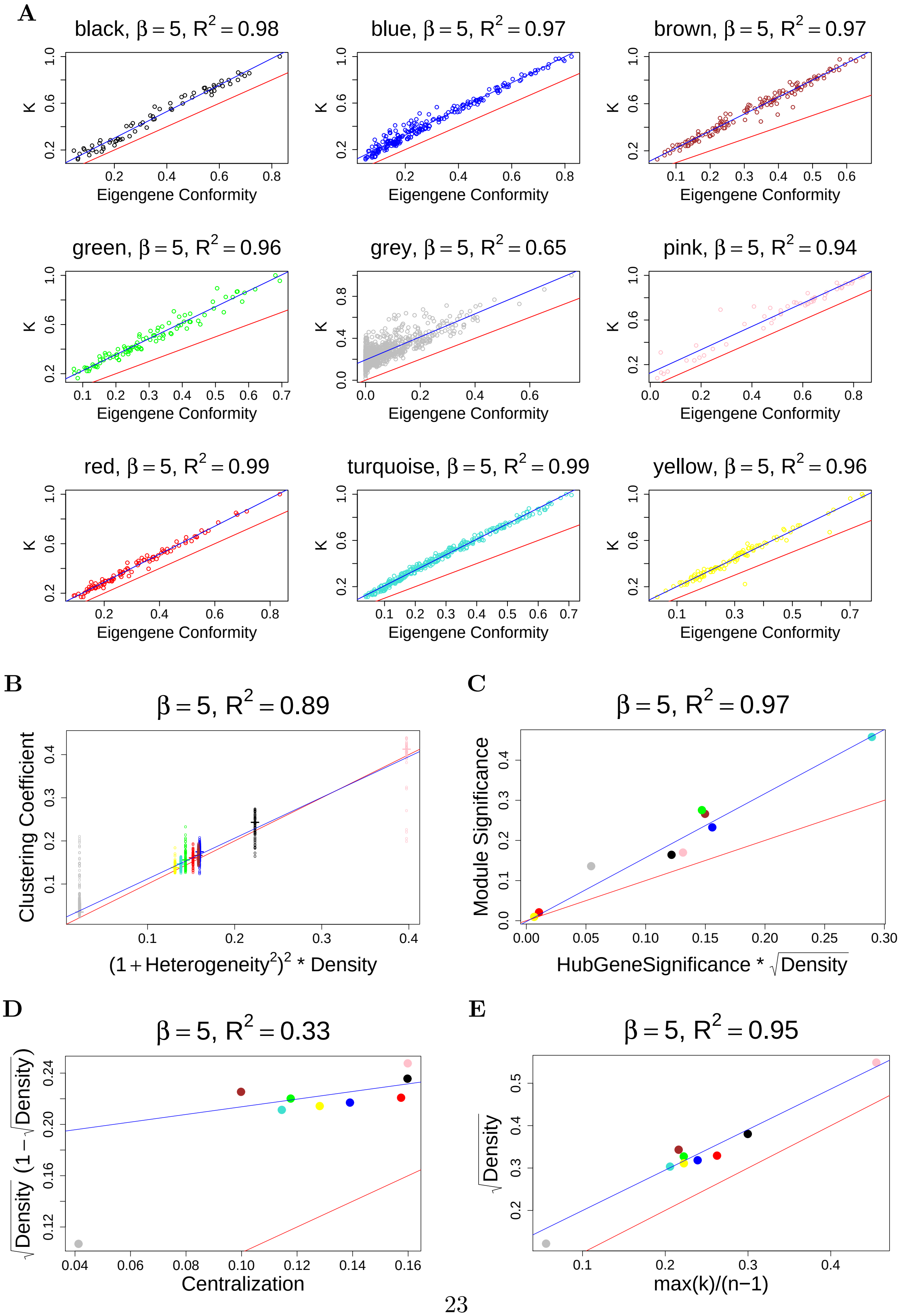

Figure 16: This figure is analogous to Figure 8 in the main article, corresponding to a weighted network constructed with a soft threshold of $\beta = 5$. It illustrates Observation 3 regarding the relationships among network concepts.

9 Weighted Gene Co-Expression Network Results for $\beta = 6$

Table 7: Values of network concepts for a weighted network constructed with a soft threshold of $\beta = 6$.

Module	black	blue	brown	green	grey	pink	red	turquoise	yellow
Size ($n^{(q)}$)	67	215	139	98	920	53	96	308	105
Eigengene Factorizability ($EF(X^{(q)})$)	0.969	0.964	0.977	0.967	0.553	0.97	0.975	0.972	0.975
$VarExplained(\boldsymbol{E}^{(q)})$	0.633	0.58	0.62	0.599	0.25	0.697	0.608	0.576	0.596
$max(a_{e,i})$	0.8	0.793	0.594	0.645	0.711	0.808	0.805	0.661	0.703
$Density$	0.108	0.0718	0.0831	0.075	0.00858	0.251	0.0755	0.063	0.0658
$Density_E$	0.094	0.0559	0.0705	0.0582	0.00193	0.219	0.064	0.0503	0.0564
$Centralization$	0.143	0.118	0.0834	0.101	0.0292	0.155	0.135	0.0954	0.107
$Centralization_E$	0.158	0.133	0.0892	0.1	0.0294	0.169	0.144	0.0988	0.113
$Heterogeneity$	0.56	0.582	0.456	0.463	0.524	0.424	0.504	0.546	0.475
$Heterogeneity_E$	0.664	0.775	0.536	0.576	1.65	0.511	0.608	0.666	0.589
$Mean(ClusterCoef)$	0.194	0.143	0.13	0.124	0.039	0.341	0.125	0.113	0.106
$ClusterCoef_E$	0.192	0.143	0.116	0.102	0.0267	0.342	0.119	0.105	0.101
$ModuleSignif$	0.164	0.233	0.266	0.276	0.136	0.17	0.0208	0.458	0.00952
$HubGeneSignif$	0.343	0.518	0.458	0.472	0.497	0.246	0.0329	1.03	0.023

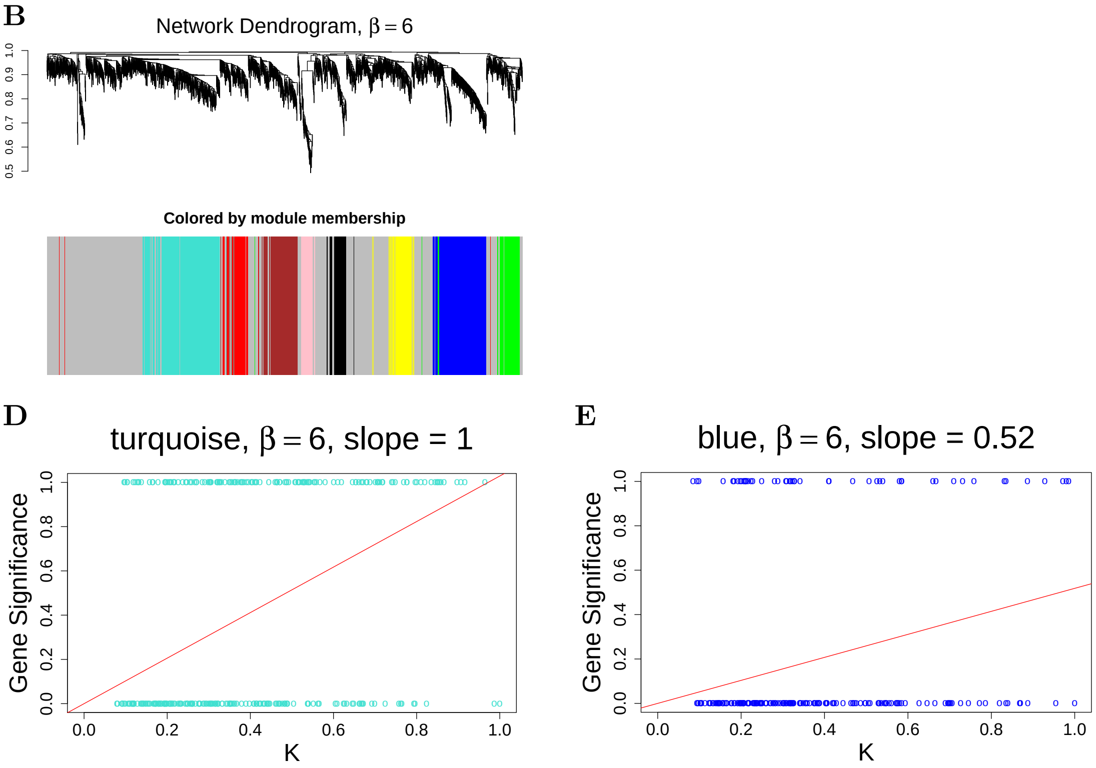

Figure 17: This figure is analogous to Figure 3 in the main article. The only difference is that we use a different dissimilarity for constructing the cluster tree in Figure B. Specifically, we use the topological overlap matrix based dissimilarity corresponding to a weighted network constructed with $\beta = 6$. Figure B depicts the hierarchical cluster tree of genes. Modules correspond to branches of the tree. The branches and module genes are assigned a color as can be seen from the color-bands underneath the tree. Grey denotes genes outside of proper modules. Figure C would be identical to the case of $\beta = 1$, and thus is omitted here. Figures D and E show scatter plots of gene significance GS (y-axis) versus scaled connectivity K (x-axis) in the turquoise and blue module, respectively. The underlying gene significance is defined as the knock out essentiality. The hub gene significance is defined as the slope of the red line, which results from a regression model without an intercept term.

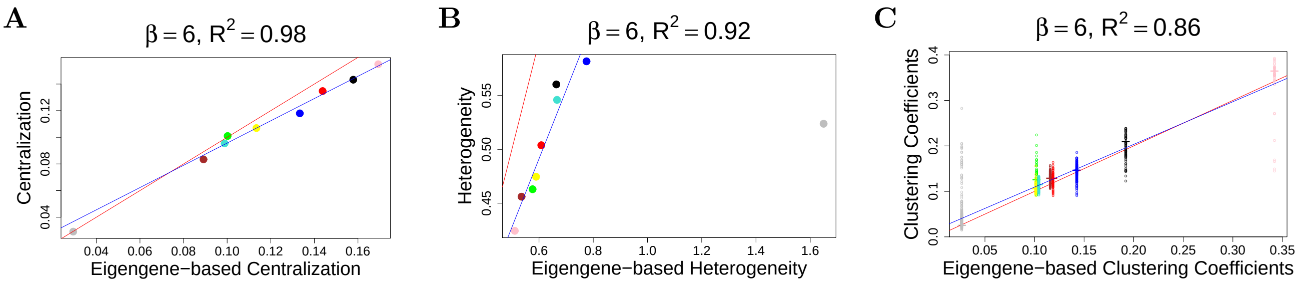

Figure 18: This figure is analogous to Figure 6 in the main article, corresponding to a weighted network constructed with a soft threshold of $\beta = 6$. It illustrates Observation 2 regarding the relationship between network concepts (y-axis) and their eigengene-based analogs (x-axis) in the mouse data. Each point corresponds to a module. Figure A: Centralization (y-axis) versus eigengene-based Centralization$_E$ (x-axis); analogous plots for Figure B: Heterogeneity; Figure C: clustering coefficient. The blue line is the regression line through the points representing proper modules (i.e., the grey, non-module genes are left out). While the red reference line (slope 1, intercept 0) does not always fit well, we observe high squared correlations R^2 between network concepts and their analogs. Since the grey point corresponds to the genes outside properly defined modules, we did not include it in calculations.

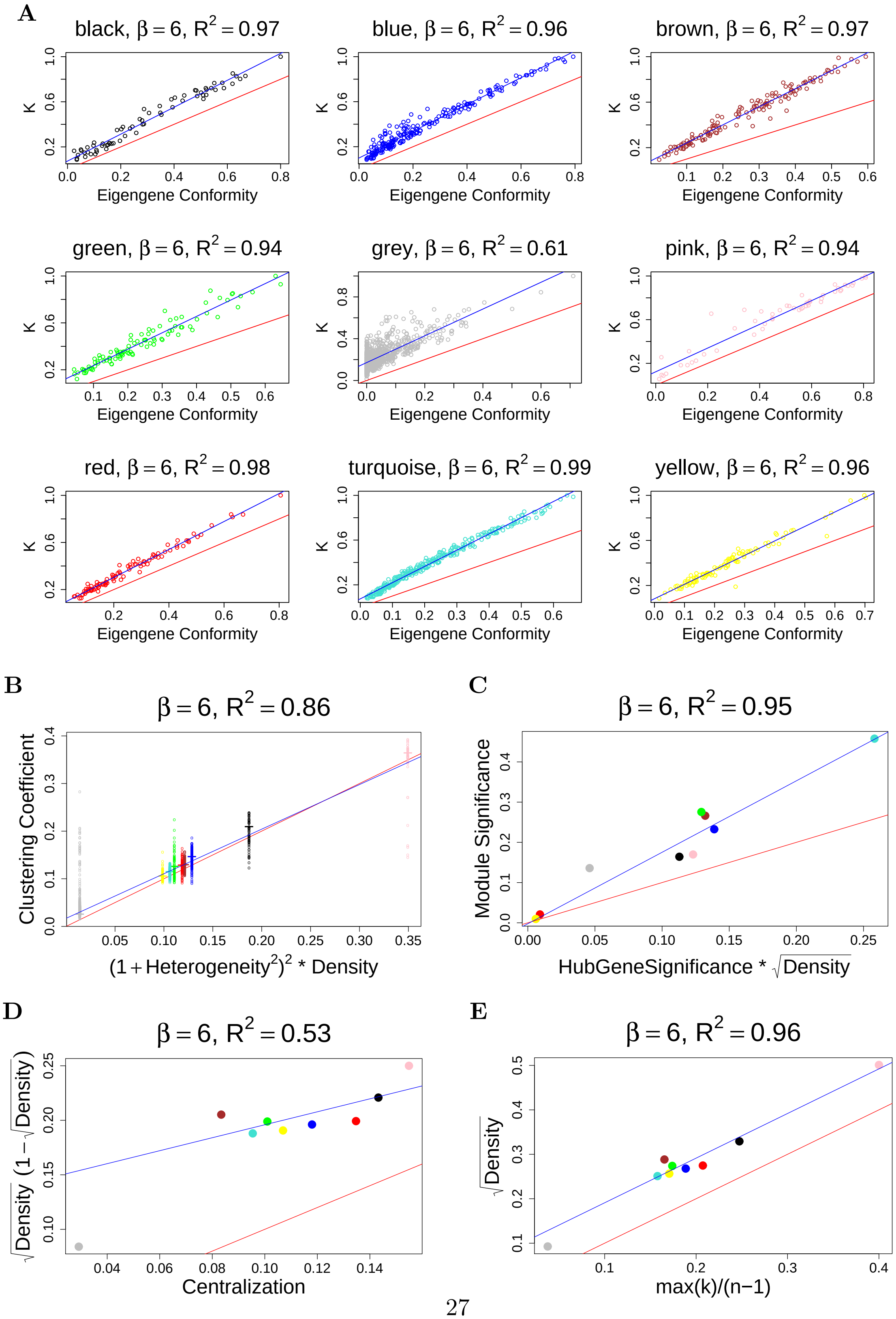

Figure 19: This figure is analogous to Figure 8 in the main article, corresponding to a weighted network constructed with a soft threshold of $\beta = 6$. It illustrates Observation 3 regarding the relationships among network concepts.

10 Weighted Gene Co-Expression Network Results for $\beta = 7$

Table 8: Values of network concepts for a weighted network constructed with a soft threshold of $\beta = 7$.

Module	black	blue	brown	green	grey	pink	red	turquoise	yellow
Size ($n^{(q)}$)	67	215	139	98	920	53	96	308	105
Eigengene Factorizability ($EF(X^{(q)})$)	0.969	0.964	0.977	0.967	0.553	0.97	0.975	0.972	0.975
$VarExplained(\boldsymbol{E}^{(q)})$	0.633	0.58	0.62	0.599	0.25	0.697	0.608	0.576	0.596
$max(a_{e,i})$	0.771	0.763	0.545	0.599	0.671	0.78	0.777	0.617	0.662
$Density$	0.0828	0.052	0.0597	0.0537	0.00521	0.211	0.0536	0.0441	0.0456
$Density_E$	0.0689	0.0383	0.0479	0.0384	0.000956	0.18	0.0431	0.0331	0.0371
$Centralization$	0.128	0.0998	0.0699	0.086	0.0209	0.148	0.114	0.079	0.0883
$Centralization_E$	0.139	0.112	0.0729	0.0813	0.0199	0.16	0.122	0.0799	0.0929
$Heterogeneity$	0.622	0.654	0.508	0.528	0.583	0.458	0.575	0.612	0.541
$Heterogeneity_E$	0.745	0.897	0.606	0.668	1.92	0.554	0.712	0.763	0.694
$Mean(ClusterCoef)$	0.165	0.121	0.103	0.101	0.0322	0.3	0.101	0.0903	0.0834
$ClusterCoef_E$	0.164	0.124	0.0888	0.0795	0.0209	0.302	0.0969	0.0827	0.0805
$ModuleSignif$	0.164	0.233	0.266	0.276	0.136	0.17	0.0208	0.458	0.00952
$HubGeneSignif$	0.365	0.542	0.481	0.49	0.54	0.251	0.033	1.1	0.0247

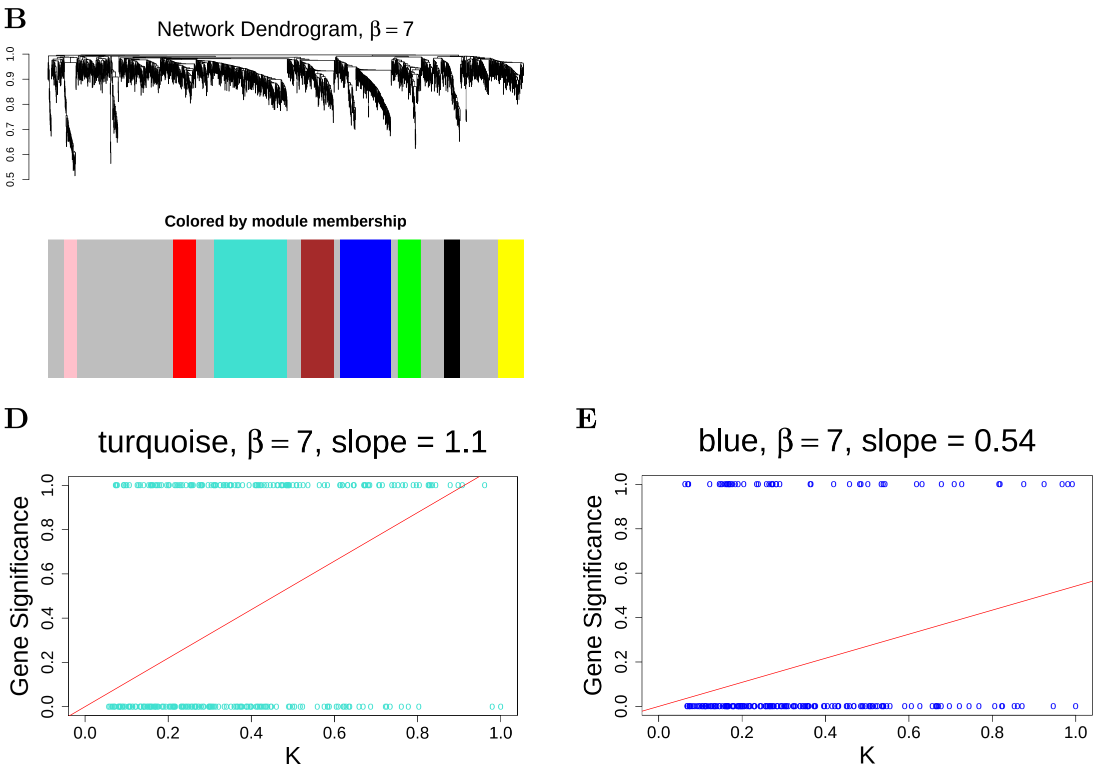

Figure 20: This figure is analogous to Figure 3 in the main article. The only difference is that we use a different dissimilarity for constructing the cluster tree in Figure B. Specifically, we use the topological overlap matrix based dissimilarity corresponding to a weighted network constructed with $\beta = 7$. Figure B depicts the hierarchical cluster tree of genes. Modules correspond to branches of the tree. The branches and module genes are assigned a color as can be seen from the color-bands underneath the tree. Grey denotes genes outside of proper modules. Figure C would be identical to the case of $\beta = 1$, and thus is omitted here. Figures D and E show scatter plots of gene significance GS (y-axis) versus scaled connectivity K (x-axis) in the turquoise and blue module, respectively. The underlying gene significance is defined as the knock out essentiality. The hub gene significance is defined as the slope of the red line, which results from a regression model without an intercept term.

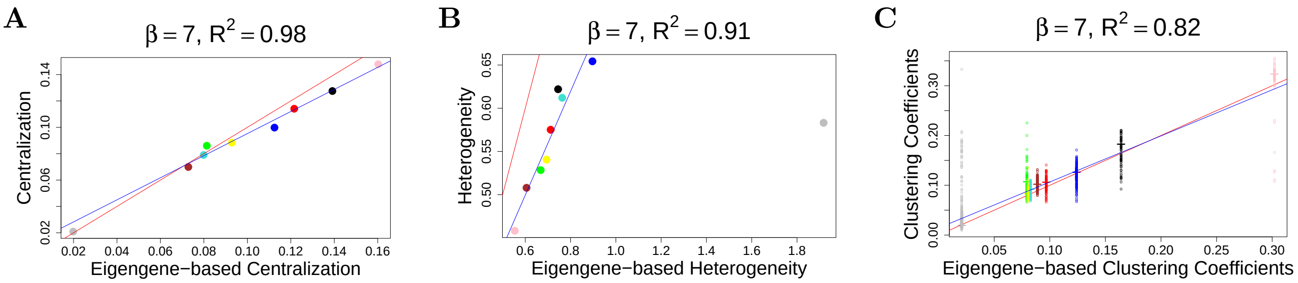

Figure 21: This figure is analogous to Figure 6 in the main article, corresponding to a weighted network constructed with a soft threshold of $\beta = 7$. It illustrates Observation 2 regarding the relationship between network concepts (y-axis) and their eigengene-based analogs (x-axis) in the mouse data. Each point corresponds to a module. Figure A: Centralization (y-axis) versus eigengene-based Centralization$_E$ (x-axis); analogous plots for Figure B: Heterogeneity; Figure C: clustering coefficient. The blue line is the regression line through the points representing proper modules (i.e., the grey, non-module genes are left out). While the red reference line (slope 1, intercept 0) does not always fit well, we observe high squared correlations R^2 between network concepts and their analogs. Since the grey point corresponds to the genes outside properly defined modules, we did not include it in calculations.

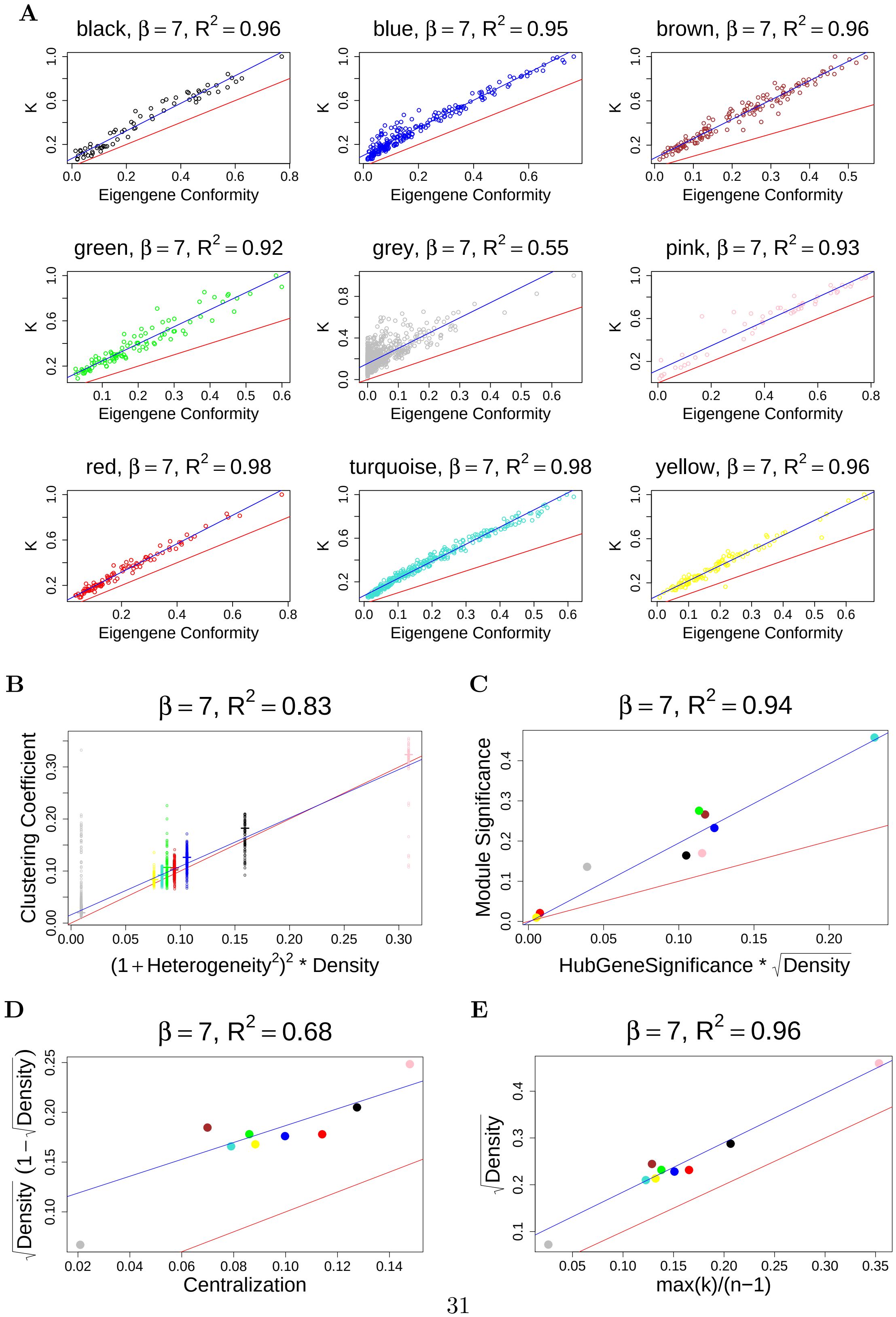

Figure 22: This figure is analogous to Figure 8 in the main article, corresponding to a weighted network constructed with a soft threshold of $\beta = 7$. It illustrates Observation 3 regarding the relationships among network concepts.

11 Unweighted Gene Co-Expression Network Results for $\tau = 0.65$

Table 9: Values of network concepts for an unweighted network constructed with a hard threshold of $\tau = 0.65$.

Module	black	blue	brown	green	grey	pink	red	turquoise	yellow
Size ($n^{(q)}$)	67	215	139	98	920	53	96	308	105
Eigengene Factorizability ($EF(X^{(q)})$)	0.969	0.964	0.977	0.967	0.553	0.97	0.975	0.972	0.975
$VarExplained(\boldsymbol{E}^{(q)})$	0.633	0.58	0.62	0.599	0.25	0.697	0.608	0.576	0.596
$max(a_{e,i})$	0.964	0.962	0.917	0.929	0.945	0.965	0.965	0.933	0.943
$Density$	0.44	0.316	0.4	0.352	0.0239	0.746	0.359	0.29	0.314
$Density_E$	0.63	0.571	0.615	0.598	0.203	0.732	0.607	0.568	0.596
$Centralization$	0.359	0.445	0.366	0.419	0.144	0.184	0.548	0.426	0.494
$Centralization_E$	0.145	0.159	0.108	0.127	0.223	0.106	0.151	0.137	0.138
$Heterogeneity$	0.498	0.585	0.506	0.469	0.832	0.308	0.531	0.631	0.563
$Heterogeneity_E$	0.137	0.133	0.108	0.102	0.477	0.146	0.101	0.125	0.1
$Mean(ClusterCoef)$	0.762	0.699	0.716	0.648	0.374	0.911	0.675	0.674	0.654
$ClusterCoef_E$	0.644	0.589	0.624	0.604	0.306	0.749	0.613	0.584	0.602
$ModuleSignif$	0.164	0.233	0.266	0.276	0.136	0.17	0.0208	0.458	0.00952
$HubGeneSignif$	0.256	0.467	0.421	0.435	0.611	0.211	0.0286	0.96	0.0209

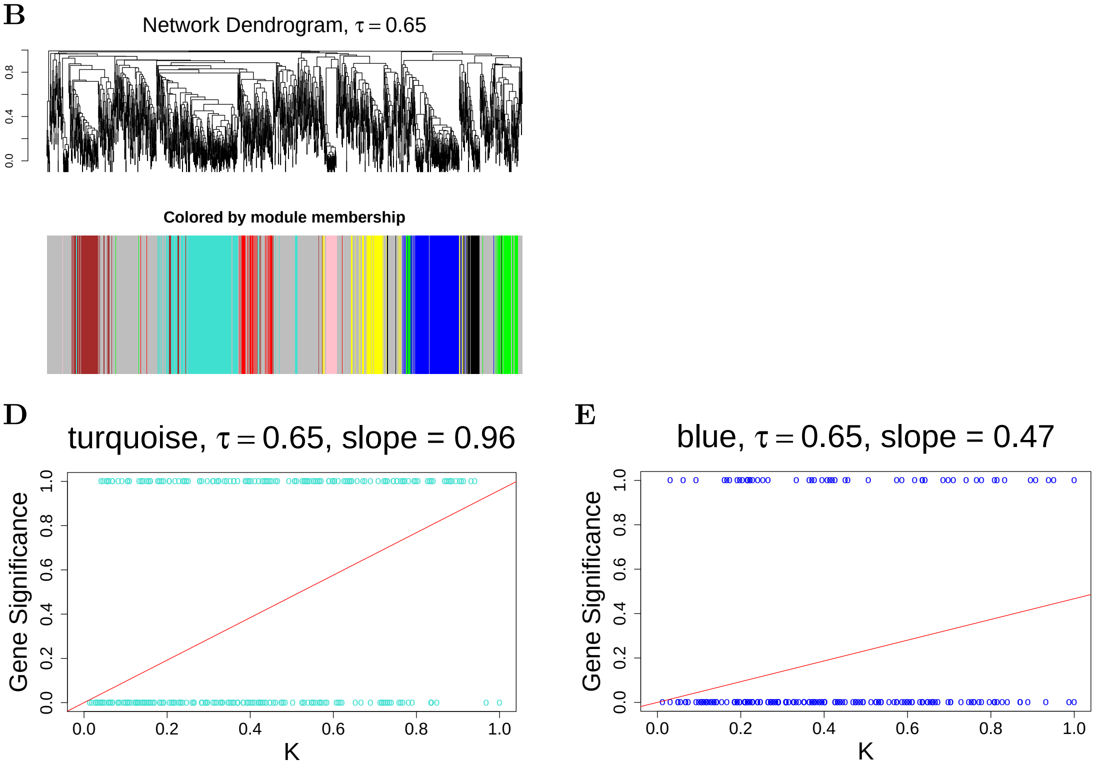

Figure 23: This figure is analogous to Figure 3 in the main article. The only difference is that we use a different dissimilarity for constructing the cluster tree in Figure B. Specifically, we use the topological overlap matrix based dissimilarity corresponding to an unweighted network constructed with $\tau = 0.65$. Figure B depicts the hierarchical cluster tree of genes. Modules correspond to branches of the tree. The branches and module genes are assigned a color as can be seen from the color-bands underneath the tree. Grey denotes genes outside of proper modules. Figure C would be identical to the case of $\beta = 1$, and thus is omitted here. Figures D and E show scatter plots of gene significance GS (y-axis) versus scaled connectivity K (x-axis) in the turquoise and blue module, respectively. The underlying gene significance is defined as the knock out essentiality. The hub gene significance is defined as the slope of the red line, which results from a regression model without an intercept term.

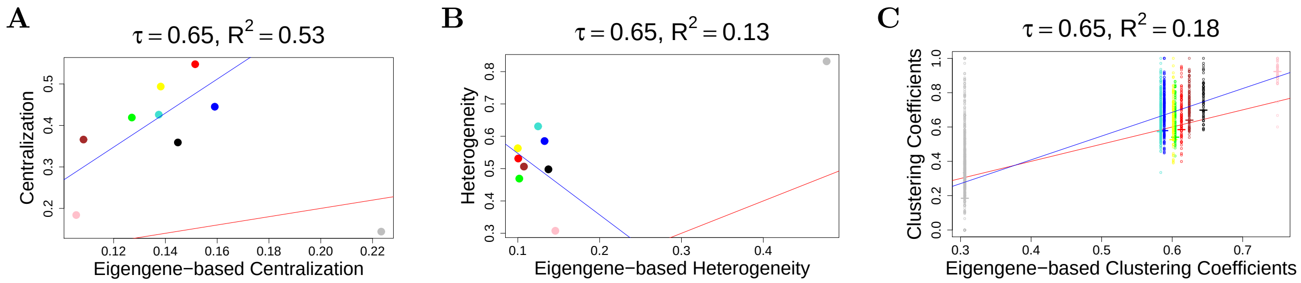

Figure 24: This figure is analogous to Figure 6 in the main article, corresponding to an unweighted network constructed with a hard threshold of $\tau = 0.65$. It illustrates Observation 2 regarding the relationship between network concepts (y-axis) and their eigengene-based analogs (x-axis) in the mouse data. Each point corresponds to a module. Figure A: Centralization (y-axis) versus eigengene-based Centralization$_E$ (x-axis); analogous plots for Figure B: Heterogeneity; Figure C: clustering coefficient. The blue line is the regression line through the points representing proper modules (i.e., the grey, non-module genes are left out). While the red reference line (slope 1, intercept 0) does not always fit well, we observe high squared correlations R^2 between network concepts and their analogs. Since the grey point corresponds to the genes outside properly defined modules, we did not include it in calculations.

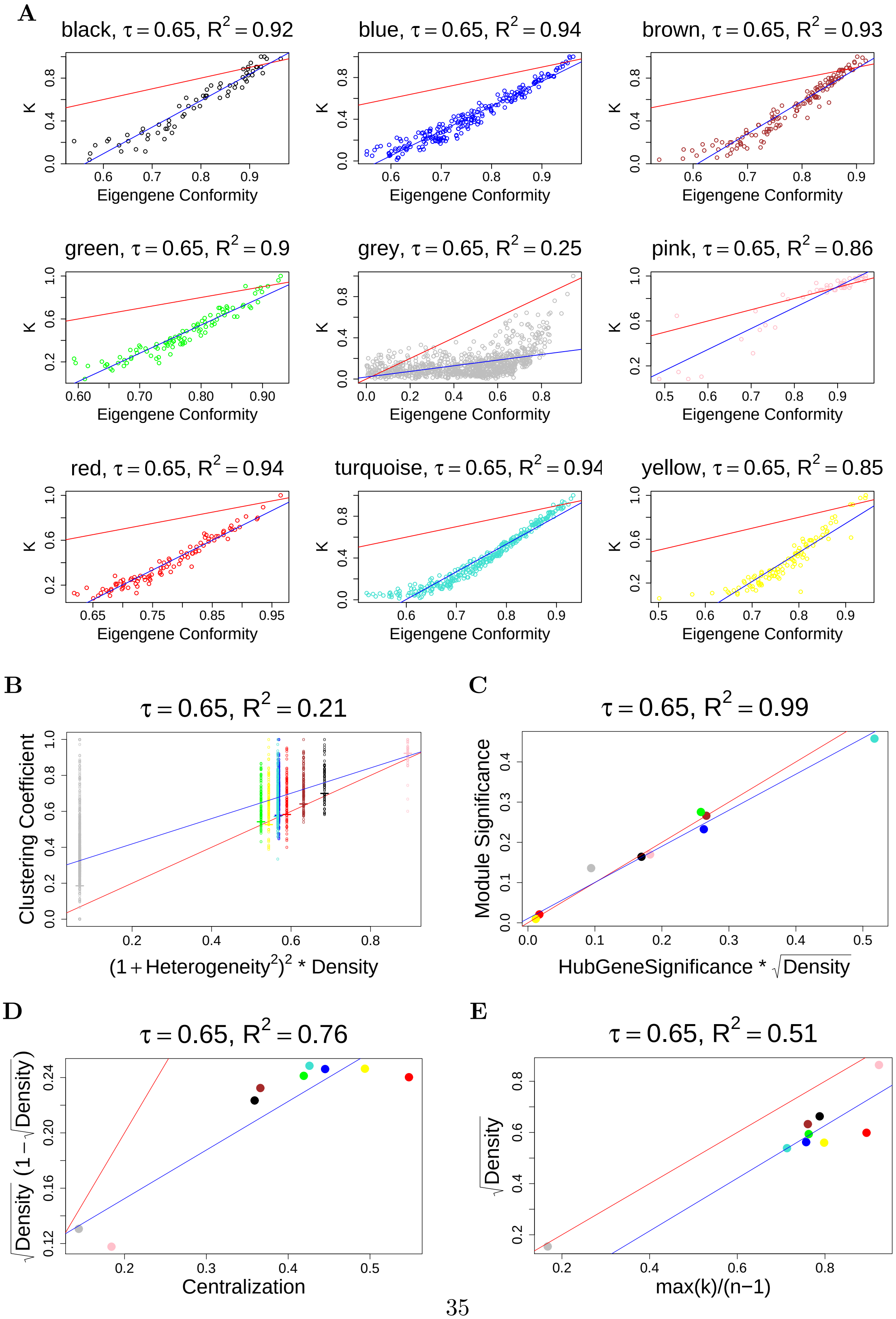

Figure 25: This figure is analogous to Figure 8 in the main article, corresponding to an unweighted network constructed with a hard threshold of $\tau = 0.65$. It illustrates Observation 3 regarding the relationships among network concepts.

12 Unweighted Gene Co-Expression Network Results for $\tau = 0.5$

Table 10: Values of network concepts for an unweighted network constructed with a hard threshold of $\tau = 0.5$.

Module	black	blue	brown	green	grey	pink	red	turquoise	yellow
Size ($n^{(q)}$)	67	215	139	98	920	53	96	308	105
Eigengene Factorizability ($EF(X^{(q)})$)	0.969	0.964	0.977	0.967	0.553	0.97	0.975	0.972	0.975
$VarExplained(\boldsymbol{E}^{(q)})$	0.633	0.58	0.62	0.599	0.25	0.697	0.608	0.576	0.596
$max(a_{e,i})$	0.964	0.962	0.917	0.929	0.945	0.965	0.965	0.933	0.943
$Density$	0.791	0.685	0.802	0.757	0.137	0.9	0.783	0.701	0.771
$Density_E$	0.63	0.571	0.615	0.598	0.203	0.732	0.607	0.568	0.596
$Centralization$	0.216	0.318	0.194	0.238	0.313	0.104	0.221	0.275	0.223
$Centralization_E$	0.145	0.159	0.108	0.127	0.223	0.106	0.151	0.137	0.138
$Heterogeneity$	0.203	0.261	0.19	0.197	0.481	0.151	0.185	0.258	0.2
$Heterogeneity_E$	0.137	0.133	0.108	0.102	0.477	0.146	0.101	0.125	0.1
$Mean(ClusterCoef)$	0.875	0.818	0.872	0.838	0.462	0.943	0.85	0.821	0.847
$ClusterCoef_E$	0.644	0.589	0.624	0.604	0.306	0.749	0.613	0.584	0.602
$ModuleSignif$	0.164	0.233	0.266	0.276	0.136	0.17	0.0208	0.458	0.00952
$HubGeneSignif$	0.21	0.343	0.317	0.331	0.379	0.192	0.0235	0.639	0.0128

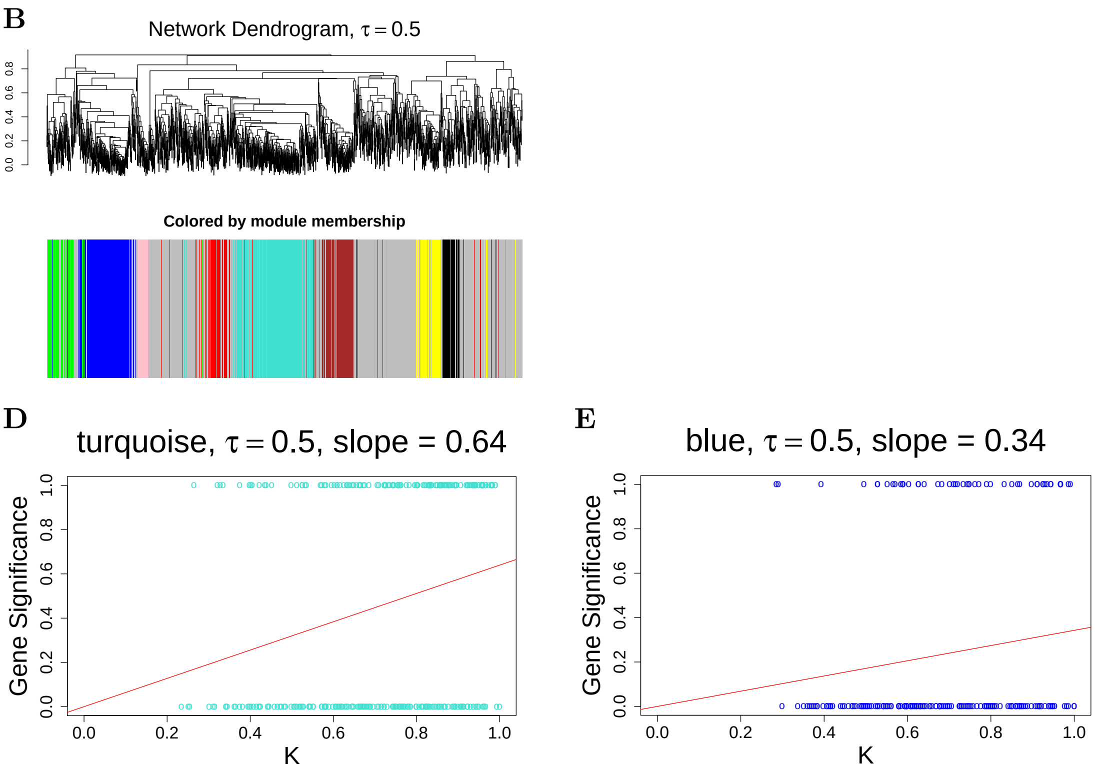

Figure 26: This figure is analogous to Figure 3 in the main article. The only difference is that we use a different dissimilarity for constructing the cluster tree in Figure B. Specifically, we use the topological overlap matrix based dissimilarity corresponding to an unweighted network constructed with $\tau = 0.5$. Figure B depicts the hierarchical cluster tree of genes. Modules correspond to branches of the tree. The branches and module genes are assigned a color as can be seen from the color-bands underneath the tree. Grey denotes genes outside of proper modules. Figure C would be identical to the case of $\beta = 1$, and thus is omitted here. Figures D and E show scatter plots of gene significance GS (y-axis) versus scaled connectivity K (x-axis) in the turquoise and blue module, respectively. The underlying gene significance is defined as the knock out essentiality. The hub gene significance is defined as the slope of the red line, which results from a regression model without an intercept term.

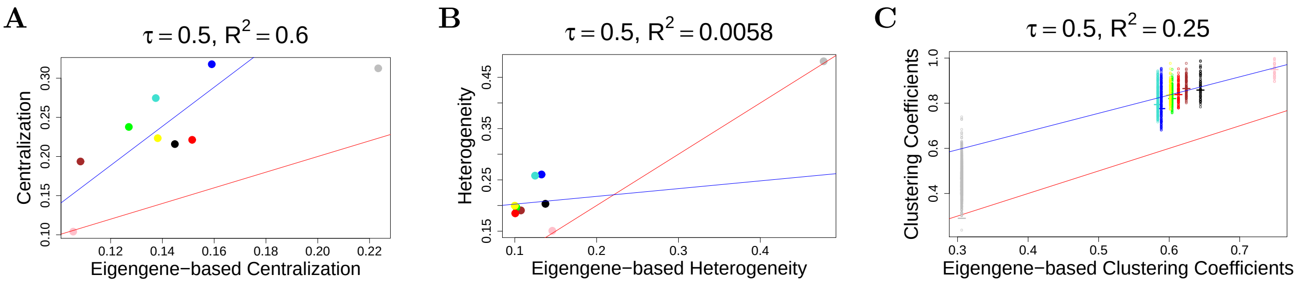

Figure 27: This figure is analogous to Figure 6 in the main article, corresponding to an unweighted network constructed with a hard threshold of $\tau = 0.5$. It illustrates Observation 2 regarding the relationship between network concepts (y-axis) and their eigengene-based analogs (x-axis) in the mouse data. Each point corresponds to a module. Figure A: Centralization (y-axis) versus eigengene-based Centralization$_E$ (x-axis); analogous plots for Figure B: Heterogeneity; Figure C: clustering coefficient. The blue line is the regression line through the points representing proper modules (i.e., the grey, non-module genes are left out). While the red reference line (slope 1, intercept 0) does not always fit well, we observe high squared correlations R^2 between network concepts and their analogs. Since the grey point corresponds to the genes outside properly defined modules, we did not include it in calculations.

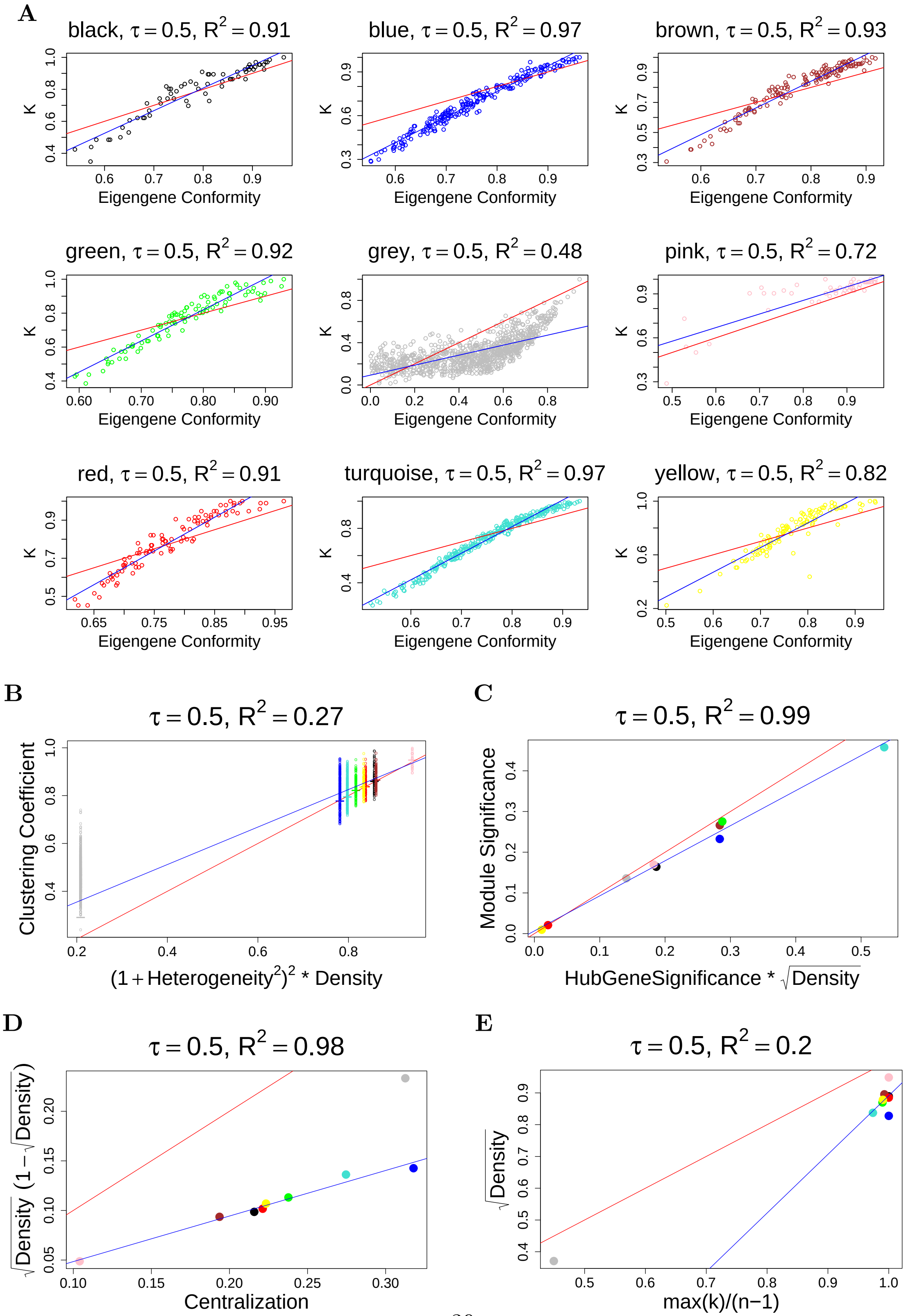

Figure 28: This figure is analogous to Figure 8 in the main article, corresponding to an unweighted network constructed with a hard threshold of $\tau = 0.5$. It illustrates Observation 3 regarding the relationships among network concepts.

References

Carlson, M., Zhang, B., Fang, Z., Mischel, P., Horvath, S. and Nelson, S. F. (2006). Gene connectivity, function, and sequence conservation: Predictions from modular yeast co-expression networks. *BMC Genomics*, **7**(40).

Spellman, P. T., Sherlock, G., Zhang, M. Q., Iyer, V. R., Anders, K., Eisen, M. B., Brown, P. O., Botstein, D. and Futcher, B. (1998). Comprehensive Identification of Cell Cycle-regulated Genes of the Yeast Saccharomyces cerevisiae by Microarray Hybridization. *Mol. biol. cell*, **9**(12), 3273–3297.
