# Supplementary material for: Geometric Interpretation of Gene Coexpression Network Analysis
Source: PLoS Comput Biol. 2008 Aug 15;4(8):e1000117. doi: 10.1371/journal.pcbi.1000117 (PMC2446438; doi:10.1371/journal.pcbi.1000117)
Supplement: Text S4 — Brain Cancer Network Comprised of 500 Prognostic Genes. Here we analyze a brain cancer network comprised of the 500 genes with highest absolute correlation with brain cancer survival time. The results illustrate that our theoretical results also apply to small networks comprised of sample trait related genes. The robustness analysis illustrates how the results change with regard to different network construction methods. (0.38 MB PDF) [file pcbi.1000117.s004.pdf]

Text S4: Brain Cancer Network Comprised of the 500 Genes with Highest Absolute Correlation with Brain Cancer Survival Time

Jun Dong and Steve Horvath*

Dept. of Human Genetics, David Geffen School of Medicine, UCLA
Dept. of Biostatistics, School of Public Health, UCLA
*Correspondence: shorvath@mednet.ucla.edu

Abstract

This is a Supplement of the article "Geometric Interpretation of Gene Co-Expression Network Analysis". Here we study a brain cancer network comprised of the 500 genes with highest absolute correlation with brain cancer survival time. These genes are a subset of the 3600 genes in our brain cancer data. Our theoretical results also apply to networks comprised of genes that are highly correlated with a sample trait, which is illustrated here.

1 Brain Cancer Gene Co-expression Network Application

In this Supplement, we use a weighted gene co-expression network that was constructed on the basis of the 500 genes with highest absolute correlation with brain cancer survival time in our brain cancer data. We defined 6 modules as branches of an average linkage hierarchical cluster tree (dendrogram), see Figure reffig:overview(a) in the main article. Module membership in the 5 'proper' modules is color-coded by turquoise, blue, brown, yellow and green. Grey denotes the color of genes that were not grouped into any of the 6 proper modules. To allow for a comparison, we also report results for the 'improper' module comprised of grey, non-module genes.

We have constructed weighted networks with $\beta = 1$ and 6, and unweighted networks with $\tau = 0.5$. For the unweighted networks, we use the eigengene-based network concepts of weighted networks with $\beta = 1$ for demonstration purposes.

Table 1: Values of Network Concepts in Weighted Gene Co-Expression Module Networks (brain cancer data). This table is analogous to Table 2 in the main article.

Module	blue	brown	green	grey	turquoise	yellow
Size ($n^{(q)}$)	159	47	17	10	247	20
Eigengene Factorizability ($EF(X^{(q)})$)	0.886	0.951	0.99	0.934	0.984	0.945
$VarExplained(\boldsymbol{E}^{(q)})$	0.452	0.576	0.779	0.605	0.623	0.572
$max(a_{e,i})$	0.963	0.949	0.972	0.954	0.981	0.885
$Density$	0.412	0.546	0.761	0.527	0.604	0.545
$Density_E$	0.398	0.566	0.823	0.633	0.608	0.597
$Centralization$	0.187	0.151	0.0959	0.198	0.158	0.114
$Centralization_E$	0.214	0.163	0.0975	0.208	0.16	0.116
$Heterogeneity$	0.294	0.189	0.0789	0.264	0.17	0.0919
$Heterogeneity_E$	0.378	0.197	0.0747	0.251	0.171	0.087
$Mean(ClusterCoef)$	0.491	0.587	0.771	0.61	0.64	0.554
$ClusterCoef_E$	0.517	0.598	0.784	0.643	0.641	0.576
$ModuleSignif$	0.195	0.222	0.287	0.252	0.212	0.332
$ModuleSignif_E$	0.151	0.212	0.285	0.216	0.205	0.331
$HubGeneSignif$	0.248	0.27	0.317	0.32	0.259	0.393
$HubGeneSignif_E$	0.232	0.271	0.315	0.272	0.259	0.389
$EigengeneSignif$	0.24	0.285	0.324	0.286	0.264	0.439

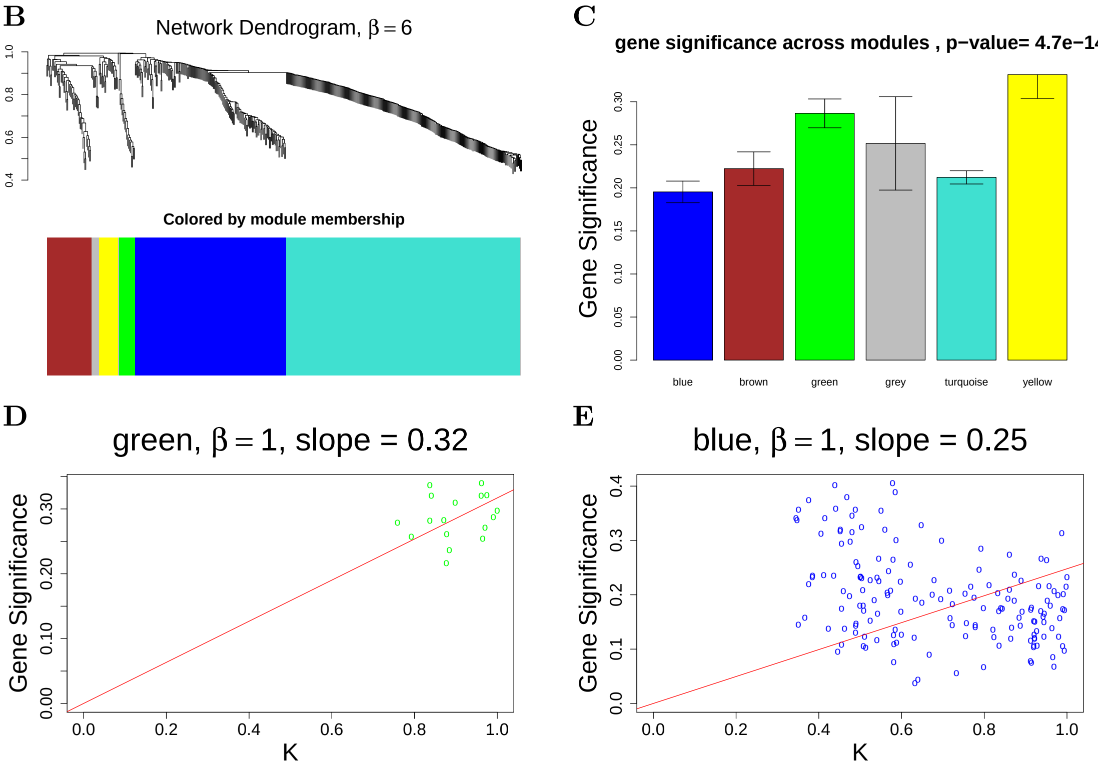

Figure 1: This figure is analogous to Figure 3 in the main article. Figure B depicts the hierarchical cluster tree of genes. Modules correspond to branches of the tree. The branches and module genes are assigned a color as can be seen from the color-bands underneath the tree. Grey denotes genes outside of proper modules. Figure C shows the module significance (average gene significance) of the modules. The underlying gene significance is defined with respect to the patient survival time. Figures D and E show scatter plots of gene significance GS (y-axis) versus scaled connectivity K (x-axis) in the green and blue module, respectively. The hub gene significance is defined as the slope of the red line, which results from a regression model without an intercept term.

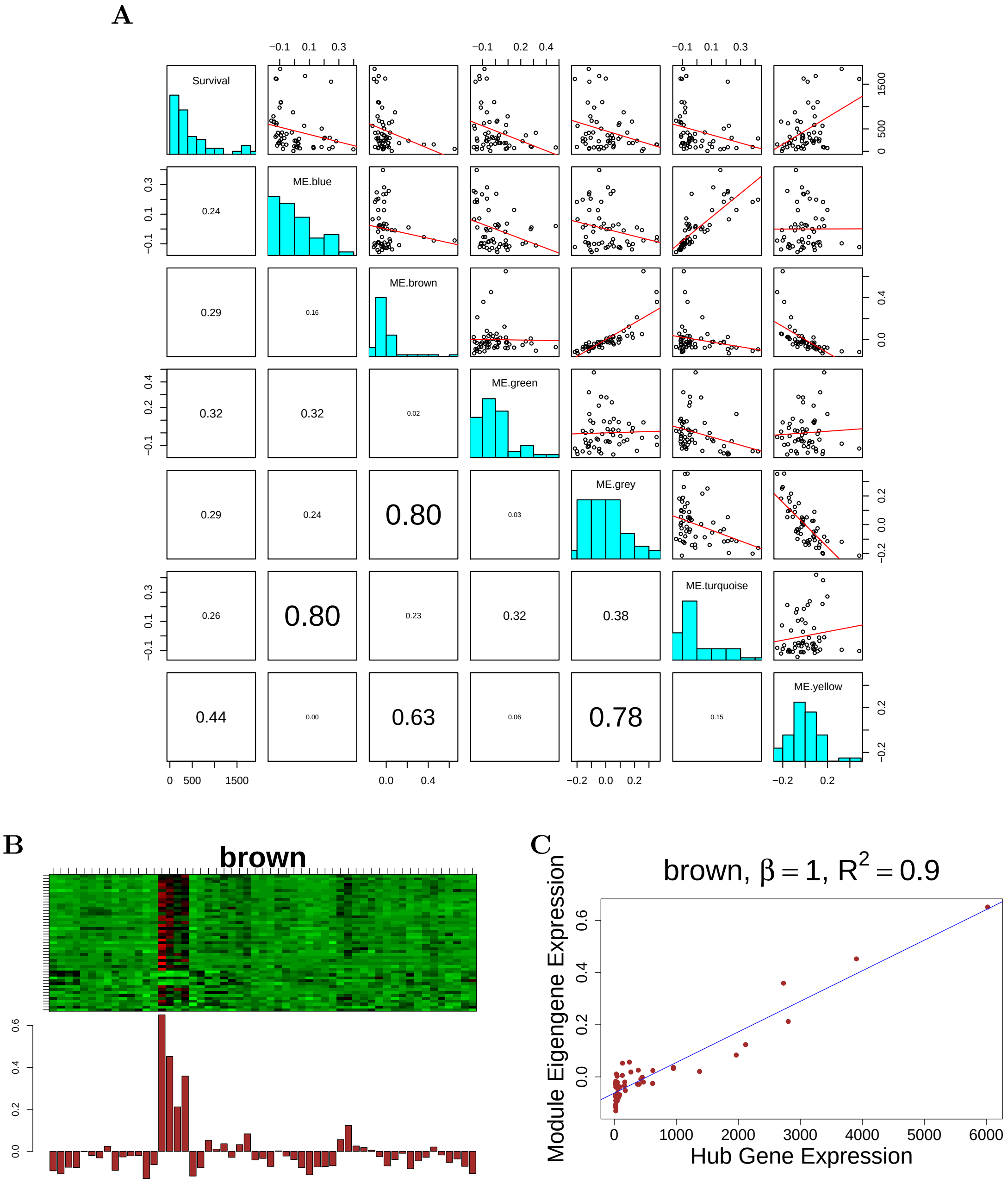

Figure 2: This figure is analogous to Figure 4 in the main article. Module eigengenes in the brain cancer gene co-expression network. Figure A depicts the pairwise scatter plots between the module eigengenes $\boldsymbol{E}^{(q)}$ of different modules and cancer survival time T. Each dot represents a microarray sample. ME.blue denotes the module eigengene $\boldsymbol{E}^{(blue)}$ of the blue module. Numbers below the diagonal are the absolute values of the corresponding correlations. Frequency plots (histograms) of the variables are plotted along the diagonal. Upper panel of Figure B: heat map plot of the brown module gene expression profiles (rows) across the microarray samples (columns). Red corresponds to high- and green to low- expression values. Since the genes of a module are highly correlated, one observes vertical bands. Lower panel of Figure B: the values of the components of the module eigengene (y-axis) versus microarray sample number (x-axis). Note that vertical bands of red (green) in the upper panel correspond to high (low) values of the eigengene in the lower panel. Figure C shows that the expression profile of the module eigengene (y-axis) is highly correlated with that of the most highly connected hub gene (x-axis). A linear regression line has been added.

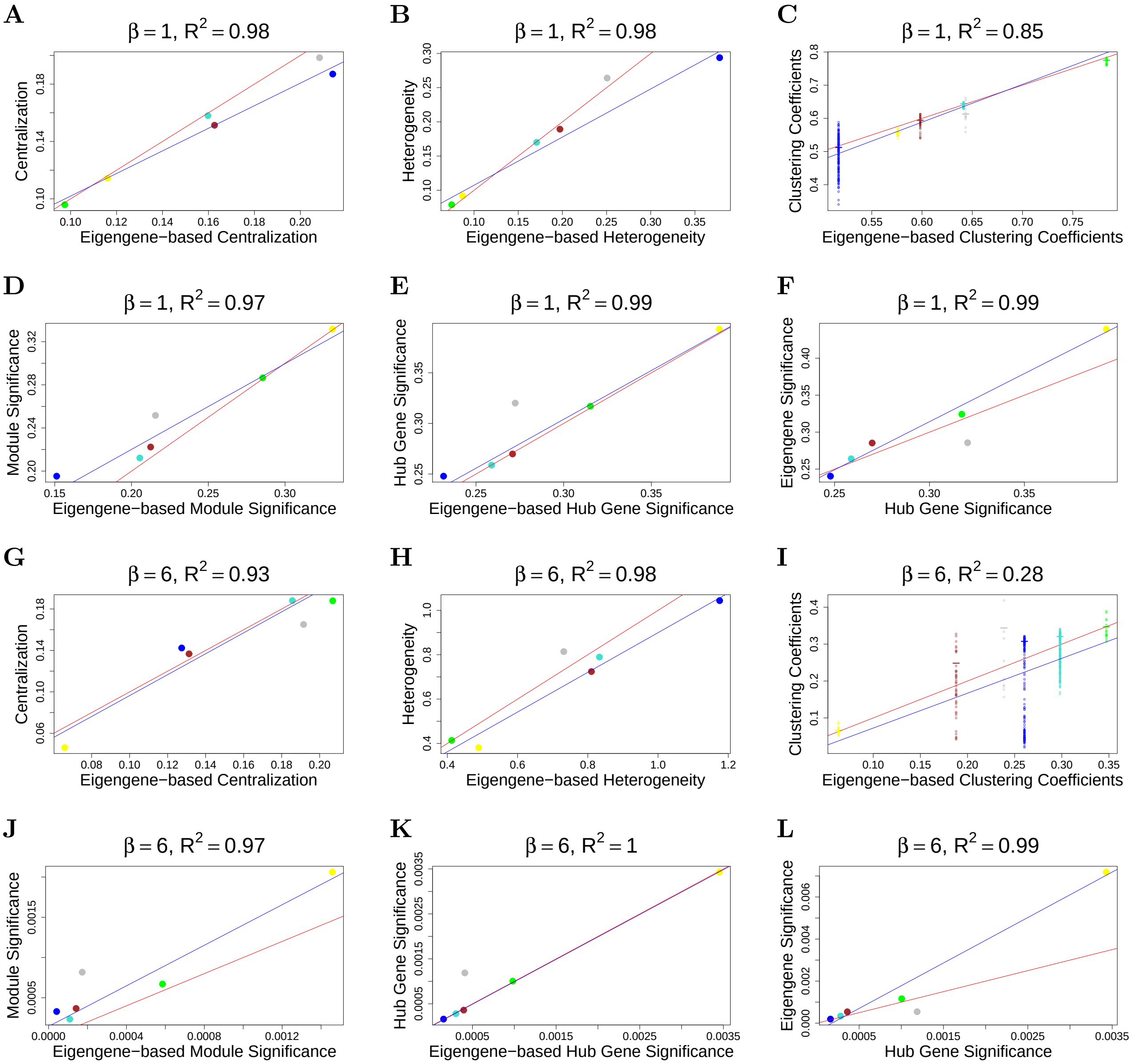

Figure 3: This figure is analogous to Figure 6 in the main article. Illustrating Observation 2 regarding the relationship between network concepts (y-axis) and their eigengene-based analogs (x-axis) in the brain cancer data. Each point corresponds to a module. Figures (A-F) and (G-L) correspond to a weighted network constructed with a soft threshold of $\beta = 1$ and $\beta = 6$, respectively. (A,G) Centralization (y-axis) versus eigengene-based Centralization$_E$ (x-axis); analogous plot for (B,H) Heterogeneity (C,I) clustering coefficient; (D,J) module significance; and (E,K) hub gene significance; Figures (F,L) illustrate Equation (13) in the main article regarding the relationship between eigengene significance and hub gene significance. The blue line is the regression line through the points representing proper modules (i.e., the grey, non-module genes are left out). While the red reference line (slope 1, intercept 0) does not always fit well, we observe high squared correlations R^2 between network concepts and their analogs. Since the grey point corresponds to the genes outside properly defined modules, we did not include it in calculations.

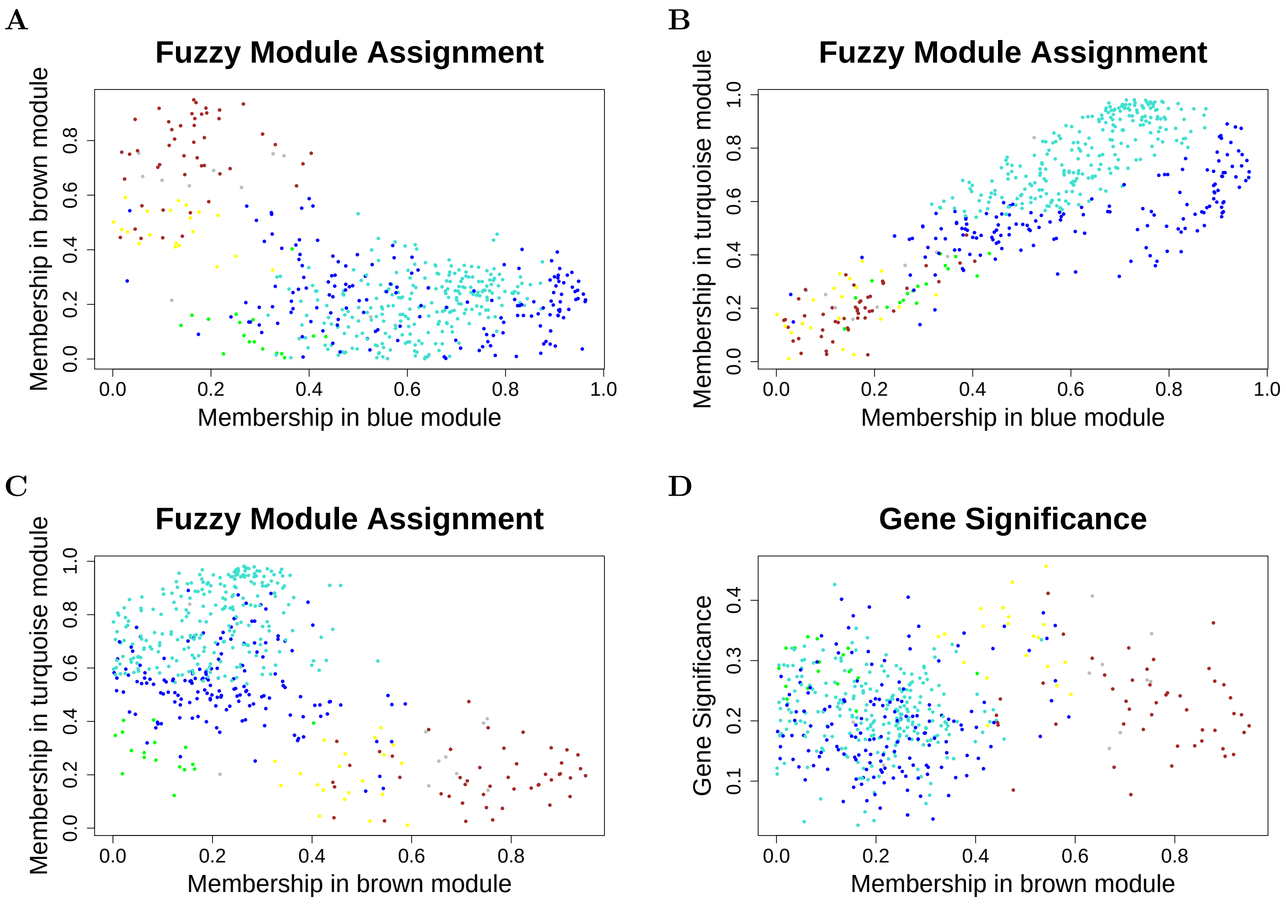

Figure 4: This figure is analogous to Figure 7 in the main article. A natural choice for a fuzzy measure of module membership is the generalized scaled connectivity measure $K^{(q)}_{cor,i} = |cor(\boldsymbol{x}_i, \boldsymbol{E}^{(q)})|$. Figure A shows the scatterplot of the brown module membership measure (y-axis) versus that of the blue module (x-axis). Note that grey dots corresponding to genes outside of properly defined modules may can be intermediate between module genes. Figure B shows the corresponding plot for blue versus turquoise module membership; Figure C shows brown versus turquoise module membership. Figure D shows the relationship between gene significance based on survival time (y-axis) and brown module membership (x-axis).

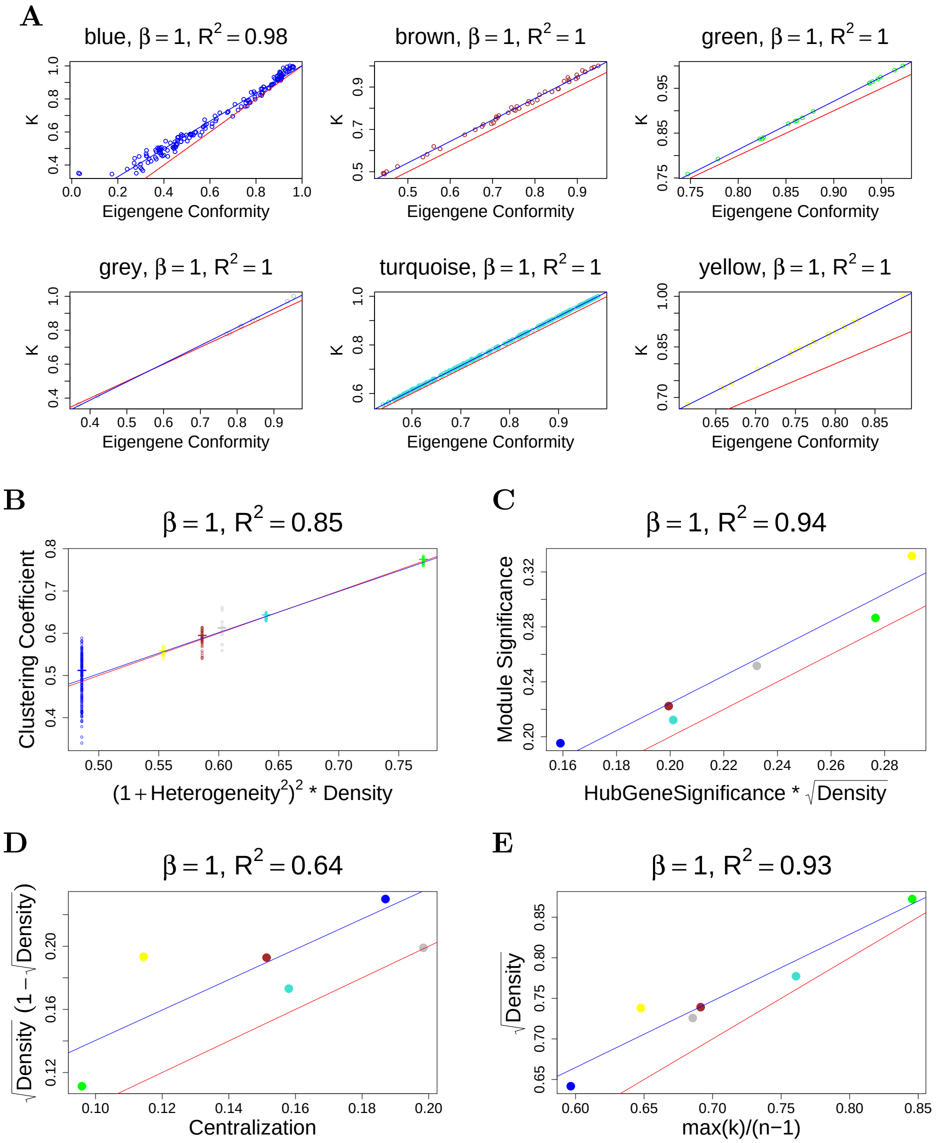

Figure 5: This figure is analogous to Figure 8 in the main article. It illustrates Observation 3 regarding the relationships among network concepts. Figure A illustrates Equation (33) regarding the relationship between scaled intramodular connectivity $K_i^{(q)}$ (y-axis) and eigengene conformity $a_{e,i}$ (x-axis). Each dot corresponds to a gene colored by its module membership. We find a high squared correlation R^2 even for the grey genes outside properly defined modules. Figure B illustrates Equation (31) regarding the relationship between the clustering coefficient and $(1 + Heterogeneity^2)^2 \times Density$. Again each dot represents a gene. The clustering coefficients of grey genes vary more than those of genes in proper modules. The short horizontal lines correspond to the mean clustering coefficient of each module. Figure C illustrates $ModuleSignif^{(q)} \approx \sqrt{Density^{(q)}} \times HubGeneSignif^{(q)}$ (Equation 37); here each dot corresponds to a module. Since the grey dot corresponds to genes outside of properly defined modules, we have excluded it from the calculation of the squared correlation R^2. Figure D illustrates $Centralization^{(q)} \approx \sqrt{Density^{(q)}}(1 - \sqrt{Density^{(q)}})$ (Equation 40); Figure E illustrates $\frac{k_{max}^{(q)}}{n^{(q)}-1} \approx \sqrt{Density^{(q)}}$ (Equation 38). A reference line (red) with intercept 0 and slope 1 has been added to each plot. The blue line is the regression line through the points representing proper modules (i.e., the grey, non-module genes are left out).

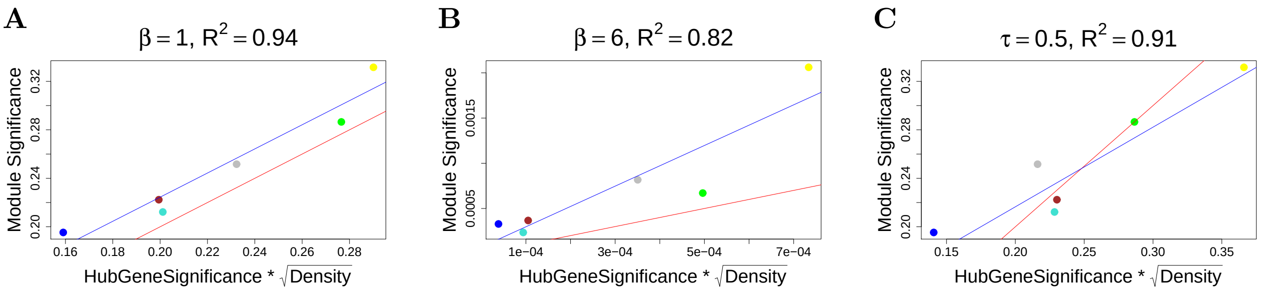

Figure 6: This figure is analogous to Figure 9 in the main article. It illustrates Equation (37) regarding the relationship between module significance (y-axis) and $\sqrt{Density^{(q)}} \times HubGeneSignif^{(q)}$ (x-axis). Points correspond to modules. The square of the correlation coefficient R^2 was computed without the grey, improper module. The figures correspond to weighted networks constructed with soft thresholds $\beta = 1$ and $\beta = 6$, and an unweighted network that results from thresholding the correlation matrix at $\tau = 0.5$. Overall, we find that the reported relationship is quite robust with respect to our theoretical assumptions (e.g. factorizability). The blue line is the regression line through the points representing proper modules (i.e., the grey, non-module genes are left out). A reference line with slope 1 and intercept 0 is shown in red.
